# Sequential and Opposing Activities of Wnt and BMP Coordinate Zebrafish Bone Regeneration

**DOI:** 10.1016/j.celrep.2014.01.010

**Published:** 2014-01-31

**Authors:** Scott Stewart, Alan W. Gomez, Benjamin E. Armstrong, Astra Henner, Kryn Stankunas

**Affiliations:** 1 Institute of Molecular Biology, University of Oregon, Eugene, OR 97403, USA; 2 Department of Biology, University of Oregon, Eugene, OR 97403, USA

## Abstract

Zebrafish fully regenerate lost bone, including after fin amputation, through a process mediated by dedifferentiated, lineage-restricted osteoblasts. Mechanisms controlling the osteoblast regenerative program from its initiation through reossification are poorly understood. We show that fin amputation induces a Wnt/β-catenin-dependent epithelial to mesenchymal transformation (EMT) of osteoblasts in order to generate proliferative Runx2^+^ preosteoblasts. Localized Wnt/β-catenin signaling maintains this progenitor population toward the distal tip of the regenerative blastema. As they become proximally displaced, preosteoblasts upregulate sp7 and subsequently mature into re-epithelialized Runx2^−^/sp7^+^ osteoblasts that extend preexisting bone. Auto-crine bone morphogenetic protein (BMP) signaling promotes osteoblast differentiation by activating *sp7* expression and counters Wnt by inducing Dickkopf-related Wnt antagonists. As such, opposing activities of Wnt and BMP coordinate the simultaneous demand for growth and differentiation during bone regeneration. This hierarchical signaling network model provides a conceptual framework for understanding innate bone repair and regeneration mechanisms and rationally designing regenerative therapeutics.

## INTRODUCTION

Among mammalian organs, bones have unusually effective repair mechanisms, as demonstrated by continuous bone remodeling throughout life and the scarless healing of some fractures. Still, traumatic injuries or disease frequently exceed the innate regenerative capacities of human bone (Dimitriou et al., 2011). Envisioned restorative bone therapies include, for example, engineered cell scaffolds combined with bone grafts and the delivery of manipulated stem cells including patient-specific induced pluripotent stem cells. An appealing alternative is to recapitulate mechanisms observed in animals with remarkable capacities for self-repair, including fish and salamanders.

Osteoblasts are specialized bone producing cells that deposit a unique extracellular matrix, the osteoid, that mineralizes to form mature bone. Two transcription factors, Runx2 and sp7/Osterix (Osx), are key determinants of the osteoblast lineage (Long, 2012). *Runx2*-null mice lack all ossification (Ducy et al., 1997; Komori et al., 1997; Otto et al., 1997). Similarly, *Sp7*-deficient mice do not form bone due to a failure of osteoblasts to differentiate (Nakashima et al., 2002). *Runx2* functions upstream of *Sp7; Runx2*-deficient mice fail to express *Sp7*, whereas *Sp7*-null mice retain *Runx2* expression (Nakashima et al., 2002). Transcriptional control of *Runx2* and *Sp7* is mediated via cell signaling including by the Wnt and bone morphogenetic protein (BMP) pathways, both central components of bone developmental regulatory networks (Long, 2012).

Wnts are secreted proteins that function in many biological processes, including development and cancer (Clevers and Nusse, 2012). Wnts bind to Frizzled (Fz) and Lrp5/6 coreceptors culminating in the stabilization and nuclear translocation of β-catenin. Nuclear β-catenin functions with Tcf/Lef transcription factors to affect gene expression (Clevers and Nusse, 2012). Given Wnt’s signaling potency in influencing cell behavior and lineage decisions, the pathway is under tight control (Clevers and Nusse, 2012). For instance, the Dickkopf family of secreted Wnt antagonists moderate Wnt activity by binding to and blocking Lrp5/6 (Mao et al., 2001). Deletion of mouse β-catenin in early mesenchymal precursors results in loss of *Runx2* and *Sp7* expression and a corresponding failure of bone formation, suggesting osteogenesis requires Wnt/β-catenin signaling (Day et al., 2005; Glass et al., 2005; Gong et al., 2001; Hill et al., 2005; Hu et al., 2005; Rodda and McMahon, 2006).

The BMP pathway is an additional key regulator of osteogenesis. Secreted BMPs bind and activate specific BMP serine/threonine kinase receptors (BMPRs). BMPRs phosphorylate Smad1/5/8 transcription factors, inducing their translocation to the nucleus where they activate downstream genes. BMPs are required for osteoblast-lineage commitment of cranial mesenchyme (Abzhanov et al., 2007), and loss of both *Bmp2* and *Bmp4* results in a severe defect in osteoblast differentiation likely due to a failure of *Runx2*-expressing cells to upregulate *Sp7* (Bandyopadhyay et al., 2006). These and earlier studies led to the development of recombinant BMPs as therapies to augment bone healing, albeit with debated clinical benefits (Garrison et al., 2010).

Mammalian bone fracture repair is mediated by bone-marrow-derived mesenchymal stem cells that differentiate into osteoblasts and subsequently produce remineralized bone (Dimitriou et al., 2011). Several signaling pathways, including Wnt and BMP, have been implicated in these repair processes, but their modes of action at the cellular and molecular level are largely unknown. For example, it is unclear whether Wnts and BMPs influence discrete osteoprogenitor populations or how expression of *Runx2* and *Sp7* the master regulators of osteogenesis, are coordinated during the repair process (Dimitriou et al., 2011). Even more confounding are observations that, whereas both Wnts and BMPs positively influence bone repair, the pathways appear to function in opposition to one another (Minear et al., 2010a).

Adult zebrafish fins comprise multiple organized cell types, including osteoblasts, fibroblasts, endothelial cells, neurons, and epidermal cells. Each of these cell types regenerate in concert after resection to perfectly restore the entire fin within 2–3 weeks (Gemberling et al., 2013). The bony rays, or lepidotrichia, that characterize fish fins are ossified spokes lined with bone forming osteoblasts. Lineage-tracing studies demonstrate that replacement osteoblasts are derived from preexisting uni-potent osteoblasts (Knopf et al., 2011; Singh et al., 2012; Sousa et al., 2011; Stewart and Stankunas, 2012; Tu and Johnson, 2011), although an unknown population can substitute if needed (Singh et al., 2012). These observations indicate that mature osteoblasts respond to injury by acquiring progenitor cell properties, a process known as dedifferentiation. These fate restricted cells then populate lateral regions of the regenerative blastema, specialized mesenchymal tissue formed upon amputation. Subsequently, by poorly understood mechanisms, the osteoblast lineage cells undergo coordinated proliferation, differentiation, and morphogenesis to form replacement bone.

Fin regeneration is accompanied by upregulation of transcripts associated with osteogenesis including *runx2a*, *runx2b*, and *sp7* (Knopf et al., 2011; Smith et al., 2006; Sousa et al., 2011). Transgenic lines utilizing fish *sp7* promoter/enhancer elements indicate that fin osteoblasts distal to the amputation site express *sp7*, whereas cells below the amputation site downregulate *sp7* expression (DeLaurier et al., 2010; Knopf et al., 2011; Singh et al., 2012). Similarly, human *Runx2* regulatory elements drive reporter gene expression in cells distal to the amputation site (Knopf et al., 2011). Although the Wnt/β-catenin pathway is required for fin regeneration and components of the Wnt pathway are expressed in the regenerating fin (Kawakami et al., 2006; Poss et al., 2000; Stoick-Cooper et al., 2007), it remains unknown which cell types participate in Wnt/β-catenin signaling or how the pathway promotes regeneration. Similarly, BMP gene expression patterns and overexpression of the BMP inhibitor Chordin implicate the BMP pathway in fin osteoblast differentiation (Smith et al., 2006). However, it is unresolved how BMP promotes bone differentiation or even when and where BMP signaling is active during regeneration.

Here, we examine mechanisms of regenerative osteogenesis in the zebrafish caudal fin. Upon fin amputation, Runx2^+^ preosteoblasts accumulate in the blastema following a Wnt/β-catenin dependent epithelial-to-mesenchymal transformation (EMT) of osteoblasts lining the damaged bone. Distal Wnt signaling continuously maintains Runx2^+^ preosteoblasts, whereas their differentiation into mature, sp7^+^ osteoblasts is promoted by autocrine BMP signaling. By inducing secreted Wnt antagonists, including *dkk1b*, BMP facilitates negative crosstalk between differentiating osteoblasts and preosteoblasts. This straightforward signaling network produces a hierarchically and spatially organized osteoblast lineage that balances a simultaneous need for bone growth and differentiation until fin regeneration is complete.

## RESULTS

### Runx2 and sp7 Define a Hierarchical Organization of Osteoblast Lineage Cells during Fin Regeneration

**Figure 1:**
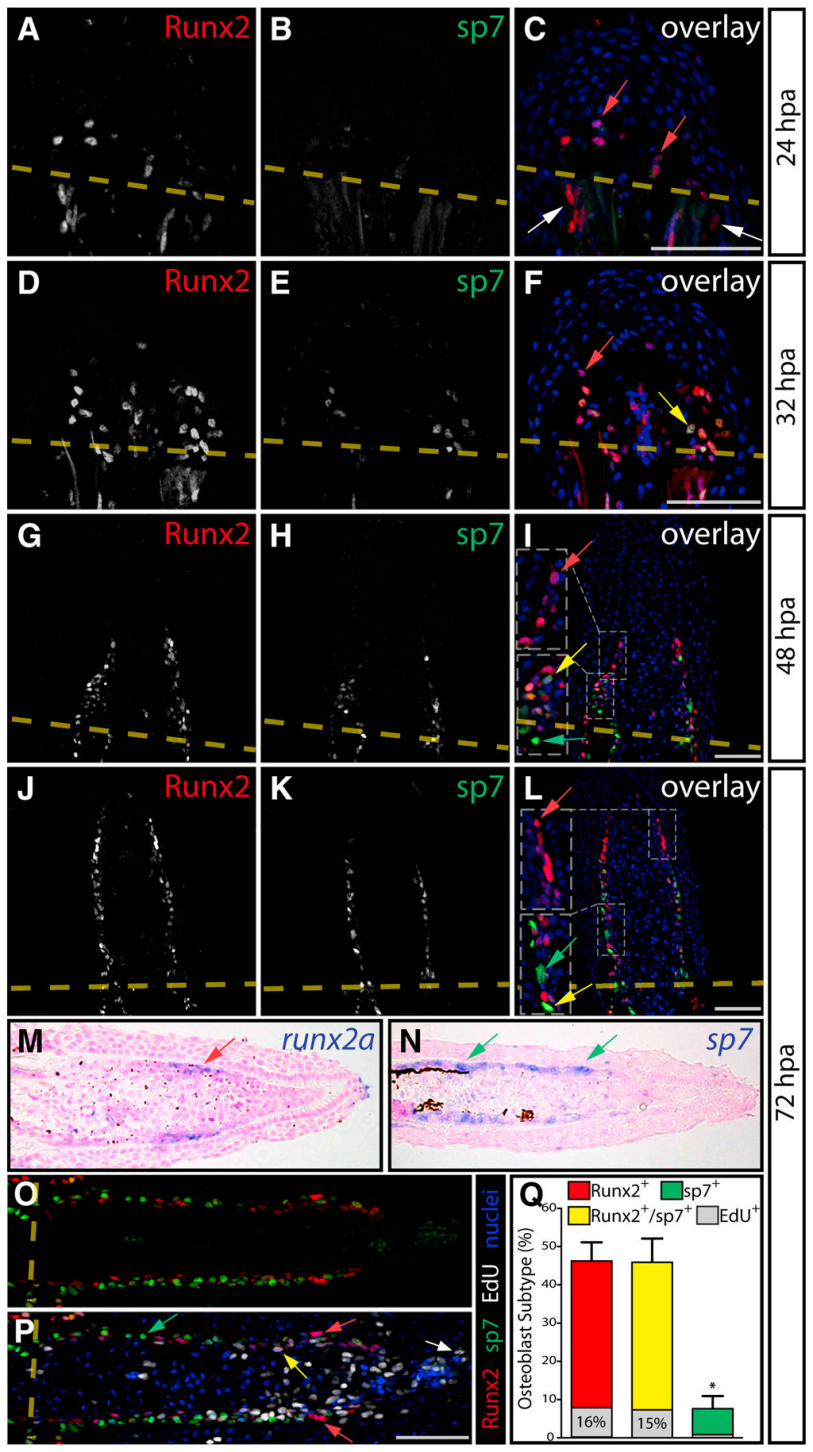
Runx2 and sp7 Define Distinct Populations of Preosteoblasts in Regenerating Fins (A–L) Runx2 (red) and sp7 (green) immunostaining at 24 (A–C), 32 (D–F), 48 (G–I), and 72 (J–L) hr post-amputation (hpa) on longitudinal caudal fin sections. Images are maximum intensity projections of confocal z-stacks. Amputation planes are indicated with a dashed yellow line, white arrows point to Runx2^+^ cells proximal to the amputation site, red arrows indicate Runx2^+^ cells distal to the amputation site, yellow arrows denote Runx2^+^/sp7^+^cells, and green arrows show sp7^+^ cells. For (I) and (L), regions bound by dashed white boxes are shown in higher magnification in inset panels. (M and N) RNA in situ hybridizations showing *runx2a* and *sp7* expression on fin sections harvested 72 hpa. Red and green arrows point to lateral blastema cells expressing *runx2a* and *sp7*, respectively. (O and P) Immunostaining showing Runx2 (red) and sp7 (green) expression (O) and incorporation of EdU (P, white, 6 hr pulse) on 72 hpa fin sections. The white arrow points to an extreme distally located EdU^+^ blastema cell. The yellow and green arrows indicate Runx2^+^/EdU^+^ and sp7^+^/EdU^−^ cells, respectively. Red arrows show high Runx2^+^/EdU^−^ cells. In all overlay images, Hoechst-stained nuclei are shown in blue. The scale bars represent 50 μm. (Q) Quantitation of osteoblast subtypes and EdU incorporation at 72 hpa. Bars show the mean percentile representation of osteoblast subtypes on comparable sections (n = 12 rays, compiled from >4 fish). Error bars are one SD from the mean, and significant p values are indicated with an asterisk (p < 10^−5^, Student’s t tests comparing either Runx2^+^ or Runx2^+^/sp7^+^ populations to sp7^+^ cells). The proportion of each cell population that incorporated EdU is indicated by the extent of gray shading relative to the bar’s height. Fewer sp7^+^ relative to Runx2^+^ cells incorporated EdU (p < 0.03, one-tailed Fisher’s exact test, n = 547 Runx2^+^ and 75 sp7^+^ cells).

We immunostained paraffin-sectioned fins with antibodies directed against the osteogenic transcription factors, Runx2 and sp7, at various times postamputation. At 24 hr postamputation (hpa), osteoblasts lining preexisting bone adjacent to the amputation site upregulated expression of Runx2. Further, Runx2, but not sp7, expressing cells were found distal to the amputation site distributed in the mesenchymal tissue of the forming blastema (Figures 1A–1C). At 32 hpa, sp7 expression was induced in select Runx2^+^ mesenchymal cells (Figures 1D–1F). By 48 hpa, osteoprogenitors became more linearly organized adjacent to the basal epidermis with Runx2^−^/sp7^+^ cells first becoming detected near the amputation site (Figures 1G–1I). At 72 hpa, the osteoblast lineage was highly organized along the proximal-distal axis (Figures 1J–1L). Runx2^+^ cells were always the most distally localized population and were neighbored by Runx2^+^/sp7^+^ cells. In contrast, sp7^+^ cells were concentrated near the amputation site. Similarly, *runx2a* and *sp7* transcripts were enriched at the distal and proximal ends of the blastema, respectively (Figures 1M and 1N).

We quantified osteoblast subtypes and their proliferation state by delivering 5-ethynyl deoxyuridine (EdU) to label cells in S phase 6 hr prior to harvesting fins at 72 hpa. In stained section fins, we observed robust EdU labeling in the distal blastema mesenchyme but only rarely in the epidermis (Figures 1O and 1P), in agreement with a previous report (Smith et al., 2006). Runx2^+^ and Runx2^+^/sp7^+^ cells were found in equal proportion (Figures 1O, 1P, and 1Q, ~45%) with significantly fewer sp7^+^ cells (Figure 1Q, ~8%, p < 10^−5^). More Runx2^+^ cells incorporated EdU compared to sp7^+^ cells (16% versus 7%) (Figure 1Q, p < 0.03). We also routinely saw clusters of several high-expressing Runx2^+^ cells that were EdU^−^, suggesting there are distinct proliferative and nonproliferative Runx2^+^ preosteoblasts (Figure 1P, red arrows). The temporal and spatial patterns of Runx2 and sp7 expression and osteoblast-subtype proliferation demonstrate a hierarchical organization of the osteoblast lineage in regenerating fins. Renewing Runx2^+^ preosteoblasts at the leading edge of the fin beget proliferative Runx2^+^/sp7^+^-differentiating cells. These cells subsequently mature into relatively quiescent sp7^+^ osteoblasts that append to progressively elongating bone.

### A twist2-Associated Epithelial-to-Mesenchymal Transition Generates Preosteoblasts

**Figure 2:**
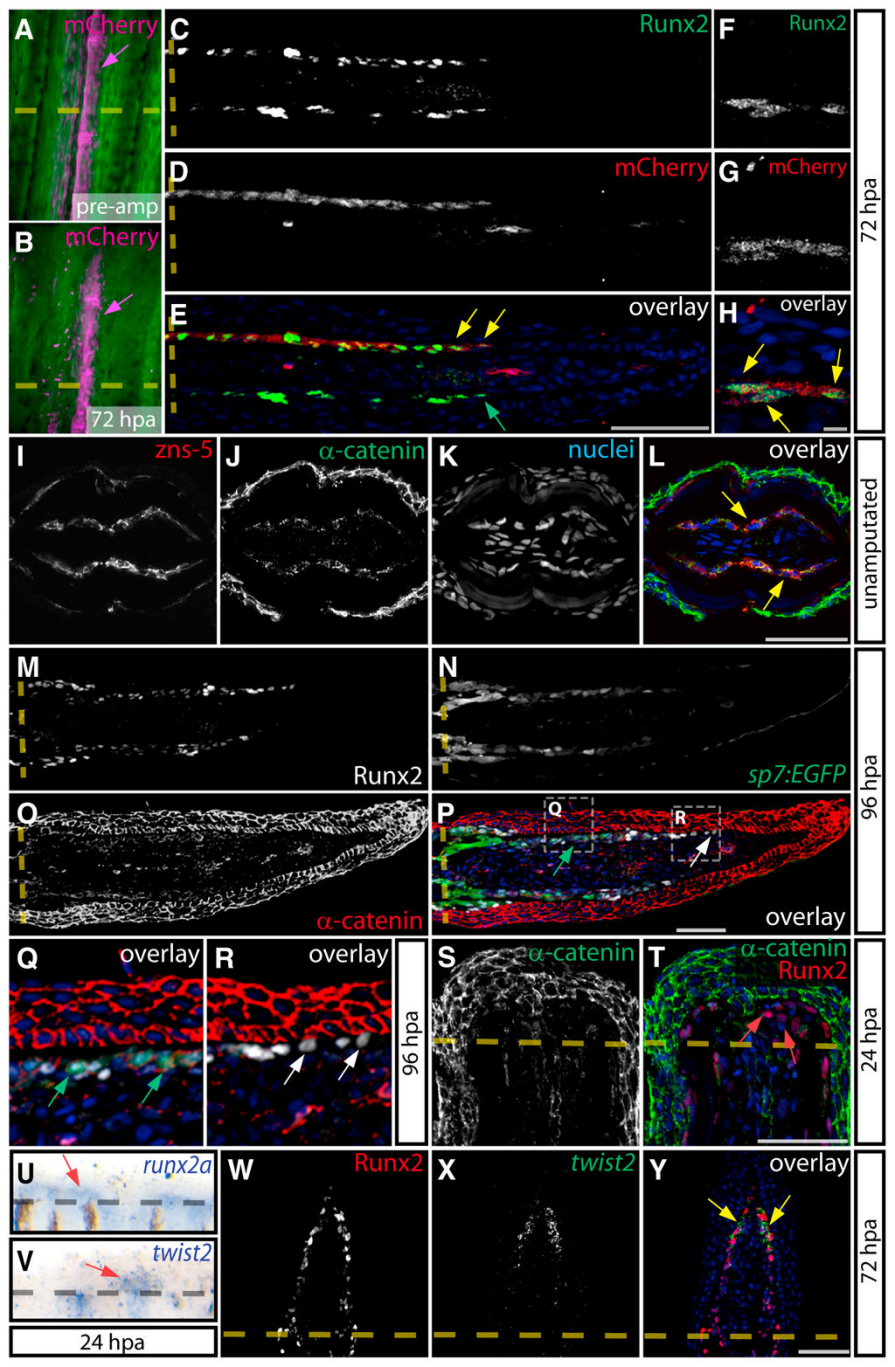
Dual Epithelial/Mesenchymal Nature of Osteoblasts during Fin Regeneration (A–H) Whole-mount imaging of a mosaic bone ray before amputation (A) and at 72 hpa (B) showing osteoblasts permanently labeled by Cre-mediated expression of mCherry (magenta, indicated by magenta arrows). Sections of the same ray at 72 hpa immunostained for Runx2 (C and F) and mCherry (D and G). Overlays are shown in (E) and (H). Yellow arrows show distal mCherry^+^/Runx2^+^ cells, and the green arrow points to a mCherry^−^/Runx2^+^ distal osteoblast (C–H). Amputation sites are indicated with a dashed line. (F)–(H) are high-magnification images of another section through the same ray in (C)–(E). (I–L) Antibody-stained transverse sections of nonregenerating fins showing α-catenin (green) and zns-5 (red). Yellow arrows indicate epithelial α-catenin^+^/zns-5^+^ osteoblasts. (M–R) A section from a 96 hpa *Tg(sp7:EGFP)* fin showing Runx2^+^ (white), *sp7:EGFP* (green), and α-catenin (red) expression. Regions bounded by dashed white boxes in (P) are shown at higher magnification in (Q) and (R). White arrows point to Runx2^+^ preosteoblasts and green arrows show *sp7:EGFP*^+^ osteoblasts with membrane-localized α-catenin. (S and T) Expression of Runx2 (red) and α-catenin (green) on 24 hpa sections. Red arrows indicate Runx2^+^/α -catenin^−^ mesenchymal cells. (U and V) Whole-mount RNA in situ hybridizations of *runx2a* (U) and *twist2* (V) at 24 hpa. Red arrows denote the specific expression of *runx2a* and *twist2* in regenerating tissue. (W–Y) Runx2 immunostaining (W, red) and *twist2* in situ hybridization (X, green) is shown on 72 hpa frozen sections and overlaid (Y). Yellow arrows point to *twist2*^+^/Runx2^+^ preosteoblasts. Hoechst-labeled nuclei are in blue. Scale bars represent 50 μm except in H, which represents 5 μm.

We performed lineage tracing to confirm the distal, leading edge Runx2^+^ cells were derived from preexisting osteoblasts. We generated adult zebrafish with caudal fins containing isolated fin rays lined with permanently labeled mCherry^+^ osteoblasts (Stewart and Stankunas, 2012) (Figure 2A). At 72 hpa, fins from the same animal displayed abundant mCherry^+^ osteoblasts in the regenerating tissue (Figure 2B). Immunostaining showed that Runx2^+^ cells extending from labeled hemirays coexpressed mCherry (Figures 2C–2H). Likewise, Runx2^+^ blastema cells observed at 24 or 48 hpa were also derived from mosaic-labeled osteoblasts (Figure S1). These results demonstrate that fin amputation triggers the migration of osteoblasts closely associated with the bone ray into the nascent blastema following their upregulation of Runx2. These Runx2^+^ blastema cells are the precursors to redifferentiated osteoblasts that will line and mineralize replacement bone.

We hypothesized that fin amputation induces an epithelial-to-mesenchymal transformation (EMT) of osteoblasts, which display an underappreciated epithelial-like organization (Ferrari et al., 2000; Izu et al., 2011), to generate Runx2^+^ preosteoblasts. We immunostained nonregenerating fins with zns-5 (Johnson and Weston, 1995), α-catenin, and β-catenin antibodies to label osteoblasts (zns-5) and adherens junctions that interconnect epithelial sheets (catenins). Zns-5-positive osteoblasts expressed both α- (Figures 2I–2L) and β-catenin (Figure S2A), which were concentrated at membranes separating individual osteoblasts, demonstrating that fin ray osteoblasts have epithelial-like properties.

We examined α-catenin localization in 96 hpa fins from transgenic *Tg(sp7:EGFP)b1212* animals, which express EGFP in mature and differentiating osteoblasts (DeLaurier et al., 2010). We saw membrane-localized α-catenin staining in both Runx2^+^/*sp7:EGFP*^+^ and *sp7:EGFP*^+^ cells that were in close proximity to new bone. In contrast, distally located Runx2^+^ cells did not express α-catenin, suggesting they lacked adherens junctions (Figures 2M–2R). N-cadherin, another component of adherens junctions (Lim and Thiery, 2012), also was expressed in maturing osteoblasts (Figures S2B–S2E). At 24 hpa, we observed α-catenin staining only in the fin epidermis (Figures 2S and 2T), indicating that osteoblasts rapidly lose α-catenin expression as they populate the blastema and acquire a progenitor state. Further indicative of EMT, osteoblasts changed cell shape from strikingly long and thin epithelial cells lining nonre-generating bone to a more compact, polygonal morphology when they became Runx2^+^ and Runx2^+^/sp7^+^ blastema-populating cells (compare Figures S3E and S3F).

EMTs are directed by transcription factors including Snail/Slug and Twist (Lim and Thiery, 2012). In developing bone, Twist1 and Twist2 are expressed in Runx2^+^ preosteoblasts where Twist interacts with Runx2 (Bialek et al., 2004; Fulzele et al., 2010; Tran et al., 2010). RNA in situ hybridization showed that both *twist2* and *runx2a* were induced at 24 hpa in tissue immediately adjacent to the amputation site (Figures 2U and 2V). Combining fluorescent in situ hybridization with Runx2 immunostaining on 72 hpa sections succinctly demonstrated that distal Runx2^+^ cells coexpressed *twist2* mRNA (Figures 2W–2Y) and are thus bona fide mesenchymal cells. Similar to *twist2*, *twist3* was expressed at 24 hpa adjacent to the amputated bone (Figure S2G) and by 72 hpa was distally localized in a pattern reminiscent of *twist2* (Figures S2F and S2H). We conclude that Runx2^+^ cells originate from an EMT of epithelial-organized differentiated osteoblasts present near the amputation site, and, throughout regeneration, distal Runx2^+^ preosteoblasts are maintained in a mesenchymal *twist2/3*-expressing state. However, the initial induction of Runx2 expression does not appear to depend on EMT, because epithelial osteoblasts lining the bone proximal to the amputation site also upregulate Runx2^+^ (Figure 1).

### Wnt/β-Catenin Signaling Is Active in Preosteoblasts

**Figure 3:**
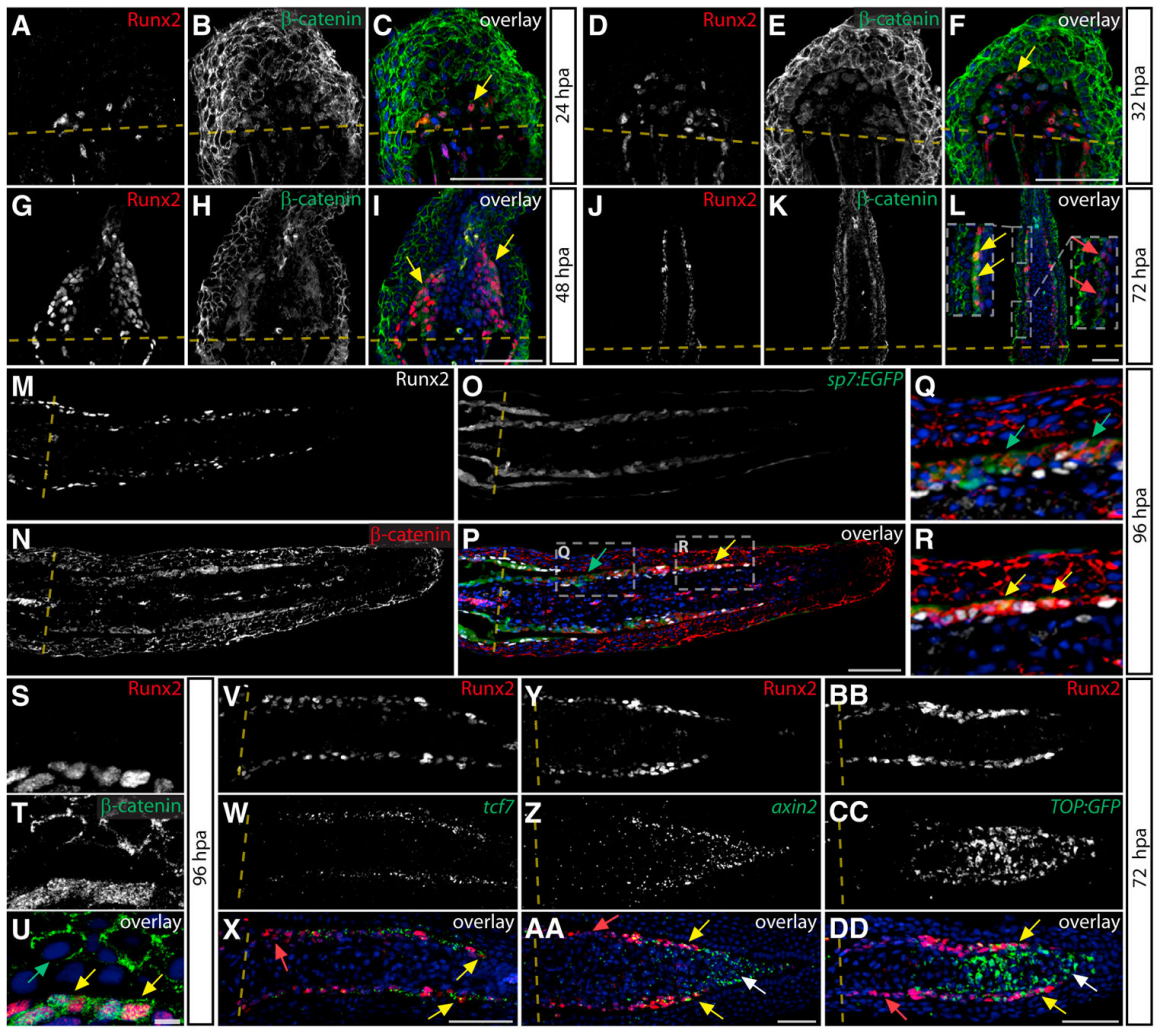
Wnt/β-Catenin Signaling in Preosteoblasts during Regeneration (A–L) Immunostaining of Runx2 (red) and β-catenin (green) on fin sections at 24 (A–C), 32 (D–F), 48 (G–I), and 72 (J–L) hpa. Yellow arrows indicate Runx2^+^ cells with nuclear β-catenin, and the red arrows point to proximal osteoblasts at 72 hpa with membrane-localized β-catenin. For (L), regions bounded by dashed white boxes are shown in higher magnification in inset panels. (M–R) Expression of Runx2 (M, white), *sp7:EGFP* (O, green), and localization of β-catenin (N, red) is shown and overlaid (P–R) on 96 hpa *Tg(sp7:EGFP)* section. Regions bound by dashed white boxes in (P) are shown at higher magnification in (Q) and (R). Yellow arrows highlight Runx2^+^ cells with nuclear-localized β-catenin, and green arrows indicate membrane-localized β-catenin in *sp7:EGFP*^+^ osteoblasts. (S–U) A single optical section at 96 hpa fin showing immunostaining of Runx2 (red) and β-catenin (green) in distal preosteoblasts. Yellow arrows point to Runx2^+^ nuclei containing β-catenin, and the green arrow indicates exclusively membrane-localized β-catenin in the fin epidermis. (V–X) Runx2 immunostaining (V, red) and *tcf7* in situ hybridization (W, green) on a 72 hpa cryosection (X, overlay). The yellow arrow indicates distal Runx2^+^ preosteoblasts that coexpress *tcf7*; the red arrow indicates proximal osteoblasts lacking *tcf7* expression. (Y–AA) Runx2 immunostaining (Y, red) combined with *axin2* in situ hybridization (Z, green) is shown on a 72 hpa cryosection and overlaid (AA). Yellow arrows point to distal preosteoblasts coexpressing *axin2* and Runx2. The white arrow indicates distal nonosteoblast blastema cells that express *axin2*. The red arrow shows proximal osteoblasts without *axin2* expression. (BB–DD) Runx2 immunostaining (BB, red) and *GFP* in situ hybridization (CC, green) on a 72 hpa section from a *Tg(TOP:GFP)* fin shown in overlay (DD). Yellow arrows show Runx2^+^/GFP^+^ preosteoblasts. The white arrow indicates distal nonosteoblast blastema cells that express the reporter. Hoechst-stained nuclei are shown in blue. Scale bars represent 50 μm except in U, which represents 5 μm.

Wnts promote EMT (Stark et al., 1994), osteogenesis (Day et al., 2005; Hill et al., 2005; Hu et al., 2005), and fin regeneration (Poss et al., 2000; Kawakami et al., 2006; Stoick-Cooper et al., 2007). We monitored Wnt signaling in regenerating fins by examining β-catenin localization by antibody staining. At 24 and 32 hpa, we saw Runx2^+^ cells with diffuse nuclear β-catenin localization dispersed in the mesenchyme distal to the amputation site (Figures 3A–3F). Nuclear β-catenin was still apparent at 48 hpa when Runx2^+^ cells became organized along the anterior-posterior body axis (Figures 3G–3I). At 72 hpa, strong nuclear β-catenin staining persisted in distally residing Runx2^+^ preosteoblasts, with less nuclear β-catenin in maturing osteoblasts located closer to the amputation site (Figures 3J–3L), suggesting that downregulation of Wnt signaling accompanies osteoblast differentiation. At 96 hpa, *Tg(sp7:EGFP)* animals showed robust nuclear β-catenin primarily in distal Runx2^+^ cells and not in proximal osteoblasts that expressed only *sp7:EGFP* (Figures 3M–3R and S3A–S3D). High-magnification single optical sections confirmed overlapping nuclear β-catenin and Runx2 in distal osteoblasts at 96 hpa (Figures 3S–3U). Expression of the Wnt transcriptional effector *tcf7* was also enriched in Runx2^+^ osteoblasts (Figures 3V–SX, S3G, and S3H), supporting the potential of Runx2^+^ cells to respond to Wnt signals.

We examined *axin2* expression, a well-accepted target gene of canonical Wnt signaling (Clevers and Nusse, 2012), to monitor Wnt-responsive cell types in the regenerating fin. At 72 hpa, *axin2* was strongly expressed in distal Runx2^+^ preosteoblasts (Figures 3Y–3AA, S3I, and S3J). *Axin2* transcripts additionally were detected in Runx2^−^ cells directly medial to the preosteoblasts and extending to the extreme tip of the blastema. These nonosteoblasts also showed low levels of nuclear β-catenin (Figures S3A–S3D). We further examined Wnt activity using *Tg(TOP:GFP)w25* transgenic fish that express destabilized GFP under the control of a synthetic Wnt-responsive promoter (Dorsky et al., 2002). Due to the rapid turnover of the destabilized GFP, we monitored *GFP* expression by in situ hybridization. At 72 hpa, *TOP:GFP* was found in cells of the distal blastema, including Runx2^+^ preosteoblasts, in a pattern indistinguishable from *axin2* (Figures 3BB–3DD). We did not observe nuclear β-catenin, *axin2* or *TOP:GFP* expression in the epidermis, suggesting that Wnt/β-catenin is not a major regulator of fin epidermis regeneration even though the basal epidermis expresses the Wnt effector *lef1* (Poss et al., 2000). Collectively, the striking nuclear β-catenin, *axin2* expression, and *TOP:GFP* activity in leading edge Runx2^+^ cells suggest ongoing roles for canonical Wnt signaling in regulating Runx2^+^ preosteoblasts from their earliest EMT-driven emergence following amputation through later stages of regenerative outgrowth. Our data further indicate that nonosteoblast lineage cells in the distal blastema represent an additional node of Wnt/β-catenin signaling.

### Wnt Production and Signaling Is Required for Osteoblast EMT

**Figure 4:**
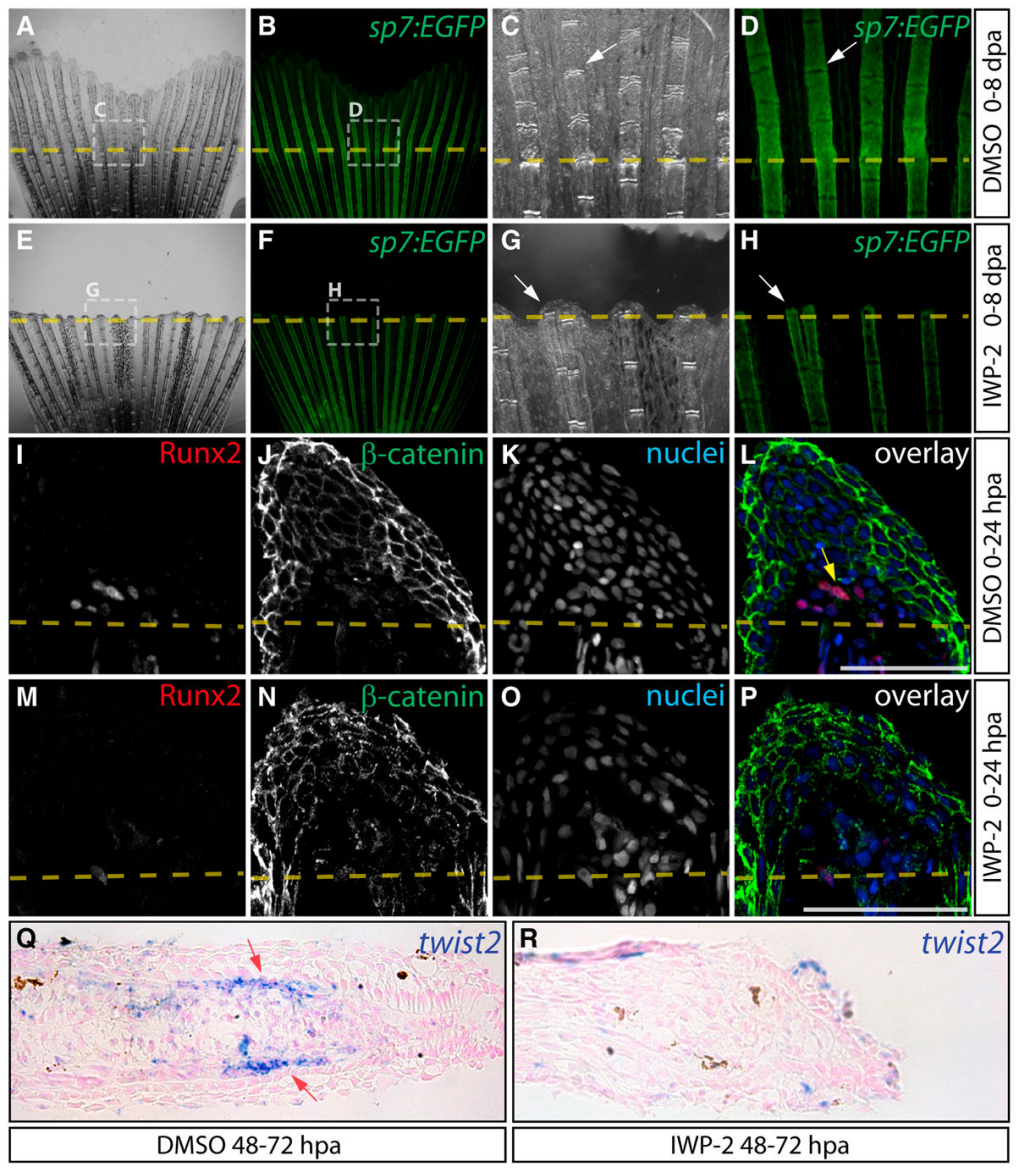
Wnt/β-Catenin Is Required for Osteoblast EMT and Dedifferentiation (A–H) Regeneration of *Tg(sp7:EGFP)* fins after treatment with DMSO (A–D) or IWP-2 (E–H, 10 μM from 0 to 8 dpa). Rotterman contrast (A, C, E, G) and epi-fluorescence (B, D, F, H) images show *sp7:EGFP* expression in osteoblasts (white arrows) before amputation (A, B, E, F) and at 8 dpa (C, D, G, H). Shown are 25× images from one of three fish for control and IWP-2 groups and regions within dashed white boxes are shown at 120× magnification. (I–P) Immunostaining for Runx2 (red) and β-catenin (green) on 24 hpa sections from fish exposed to DMSO (I–L) or Wnt inhibitor (M–P, 10 μM IWP-2 from 0 to 24 hpa). The yellow arrow indicates Runx2^+^ cells with nuclear-localized β-catenin. Nuclei are stained blue. Scale bars represent 50 μm. (Q and R) *twist2* in situ hybridization on fins from DMSO (Q) and IWP-2-treated (R, 10 μM from 48 to 72 hpa) fish harvested 72 hpa. The red arrows point to *twist2*-expressing osteoblasts.

Wnt is one secreted factor that can initiate EMT during embryonic development (Lim and Thiery, 2012; Stark et al., 1994), and Wnts induce *twist* expression during mouse craniofacial bone development (Tran et al., 2010). We performed loss-of-function studies of Wnt signaling during fin regeneration using IWP-2, a small molecule inhibitor of Porcupine, an acyltransferase that covalently modifies Wnts and is required for their secretion (Chen et al., 2009). We treated *Tg(sp7:EGFP)* animals with IWP-2 and monitored regeneration in individual animals from 0 to 8 dpa. At 2 and 4 dpa, IWP-2-treated fish arrested regeneration after forming a wound epidermis and lacked *sp7:EGFP^+^* osteoblasts beyond the amputation site (Figure S4). By 8 dpa, control animals had substantially regenerated and *sp7:EGFP^+^* osteoblasts were seen throughout the regenerate (Figures 4A–4D and S4). In contrast, IWP-2-treated fish displayed no regenerative outgrowth, although bone proximal to the amputation site continuously maintained *sp7:EGFP^+^* expression (Figures 4E–4H and S4). Low doses (100 nM) of Wnt-C59, a chemically distinct Porcupine inhibitor (Proffitt et al., 2013), also completely inhibited bone regeneration (Figures S5A–S5D).

Given the complete lack of bone regeneration in both IWP-2-and Wnt-C59-treated fish, we speculated that an initiating step(s) requires Wnt activity. Sections of fins from fish treated with IWP-2 from 0 to 24 hpa lacked both nuclear-localized β-catenin and Runx2-expressing blastema cells (Figures 4I–4P). However, Runx2 expression was still evident in bone-lining osteoblasts near the amputation site, suggesting its initial activation is Wnt-independent (Figures 4I–4P). A later treatment with IWP-2 from 48 to 72 hpa abolished *twist2* expression (Figures 4Q and 4R). These results demonstrate that Wnt initiates osteoblast EMT and then maintains the mesenchymal state of preosteoblasts by promoting *twist2* expression.

### Wnt/β-Catenin Signaling Maintains Runx2^+^ Preosteoblasts

**Figure 5:**
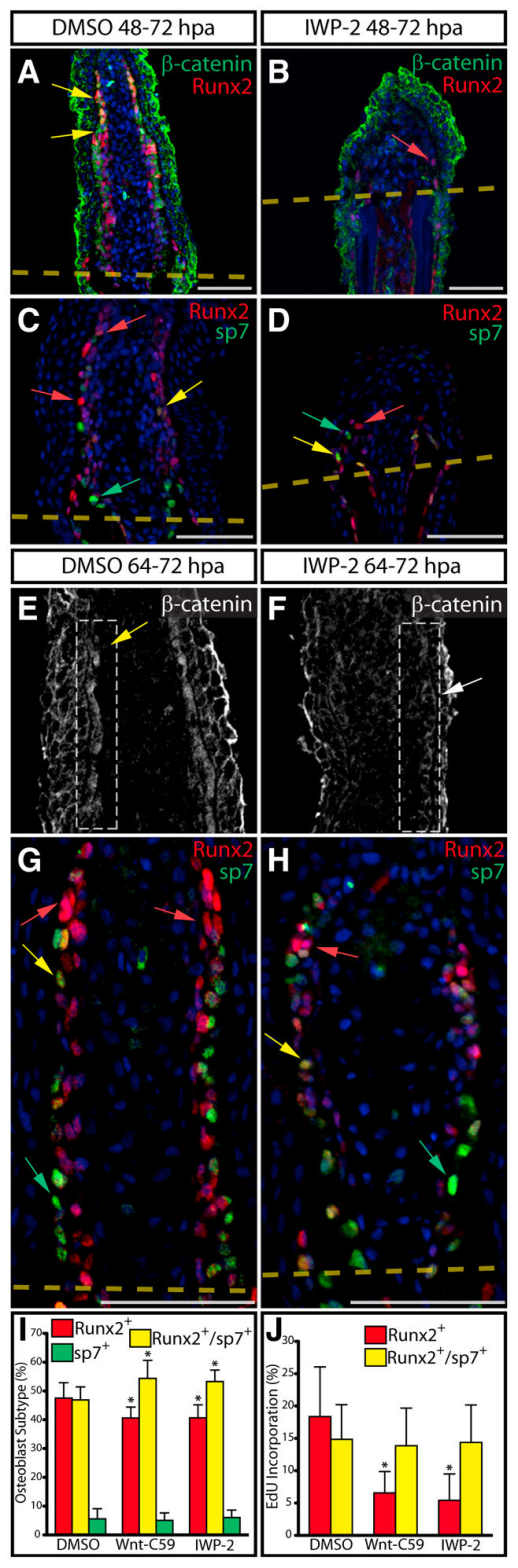
Wnt Is Required for Maintenance of Runx2^+^ Preosteoblasts during Fin Regeneration (A–D) Runx2 (A–D, red), β-catenin (A and B, green), and sp7 (C and D, green) protein expression on sections from DMSO (A and C) and IWP-2-treated (B and D, 10 μM from 48 to 72 hpa) fish. In (A) and (B), yellow arrows indicate Runx2^+^ cells with nuclear-localized β-catenin and the red arrow points to a Runx2^+^ cell lacking nuclear β-catenin. In (C) and (D), red arrows show Runx2^+^ cells, yellow arrows point to Runx2^+^/sp7^+^ cells, and green arrows mark sp7^+^ cells. (E and F) β-catenin localization at 72 hpa in DMSO (E) and IWP-2-treated fish (F, 10 μM at 64–72 hpa). The osteoblast-populated domain of the blastema is bounded by a dashed white box, and the white arrows point to osteoblasts with β-catenin expression. (G and H) Runx2 (red) and sp7 (green) levels in DMSO (G) and IWP-2-treated fins (H, 10 μM at 64–72 hpa) harvested 72 hpa. Red, yellow, and green arrows indicate Runx2^+^, Runx2^+^/sp7^+^, and sp7^+^ cells, respectively. Nuclei are in blue. Scale bars represent 50 μm. (I) Osteoblast subtype percentile representation on matched fin sections (n > 6 rays collected from three animals and representing >600 osteoblasts for each treatment) harvested 72 hpa following DMSO (64–72 hpa), Wnt-C59 (100 nM, 64–72 hpa), or IWP-2 (10 μM, 64–72 hpa) exposure. Asterisks indicate significant differences relative to DMSO-treated fish (all p < 0.02, one-tailed Student’s t tests). (J) Percentage of Runx2^+^ and Runx2^+^/sp7^+^ osteoblasts in the same sections scored in (I) that had incorporated EdU. Asterisks indicate a significant decrease (p < 0.003, one tailed Student’s t tests).

We tested if Wnt has an ongoing role maintaining preosteoblasts during bone regrowth by blocking Wnt production from 48 to 72 hpa using IWP-2. This IWP-2 regimen stunted overall regeneration and depleted osteoblast-lineage cells distal to the amputation site (Figures 5A–5D). The few remaining cells displayed membrane-localized β-catenin and reduced levels of Runx2 (Figures 5A and 5B), whereas sp7 expression was largely unchanged (Figures 5C and 5D). Along with fewer osteoblasts, fins from IWP-2-treated fish had a pronounced decrease in collagen deposition near amputated bones (Figures S5M–S5P). Although the small number of osteoblasts remaining after a 24 hr IWP-2 treatment precluded a detailed analysis, we were able to rule out apoptosis as a cause of the phenotype by TUNEL staining (Figures S5Q–S5V).

At least two populations of distally located cells transmit Wnt signals in the regenerating fin: Runx2^+^ preosteoblasts and adjacent nonosteoblast distal mesenchymal cells. Therefore, the loss of osteoblasts after 24 hr IWP-2 treatment could represent direct and/or indirect Wnt signaling roles. To distinguish between these possibilities, we inhibited Wnt for brief periods (8 hr) during regeneration and monitored β-catenin, Runx2, and sp7 by immunostaining. Administration of IWP-2 or Wnt-C59 from 64 to 72 hpa was sufficient to inhibit nuclear localization of β-catenin (Figures 5E and 5F and S5E–S5L). An examination of osteoblast subtypes (Figures 5G–5J) indicated both IWP-2- and Wnt-C59-treated fish contained fewer Runx2^+^ cells (Figure 5I, p < 0.02) and increased numbers of Runx2^+^/sp7^+^ cells (Figure 5I, p < 0.02). However, no significant difference in the number of sp7^+^ cells was observed (Figure 5I). EdU incorporation in Runx2^+^ cells, but not sp7-expressing osteoblasts, was also reduced in fish treated with either IWP-2 or Wnt-C59 (Figure 5J, p < 0.003), indicating that Wnt-dependent proliferation specifically maintains Runx2^+^ preosteoblasts. We also quantified Runx2 and sp7 expression on sections from multiple animals and produced scatterplots depicting normalized expression levels in individual cells. Confirming our qualitative observations, there was a 3.1-fold decrease in the fraction of Runx2 single positive cells relative to all mesenchymal cells in animals treated with IWP-2 from 64 to 72 hpa (Figures S5W and S5X).

We developed a method to isolate and culture primary fin osteoblasts where 78.5% of the cells stained positively for Runx2 and/or sp7 (SD = 8.3%, n = 5 independent cell preparations). To test if Wnt directly promotes fin osteoblast proliferation, we treated these cultures with Wnt3A and monitored EdU incorporation in Runx2^+^ cells. Wnt3A significantly increased the fraction of EdU^+^/Runx2^+^ cells (Figures S5Y–S5DD, p = 0.0021). We conclude that, in regenerating fins, continuously secreted Wnt activates canonical Wnt signaling in Runx2^+^ preosteoblasts to support, directly or indirectly, their renewal.

### BMP Signaling Promotes sp7 Expression and Osteoblast Differentiation during Bone Regeneration

**Figure 6:**
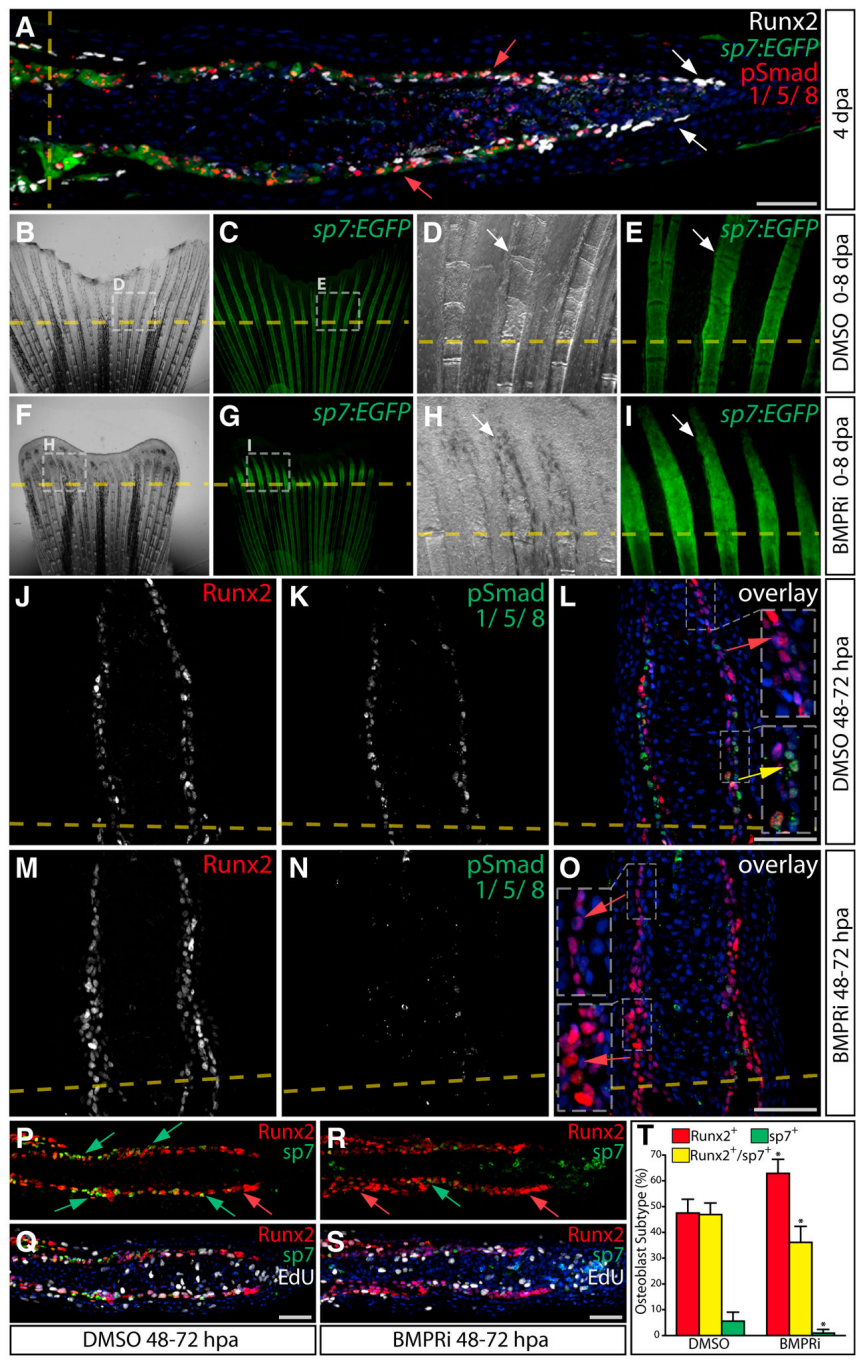
BMP/Smad Signaling Promotes Osteoblast Differentiation and sp7 Expression during Bone Regeneration (A) Runx2 (white), *sp7:EGFP* (green) and pSmad1/5/8 (red) levels on sections from a *Tg(sp7:EGFP)* fish 4 dpa. White arrows point to Runx2^+^/pSmad^−^ preosteoblasts. Red arrows indicate sp7^+^/pSmad1/5/8^+^ osteoblasts. The amputation site is marked with a dashed yellow line. (B–I) Whole-mount images of regenerating fins of *Tg(sp7:EGFP)* fish at 8 dpa after DMSO (B–E) or BMPRi exposure (F–I, 5 μM from 0 to 8 dpa). Rotterman contrast (B, D, F, and H) and epifluorescence images (C, E, G, and I) to visualize *sp7:EGFP* expression (green) are shown. White arrows indicate osteoblasts. Representative images from one of three fish in each of the control and BMPRi groups are shown at low and high magnification. (J–O) Antibody-stained fin sections showing Runx2 (red) and pSmad1/5/8 (green) levels in fish 72 hpa exposed to DMSO (J–L) or BMPRi (M–O, 5 μM BMPRi at 48–72 hpa). Red arrows indicate Runx2^+^/pSmad^−^ cells, and the yellow arrow points to a Runx2^low^/pSmad1/5/8^+^ cell. Insets show magnified boxed regions. (P–S) EdU (white) incorporation in Runx2- (red) and sp7- (green) expressing cells in fin sections from DMSO (P and Q) and BMPRi-exposed animals (R and S, 5 μM at 48–72 hpa). Red arrows indicate Runx2^+^ cells and green arrows point to sp7^+^ cells. Overlay panels show Hoechst-stained nuclei in blue. Scale bars represent 50 μm. (T) The percentage of Runx2^+^ (red bars), Runx2^+^/sp7^+^ (yellow bars), and sp7^+^ cells (green bars) in fin sections from DMSO (48–72 hpa) versus BMPRi-treated (5 μM, 48–72 hpa) zebrafish. Four rays from three BMPRi-exposed animals containing a combined >400 osteoblasts were scored. The DMSO sample data are shared with the experiment shown in Figure 5I. Error bars are one SD, and significant p values are indicated with an asterisk (p < 0.002 for increased Runx2^+^ cells; p < 0.007 for a decrease in Runx2^+^/sp7^+^ cells; and p < 0.02 for a decreased sp7^+^ population, Student’s t tests).

BMPs have been implicated in osteoblast differentiation (Abzhanov et al., 2007; Bandyopadhyay et al., 2006) and are expressed in fin osteoblasts during regeneration (Smith et al., 2006). We examined the pattern of BMP activity by staining fin sections from 96 hpa *Tg(sp7:EGFP)* fish with antibodies against phosphorylated, active Smad1, 5, and 8 (pSmad1/5/8), Runx2, and EGFP. We observed pSmad1/5/8 in differentiating *sp7:EGFP^+^* cells but not Runx2^+^/*sp7:EGFP*^−^ preosteoblasts or other cell types (Figure 6A). pSmad1/5/8 staining was evident by 48 hpa, coincident with the appearance of sp7^+^ maturing osteoblasts (Figures S7A–S7D). The discrete localization of pSmad1/5/8 suggests that differentiating osteoblasts are the primary targets of BMP signals during fin regeneration and that the pathway promotes osteoblast redifferentiation.

We blocked BMP signaling in *Tg(sp7:EGFP)* animals using the BMPR inhibitor (BMPRi) LDN193189 (Cuny et al., 2008) from 0 to 8 dpa (Figures 6B–6I and S6). At 2 dpa, we could not distinguish BMPRi-treated from control fish, but by 4 dpa BMP inhibition produced a pronounced decrease in the extent and levels of *sp7:EGFP* expression (Figure S6). Paradoxically, by 8 dpa of BMPRi treatment, *sp7:EGFP* expression became relatively high, possibly indicating the activation of feedback mechanisms (Figure S6). Regardless, long-term inhibition of the BMP pathway (0–8 dpa) reduced bone formation (Figures 6B–6I) with BMPRi-treated fish lacking mature lepidotrichia including the joints between ray segments but maintaining regeneration of epidermis and blood vessels at 4 dpa (Figures S7E–S7H). Von Kossa staining revealed that fins from BMPRi-treated fish still formed a blastema but failed to produce mineralized bone (Figures S7I–S7L), consistent with a reduced expression domain of *col10a1,* a marker of differentiating osteoblasts (Smith et al., 2006) (Figures S7M and S7N). Importantly, pSmad1/5/8 levels were greatly diminished by chronic BMPRi treatment (Figures S7O and S7P), demonstrating drug efficacy. The bone-specific phenotype induced by BMPRi exposure contrasts those observed with IWP-2 or Wnt-C59, which largely blocked blastema outgrowth, and is consistent with the observation that BMP activity is primarily located in differentiating osteoblasts.

To determine BMP roles during ongoing regeneration, we treated animals with BMPRi from 48 to 72 hpa, which blocked Smad1/5/8 phosphorylation, increased Runx2^+^ cells near the amputation site (Figures 6J–6O), and decreased sp7 expression (Figures 6P and 6R). We scored osteoblast subtypes in DMSO versus BMPRi-treated animals (Figure 6T), confirming BMPRi treatment increased Runx2^+^ cells (p < 0.002) and decreased the Runx2^+^/sp7^+^ and the sp7^+^ populations (p < 0.007 for the Runx2^+^/sp7^+^ cells and p < 0.02 for the sp7^+^ population). EdU incorporation (Figures 6Q and 6S, quantified in Figure S7Q) and TUNEL staining (Figures S5Q–S5V) revealed osteoblast proliferation and cell death, respectively, were unaffected by BMPRi treatment. These results argue against a role for BMP in maintaining Runx2 expression (Smith et al., 2006) and rather demonstrate that BMP specifically drives osteoblast differentiation by promoting the transition from Runx2^+^/sp7^−^ cells to sp7^+^ cells.

### BMP Negatively Modulates Wnt/β-Catenin Activity in Regenerating Osteoblasts

**Figure 7:**
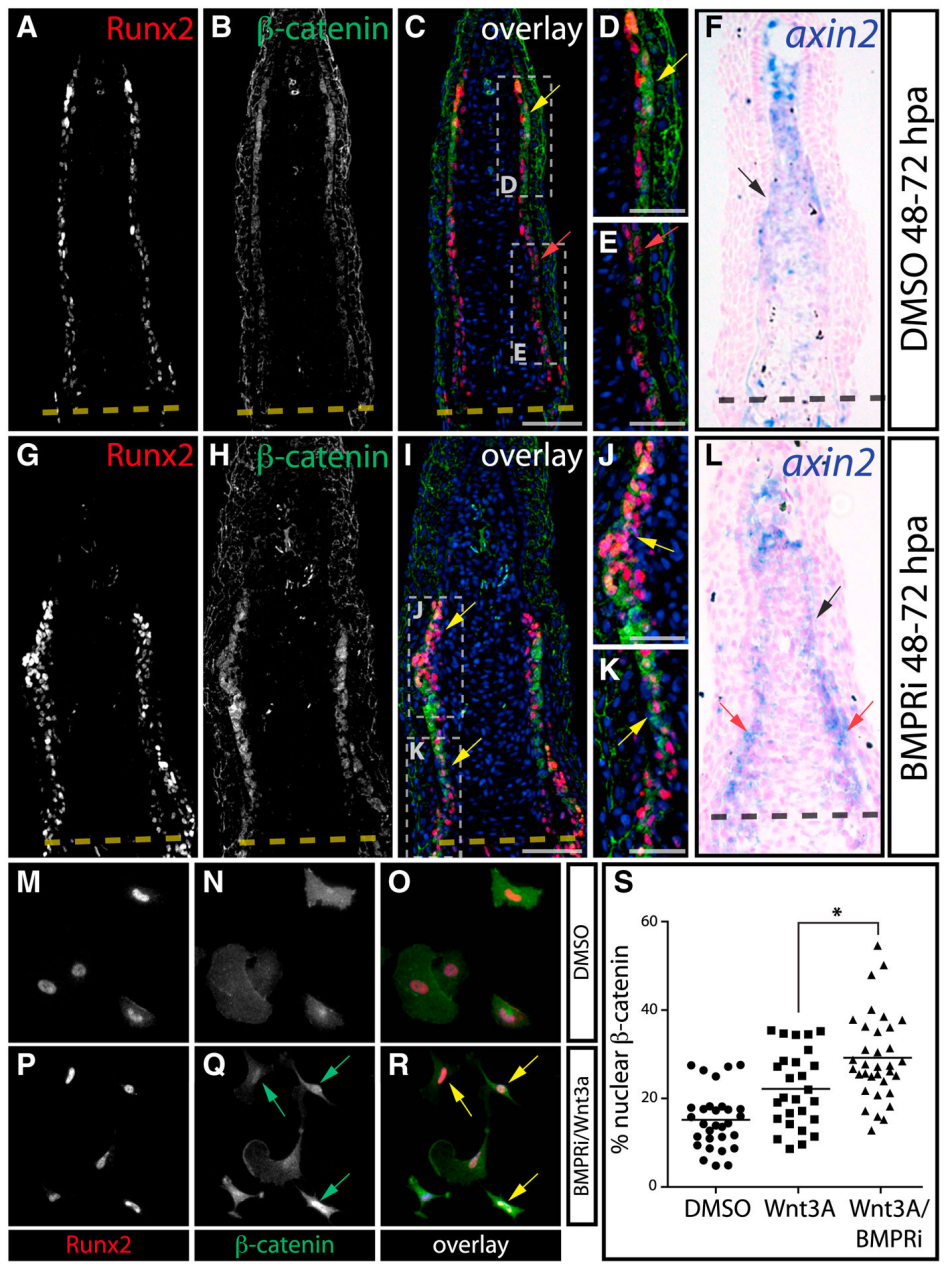
BMP Negatively Regulates Wnt/β-Catenin Signaling (A–L) Immunostaining (A–E and G–K) with Runx2 (red) and β-catenin (green) antibodies and *axin2* in situ hybridization (F and L) on sectioned fins from DMSO (A–F) and BMPRi-treated fish (G–L, 5 μM at 48–72 hpa). Yellow arrows highlight Runx2^+^ cells with nuclear-localized β-catenin, and red arrows point to osteoblasts with membrane-localized β-catenin. Hoechst-stained nuclei are in blue. For (F) and (L), *axin2* expression (blue) in distal osteoblasts is indicated by black arrows. In (L), red arrows point to proximal *axin2*^+^ osteoblasts. Scale bars represent 50 μm (A–C and G–I) and 25 μm (D, E, J, and K). (M–R) Runx2 expression (red) and β-catenin localization (green) in antibody-stained primary zebrafish fin osteoblasts grown in media containing DMSO (M–O) or BMPRi + Wnt3a (P–R, 300 nM and 40 ng/ml, respectively) for 24 hr beginning at 4 days postisolation. Green arrows point to cells with nuclear β-catenin localization, and yellow arrows indicate Runx2^+^ cells displaying nuclear β-catenin. (S) Plots showing nuclear-localized β-catenin versus total cell β-catenin in Runx2^+^ cells in individual, randomly selected cultured fin osteoblasts following DMSO, Wnt3 (40 ng/ml), or BMPRi + Wnt3a (300 nM and 40 ng/ml, respectively) exposure. A line marks the mean of each group. The Wnt3a + BMPRi population displayed significantly increased nuclear β-catenin relative to the Wnt3A-alone-treated group as determined by one-way ANOVA and a post hoc Tukey’s test (p > 0.01, indicated by an asterisk).

Because inhibiting BMP activity expanded the Runx2^+^ population, we hypothesized that BMP signaling in proximally located osteoblasts quenches Wnt/β-catenin activity. Indeed, stained fin sections from 48 to 72 hpa BMPRi-treated fish exhibited a dramatic increase of nuclear-localized β-catenin throughout an expanded population of Runx2^+^ preosteoblasts (Figures 7A–7E and 7G–7K). The domain of *axin2* expression also was expanded proximally in BMPRi-exposed fish, further indicating that BMP functions in proximal osteoblasts to restrain Wnt activity to the distal-most progenitor pool (Figures 7F and 7L).

Cultured primary caudal fin osteoblasts became uniformly pSmad1/5/8^+^ without the addition of exogenous BMP, suggesting cell autonomous BMP production and signaling (Figures S7R–S7U). Among BMPs reported to be expressed in the regenerating fin (Smith et al., 2006), we detected robust expression of *bmp2b* in cultured osteoblasts by quantitative RT-PCR (qRT-PCR) (Figure S7V). We reconstituted BMP’s negative regulation of Wnt/β-catenin by combined addition of BMPRi and recombinant Wnt3A to primary fin osteoblasts. Wnt3A induced partial nuclear accumulation of β-catenin (Figures 7M–7S), which was substantially enhanced by inhibiting BMP signaling using BMPRi (Figures 7P–7S, p < 0.01). These results are congruent with our in vivo observations that BMP signaling is active in osteoblasts where its roles include negatively regulating Runx2^+^ preosteoblasts by countering Wnt signals.

**Figure 8:**
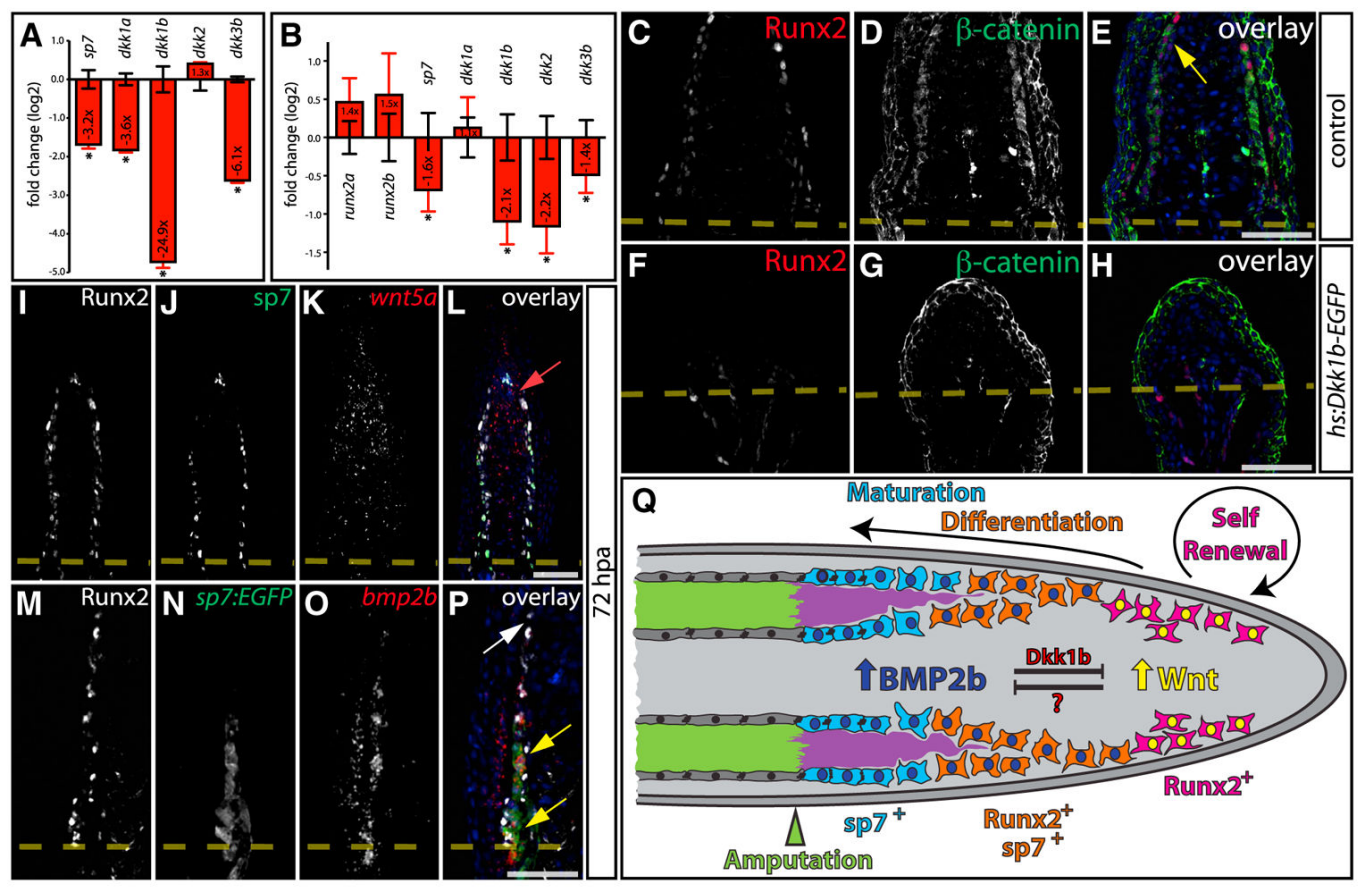
Spatially Distributed Gene Expression Combined with Negative Feedback Maintains Regenerative Osteogenesis (A) Quantitative RT-PCR analysis of *sp7* and *dkk* genes in primary fin osteoblasts after 24 hr of 300 nM BMPRi (red bars and error bars) relative to control DMSO-treated cells (variation shown with black error bars). For each primer pair, the normalized average fold change in transcript levels upon BMPRi treatment is plotted on a log_2_ scale with DMSO samples averaged to log_2_0 = 1. Each group includes three independent cultures. Error bars are one SD. Statistically significant differences are indicated with an asterisk (p < 0.05, two-tailed Student’s t tests). A representative example of three independent experiments is shown. (B) Relative gene expression of osteogenic factors and *dkk* genes at 96 hpa following DMSO (black error bars) or BMPRi exposure (red bars, 5 μM from 48 to 96 hpa). Mean normalized levels of the indicated transcripts from four fins per treatment group are shown on a log_2_ scale. Error bars represent one SD, and asterisks mark differentially expressed genes (p < 0.05, two-tailed Student’s t tests). (C–H) Antibody staining for Runx2 (red) and β-catenin (green) on sectioned fins from control (C–E, two heat treatments between 48 and 72 hpa) and *Tg(hsp70l:dkk1b-GFP)* fish (F–H, two heat treatments between 48 and 72 hpa) harvested 72 hpa. The yellow arrow indicates Runx2^+^ cells with nuclear β-catenin. (I–L) Immunostaining of a 72 hpa fin cryosection with Runx2 (I, white) and sp7 (J, green) antibodies and simultaneous *wnt5a* mRNA in situ hybridization (K, red), overlaid in (L). The red arrow shows *wnt5a* expression in distal mesenchymal cells. (M–P) A fin cryosection from a 72 hpa *Tg(sp7:EGFP)* fish showing antibody staining for Runx2 (M, white) and EGFP (N, green) and *bmp2b* mRNA in situ hybridization (O, red), overlaid in (P). The white arrow points to Runx2^+^/*bmp2b*^−^ cells, and yellow arrows indicate overlapping expression of *sp7:EGFP* and *bmp2b*. Hoechst-stained nuclei are in blue in overlay panels. Scale bars represent 50 μm. (Q) A signaling network model for osteogenesis during fin regeneration. Wnt acts distally to maintain a pool of Runx2^+^ osteoblast progenitor cells, whereas Bmp2b-initiated signaling in progenitor-derived cells both promotes sp7-associated differentiation and constrains Wnt activity by inducing Dkk1b.

Dkk proteins compete with Wnt ligands for binding to Fz/Lrp5/6 receptor complexes (Mao et al., 2001). We therefore hypothesized that BMPs could negatively regulate Wnt/β-catenin signaling by activating Dkk expression. Primary fin osteoblasts expressed *dkk1a*, *dkk1b*, *dkk2*, and *dkk3b*, among which *dkk1a*, *dkk1b*, and *dkk3b* expression were reduced upon BMPRi treatment, as was *sp7* (Figure 8A, p < 0.05). We next examined BMP-dependent expression of *dkk* genes in regenerating fins from fish treated with DMSO or BMPRi from 48 to 96 hpa. *Dkk1b*, *dkk2*, and *dkk3b* all were significantly downregulated in BMPRi-treated animals (Figure 8B, p < 0.05). Consistent with antibody staining and the in vitro results, *sp7* transcript levels were also reduced upon BMPRi treatment. *Dkk2* was downregulated by BMPRi in vivo but not in cultured cells, possibly reflecting nonosteoblast expression of *dkk2* in intact fins. Unique among Dkk proteins, Dkk3 is not thought to directly antagonize Wnt/receptor interactions (Mao et al., 2001). Nevertheless, *dkk3b* was enriched in regenerating osteoblasts, and its expression was reduced by a 24 hr BMPRi treatment from 48 to 72 hpa (Figures S8A and S8B).

The BMP-dependent expression of *dkk1b* and its ability to inhibit Wnt signaling in zebrafish (Shinya et al., 2000) and attenuate fin regeneration (Stoick-Cooper et al., 2007) suggested that Dkk1b may be sufficient to inhibit the Wnt-dependent Runx2^+^ preosteoblast pool. We amputated fins from control and *Tg(hsp70l:dkk1b-GFP)w32* fins, subjected them to a heat shock regimen from 48 to 72 hpa, and examined β-catenin localization and Runx2 expression. Dkk1b induction decreased nuclear-localized β-catenin and produced a pronounced deficiency in Runx2^+^ preosteoblasts (Figures 8C–8H), consistent with Dkk1b being a potent BMP-dependent inhibitor of osteoblast progenitor renewal.

The division of regenerating fin osteoblasts into distal Wnt active and proximal BMP active populations suggests the pathway’s respective ligands would be similarly distributed. Guided by earlier studies (Poss et al., 2000; Smith et al., 2006; Stoick-Cooper et al., 2007), we determined the cell specific expression patterns of *bmp2b*, *wnt5a*, *wnt5b*, and *wnt10a* transcripts on 72 hpa fin sections. In situ hybridization revealed that *bmp2b* mRNA was produced by differentiating proximal osteoblasts (Figure S8C). In contrast, expression of *wnt5a* (Figure S8D) and *wnt5b* (Figure S8E) was concentrated toward the distal end of the regenerating fin, in both blastema mesenchymal cells and basal epidermis. Although more weakly expressed, *wnt10a* was also present in distal and lateral regions of the blastema in or adjacent to osteoblasts (Figure S8F). To conclusively identify the cellular sources of *wnt5a* and *bmp2b* relative to osteoblast lineage cells, we performed double immunostaining/in situ hybridization studies. *Wnt5a* expression was expressed in mesenchymal cells occupying the extreme distal blastema bordered laterally by Runx2^+^ preosteoblasts. Minimal *wnt5a* was detected in Runx2^+^ preosteoblasts or blastema cells adjacent to maturing sp7-expressing osteoblasts (Figures 8I–8L). *Bmp2b* was expressed in maturing sp7^+^ osteoblasts, but not Runx2^+^ distal preosteoblasts (Figures 8M–8P). Based on their spatial separation, Wnt5a/5b and Bmp2b are attractive candidate ligands to simultaneously promote the opposing activities of Wnt-dependent preosteoblast maintenance and BMP-dependent osteoblast differentiation, respectively.

## DISCUSSION

Bone regeneration in zebrafish fins requires regulatory mechanisms to (1) generate dedifferentiated progenitors from mature osteoblasts, (2) maintain this preosteoblast population until regeneration is complete, and (3) spatially and temporally restrict redifferentiation to a subset of preosteoblasts that progressively reform lost bone. The latter two processes are opposing activities; a fine balance between progenitor expansion and terminal differentiation must be maintained to sustain bone regeneration over 2 or more weeks. Our studies suggest a model for bone regeneration in the fin (Figure 8Q) whereby a Wnt-dependent EMT of epithelial osteoblasts populates the newly formed blastema with dedifferentiated Runx2^+^ preosteoblasts. Spatial segregation of two opposing pathways, Wnt/β-catenin and BMP, then balances growth and differentiation until regeneration is complete. Sustained high levels of Wnt in the distal blastema directly and continuously maintain a leading edge pool of Runx2^+^ mesenchymal preosteoblasts. Conversely, as more proximally located Runx2^+^ cells become physically distanced from the distal Wnt source, they upregulate *bmp2b* and activate autocrine BMP signaling. BMP promotes osteoblast differentiation by inducing *sp7* and *dkk1b* expression to feedback inhibit Wnt activity and prevent unproductive overexpansion of the progenitor pool. In agreement, *dkk1b* promoter elements drive gene expression exclusively in differentiating osteoblasts near the amputation site (Kang et al., 2013). As osteoblast maturation ensues, sp7-expressing cells downregulate Runx2, re-epithelialize by reforming adherens junctions, and progressively extend new mineralized bone (Figure 8Q).

### Specific Roles for Wnt and BMP in the Osteoblast Lineage during Regeneration

Our examination of canonical Wnt activity shows that osteoblasts are a major Wnt-responsive cell type in the regenerating fin. Rapid changes in preosteoblast subtypes and their proliferation with short-term Wnt inhibitor exposure and the sufficiency of recombinant Wnt to promote osteoblast proliferation demonstrate canonical Wnt directly affects preosteoblasts. The requirement for Wnt in Runx2^+^ preosteoblast proliferation during fin regeneration is consistent with known functions for the Wnt/β-catenin pathway in maintaining progenitor cells in diverse biological settings (Clevers and Nusse, 2012) and regulating bone development in mice (Day et al., 2005; Glass et al., 2005; Gong et al., 2001; Hill et al., 2005; Hu et al., 2005; Rodda and McMahon, 2006). Precisely how Wnt/β-catenin signaling supports the preosteoblast state requires further study, but it is likely that Wnt/β-catenin’s effects on transcription favor the silencing of genes required for differentiation and activation of genes associated with “stemness” (Clevers and Nusse, 2012).

We also observed *axin2* and *TOP:GFP* expression, as well as modest nuclear β-catenin in nonosteoblast distal blastema cells, indicating they, like preosteoblasts, are Wnt responsive. A role for Wnt signaling outside of osteoblasts is supported by our long-term Wnt inhibitor and Dkk1b overexpression experiments that produce a near complete arrest of fin regeneration. Therefore, akin to its role in preosteoblasts, canonical Wnt signaling may maintain these distal-most blastema cells, which then act as a signaling niche/center that orchestrates the overall fin regeneration process. This concept is described in detail in the accompanying paper from Wehner et al. (2014) in this issue of *Cell Reports*. W*nt5a*, *wnt5b*, and *wnt10a* are expressed in distal regenerating fin tissue (Poss et al., 2000; Stoick-Cooper et al., 2007), results we confirmed and expanded upon by demonstrating that *wnt5a* is expressed in distal blastema cells adjacent to Runx2^+^ preosteoblasts. Therefore, distal blastema cells may be a self-sustaining source of a Wnt morphogen gradient that both directly and indirectly regulates osteoblasts and other cell types of the regenerating fin.

Our observations show that BMP signaling likely is dedicated to differentiating osteoblasts in regenerating fins. Antibody staining localizes pSmad1/5/8 to proximal maturing osteoblasts and not distal Runx2^+^ preosteoblasts. Further, BMP receptor function is required for normal sp7 expression and bone formation, but not to maintain distal Runx2^+^ cells. Intriguingly, although distal Runx2^+^ cells with robust nuclear β-catenin reside near a field of *bmp* expression (Smith et al., 2006), they and other distal cells are pSmad1/5/8 negative and therefore likely refractory to BMP signals. One possible explanation is feedback inhibition of BMP in the distal blastema by Wnt-induced BMP antagonists. Ectopic expression of Chordin, a secreted BMP inhibitor, blocks fin regeneration (Smith et al., 2006), although it is unknown whether endogenous Chordin, or other BMP inhibitors, quench BMP signals in the context of fin regeneration. We find that cultured fin osteoblasts strongly express *bmp2b* and accordingly become pSmad1/5/8^+^. Further, *bmp2b* expression is concentrated in differentiating sp7^+^ osteoblasts in vivo, as suggested by an earlier study (Smith et al., 2006). Direct downstream targets of BMP/Smad during bone regeneration could include both *sp7* and *dkk* genes, such as *dkk1b*. Our model does not resolve if other signaling pathways required for fin ray regeneration, including fibroblast growth factor (FGF) and Hedgehog (reviewed in Gemberling et al., 2013), interface with Wnt/β-catenin and BMPs in osteoblasts or have secondary effects on regenerating bone.

### Opposing Activities of Wnt and BMP Establish Self-Renewing Tissues and Organs

Self-renewing tissues, including intestinal epithelium, stomach lining, interfollicular epidermis, sebaceous glands, and hair follicles of the skin, contain stem cells that produce cells whose differentiation states are both temporally and spatially arranged (Barker et al., 2010). The logic of how these hierarchical progenitor cell systems are maintained is remarkably similar to what we describe during fin bone regeneration with Wnt/BMP mutual antagonism being a common theme (Kandyba et al., 2013; Plikus et al., 2008). Mathematical models predict an obligatory role for negative feedback loops to balance stem cell renewal and differentiation (Lander et al., 2009). Therefore, an equilibrium between Wnt and BMP output may be a common logic component of regulatory networks that establish a precise balance between cell plasticity and differentiation.

### Implications for Restorative Bone Therapies

Our interpretation of bone regenration in the zebrafish fin suggests that the signaling networks that control regenerative osteogenesis are evolutionarily conserved, relatively simple, and largely lineage intrinsic. Human bone may retain the competency to robustly regenerate if inherent networks could be somehow activated and/or augmented. Currently, recombinant BMPs are the only biologic factors approved for use in bone repair therapies; however, whereas BMPs can promote ossification, their clinical efficacy is controversial (Garrison et al., 2010). A potential explanation for this can be inferred from our results and those of others that show that BMPs downregulate Wnt/β-catenin signaling in bone (Minear et al., 2010a). New therapeutic approaches can be envisioned that exploit the features of regenerative osteogenesis that we have uncovered. For instance, first enhancing Wnt/β-catenin activity using small molecules or Wnt proteins soon after injury may expand endogenous preosteoblasts, an idea supported by enhanced healing of damaged bone in mice treated with recombinant Wnts (Minear et al., 2010b). Then, later stages of bone healing may benefit from BMP treatment to enhance osteoblast redifferentiation and mineralization. More broadly, our observations encourage approaches aimed at coaxing human cells to mimic those of animals, like zebrafish, that have remarkably robust regenerative abilities.

## EXPERIMENTAL PROCEDURES

### Zebrafish

Wild-type AB, *Tg(sp7:EGFP)b1212* (DeLaurier et al., 2010), *Tg(hsp70l:dkk1b-GFP)w32* (Stoick-Cooper et al., 2007), *Tg(dusp6:Cre-ERT2,myl7:ECFP*) *b1230* (Stewart and Stankunas, 2012), *Tg(TOP:GFP)w25* (Dorsky et al., 2002), and *Tg(Xla.Eef1a1-actb2:LOXP-LOX5171-FRT-F3-EGFP,mCherry) vu295a* (Boniface et al., 2009) lines were maintained according to University of Oregon institutional guidelines. Regeneration studies were performed at 28°C –29°C as described previously (Stewart and Stankunas, 2012).

### Immunostaining

Sections were rehydrated and antigen retrieval was performed for 10 min in a pressure cooker in retrieval buffer (1 mM EDTA [pH 8], 0.1% Tween 20). Antibodies were diluted in PBST containing 10% nonfat dry milk and applied to slides overnight at 4°C, followed by staining with Alexa-conjugated secondary antibodies (Invitrogen) and Hoechst nuclear staining. Antibody staining details are provided in the Supplemental Experimental Procedures.

### Drug Treatments

Wnt pathway inhibitor IWP-2 and the BMPR inhibitor, LDN193189, referred to in the text and figures as BMPRi, were purchased from StemRD; Wnt-C59 was from Biovision; all were dissolved in DMSO. At the indicated times after fin amputation, IWP-2, Wnt-C59, BMPRi, or DMSO was added to fish water (one animal per 200 ml of water) at 10 μM, 100 nM, or 5 μM, respectively. For each drug treatment (n = 3), cohorts of three or four animals were used, each was analyzed at the completion of the study, and images shown are representative examples of each cohort.

### In Situ Hybridization

For combination fluorescent in situ hybridization immunostaining, sections on slides were sequentially antigen retrieval treated, hybridized with DIG-labeled probes, stained with α-DIG peroxidase-conjugated antibody (Roche), developed using the TSA system (PerkinElmer), and finally immunostained. Protocol details and probe synthesis are described in the Supplemental Experimental Procedures.

### Heat Shock Studies, EdU Labeling, Osteoblast Cell Culture, Quantitative RT-PCR, Mosaic Analysis, TUNEL Staining, and Histology

These methods are presented in the Supplemental Experimental Procedures.

### Statistical Analysis

Statistically significant differences between osteoblast subtypes were determined by scoring their fractional representation on comparable immuno-stained sections (for Figures 1 and 5, >600 osteoblasts from more than six rays compiled from at least three fish; for Figure 6, >400 osteoblasts from more than four rays collected from at least three animals). One-tailed Student’s t tests compared the means of each population’s percentage of total osteoblasts across individual rays. Fisher’s exact tests were used to determine significant differences in the proportion of EdU^+^ cells between osteoblast subtypes, combining osteoblasts scored in 12 rays from at least four fish (>1,100 cells total). To assess changes in EdU incorporation between drug-treated fish, comparable stained sections were scored for the fraction of EdU incorporating cells of each osteoblast subtype. Student’s t tests assessed differences between drug-treated groups using the means of each subtype’s percentage of EdU^+^ incorporating cells across individual rays. For nuclear β-catenin quantitation in cultured osteoblasts, one-way ANOVA and post hoc Tukey’s tests were used to determine significant differences in the ratio of nuclear to total β-catenin in individual cells across and between treatments. To determine differentially expressed gene in qRT-PCR studies, two-tailed Student’s t tests used raw ΔCts from three or four independent control and experimental samples.

## Supplementary Material

1

2
