# Supplementary material for: Sequential and Opposing Activities of Wnt and BMP Coordinate Zebrafish Bone Regeneration
Source: Cell Rep. Author manuscript; Available in PMC 2015 Feb 13. (PMC4009375; doi:10.1016/j.celrep.2014.01.010)
Supplement: 1 [file NIHMS558474-supplement-1.pdf]

SUPPLEMENTAL INFORMATION

Sequential and opposing activities of Wnt and BMP coordinate zebrafish bone regeneration

Scott Stewart, Alan W. Gomez, Benjamin E. Armstrong, Astra Henner, Kryn Stankunas

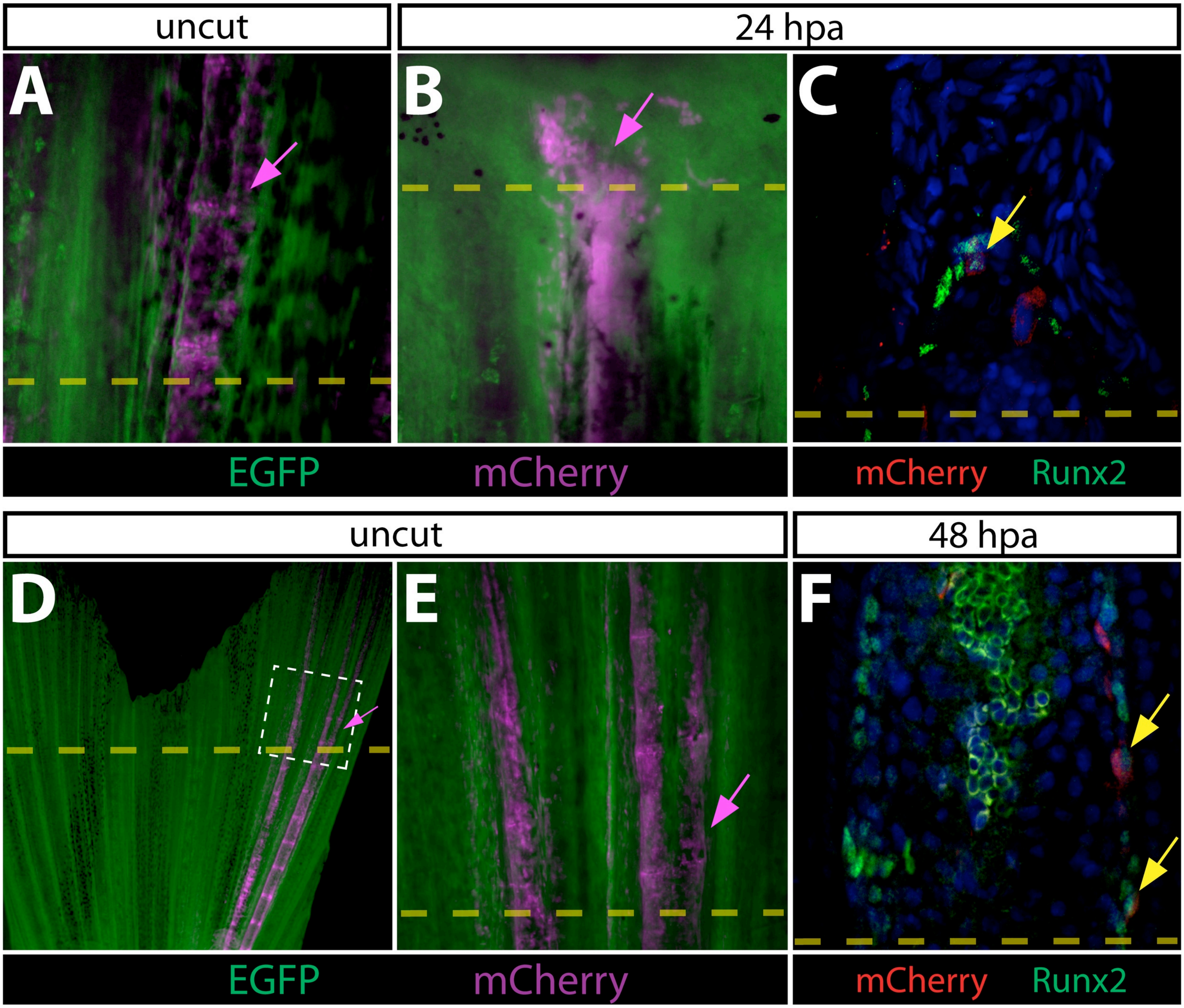

Figure S1, related to Figure 1

Figure S1, related to Figure 1. Pre-existing osteoblasts generate Runx2$^+$ blastema cells.

(A-C) Whole-mount epifluorescence microscopy of a mosaic adult *Tg(Xla.ef1a1-actb2:LOXP-LOX5171-FRT-F3-EGFP,mCherry); Tg(dusp6:CreERT2,myl7:ECFP)* caudal fin mosaic displaying osteoblasts labeled by permanent mCherry expression (magenta) before amputation (A) and at 24 hpa (B). (C) Immunostaining of a section of this fin ray at 24 hpa showing Runx2 (green) and mCherry expression (red) in cells distal to the amputation site (dashed yellow line). Nuclei stained with Hoechst are shown in blue. **(D-F)** Another *Tg(Xla.ef1a1-actb2:LOXP-LOX5171-FRT-F3-EGFP,mCherry); Tg(dusp6:CreERT2,myl7:ECFP)* osteoblast mosaic visualized by whole-mount epifluorescence microscopy at 25x (D). The region in the dashed box is shown at 120x magnification (E). (F) Immunostaining of a section of this mosaic fin at 48 hpa showing Runx2 (green) and mCherry (red) co-expression. Nuclei are stained with Hoechst (blue). The dashed yellow line marks the amputation plane. Magenta arrows point to the osteoblasts expressing mCherry in mosaic lepidotrichia and yellow arrows indicate the Runx2$^+$/mCherry$^+$ mosaic-derived cells.

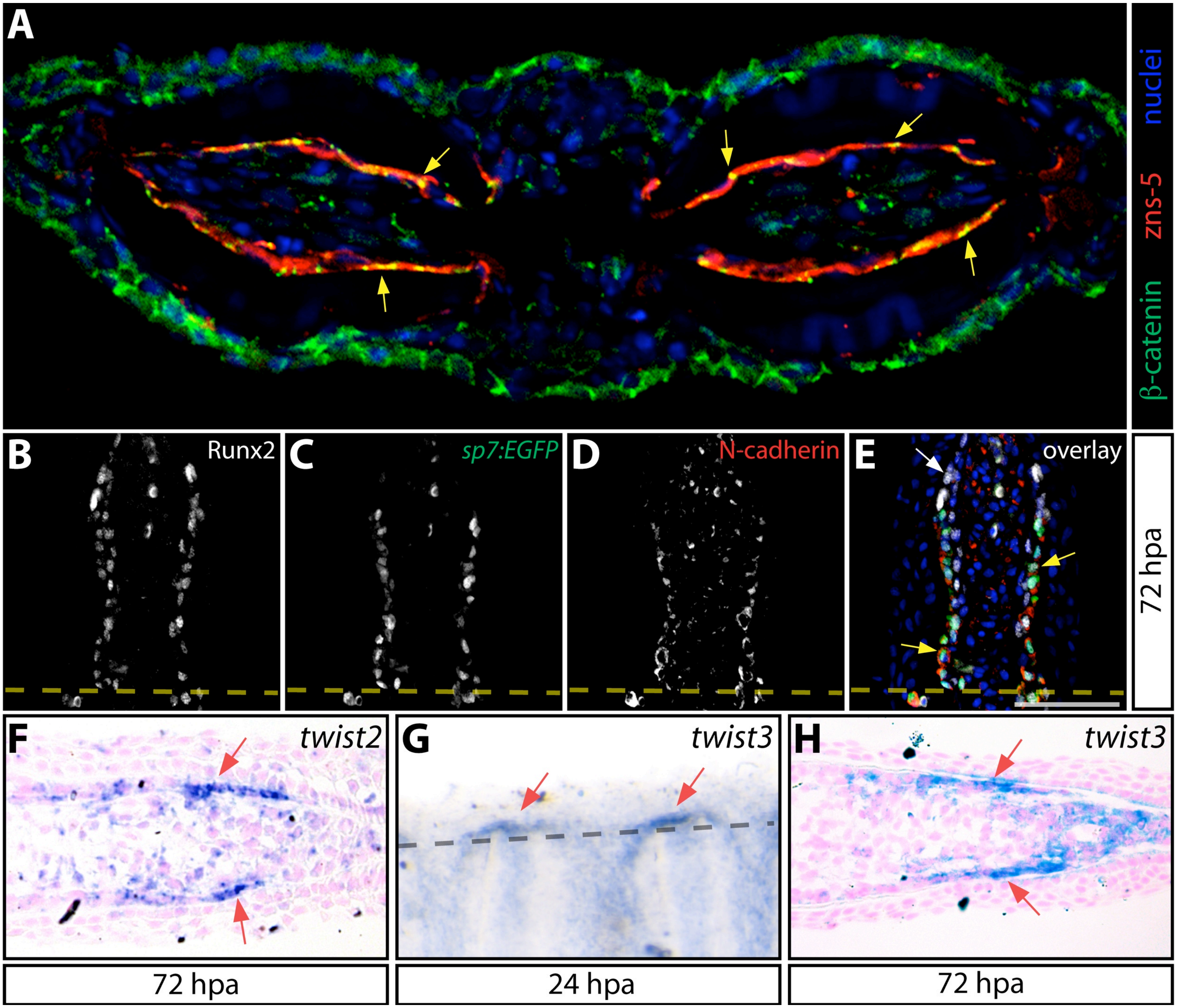

Figure S2, related to Figure 2

Figure S2, related to Figure 2. Dual epithelial/mesenchymal nature of osteoblasts during fin regeneration.

(A) Immunostaining for the osteoblast lineage marker zns-5 (red) and β-catenin (green) on a transverse section from a non-regenerating fin. Hoechst-stained nuclei are shown in blue. Yellow arrows point to adherens junctions between β-catenin/zns-5 labeled osteoblasts. **(B-E)** Expression of Runx2 (B, in white), *sp7:EGFP* (C, in green), and N-cadherin (D, in red) are shown in overlay (E) on 72 hpa fin sections. The white arrow points to distal Runx2^{+} pre-osteoblasts lacking N-cadherin expression and yellow arrows indicate sp7^{+} maturing osteoblasts that display membrane-localized N-cadherin. The yellow dashed line indicates the amputation plane and the scale bar represents 50 μm. **(F)** Expression of *twist2* mRNA (in blue) using in situ hybridization on 72 hpa fin paraffin sections. Red arrows point to distal pre-osteoblasts expressing *twist2*. **(G and H)** In situ hybridization to localize expression of *twist3* on whole-mount fins at 24 hpa. (G) and paraffin sections of 72 hpa fins (H). In (G), red arrows point to cells expressing *twist3* (in blue) distal to the amputation site (grey dashed arrow). In (H), red arrows point to distal pre-osteoblasts exhibiting *twist3* expression (in blue). Shown are representative images from experiments repeated > 3 times.

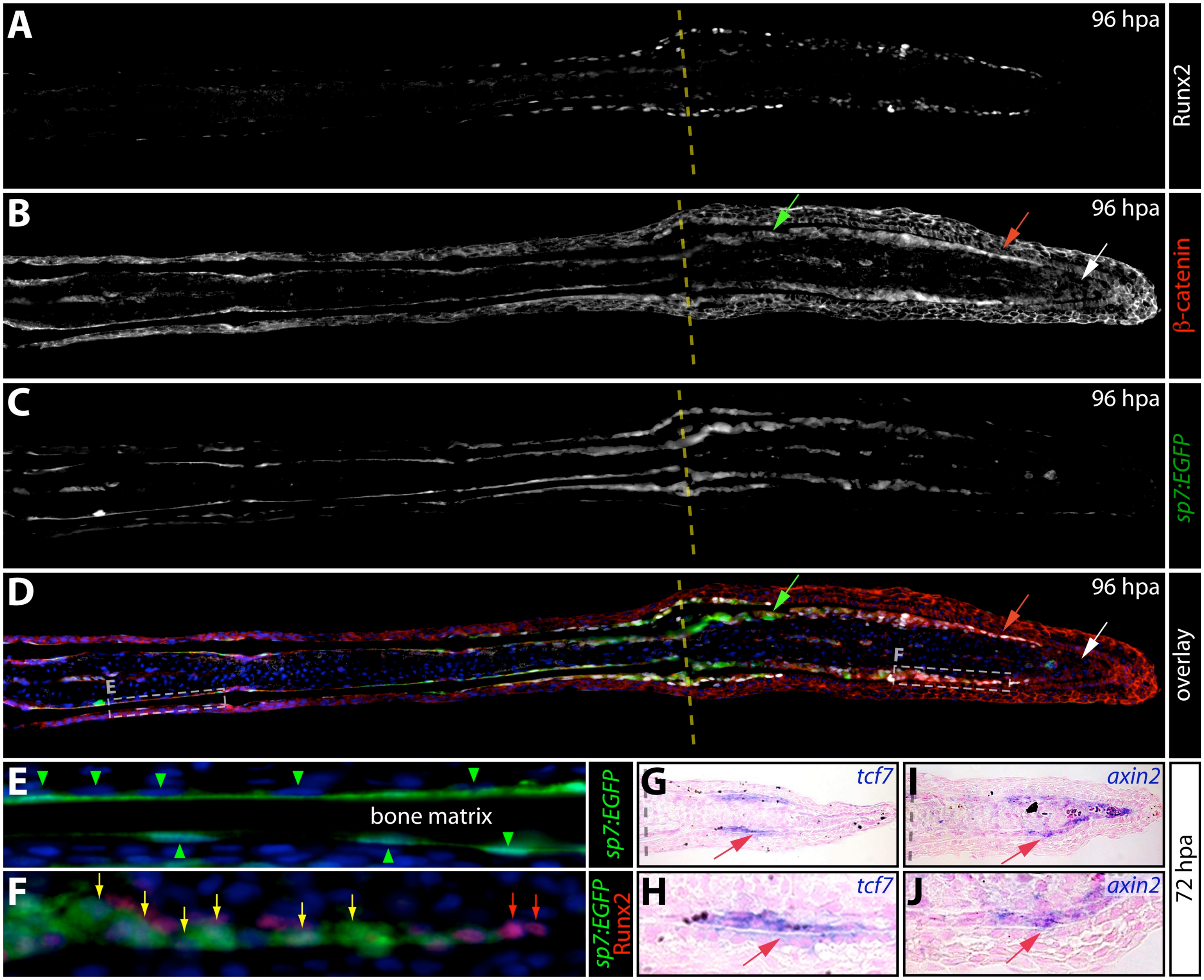

Figure S3, related to Figure 3

Figure S3, related to Figure 3. β-catenin localization in the osteoblast lineage during fin regeneration.

(A-F) Stitched high-resolution widefield epifluorescence images of a fin section from a 4 dpa *Tg(sp7:EGFP)* fish immunostained to show Runx2 expression (A, white), β-catenin localization (B, red), and *sp7:EGFP* reporter activity (C, green). The overlay is shown in (D). Red arrows point to $Runx2^{+}$ cells containing robust nuclear β-catenin, green arrows indicate *sp7:EGFP*$^{+}$ with membrane associated β-catenin, and white arrows point to distal blastema cells with low nuclear β-catenin. The dashed yellow lines show the amputation site. (E, F) High magnification images of the regions bounded by the dashed white boxes in (D) to highlight cell shape in non-regenerating (E) vs. regenerating (F) osteoblasts. Green arrowheads indicate elongated non-regenerating *sp7:EGFP*$^{+}$ epithelial osteoblasts. Red and yellow arrows indicate regenerating distal $Runx2^{+}$ and $Runx2^{+}$/*sp7:EGFP*$^{+}$ cells, respectively, with distinctly rounded morphology. **(G-J)** Detection of *tcf7* (G and H) and *axin2* (I and J) expression by in situ hybridization on sections from 72 hpa fins at low (G and I) and high (H and J) magnification. Red arrows point to specific expression of *axin2* and *tcf7* in distal pre-osteoblasts. The grey dashed line indicates the site of amputation. Shown are representative images indicative of experiments repeated > 6 times.

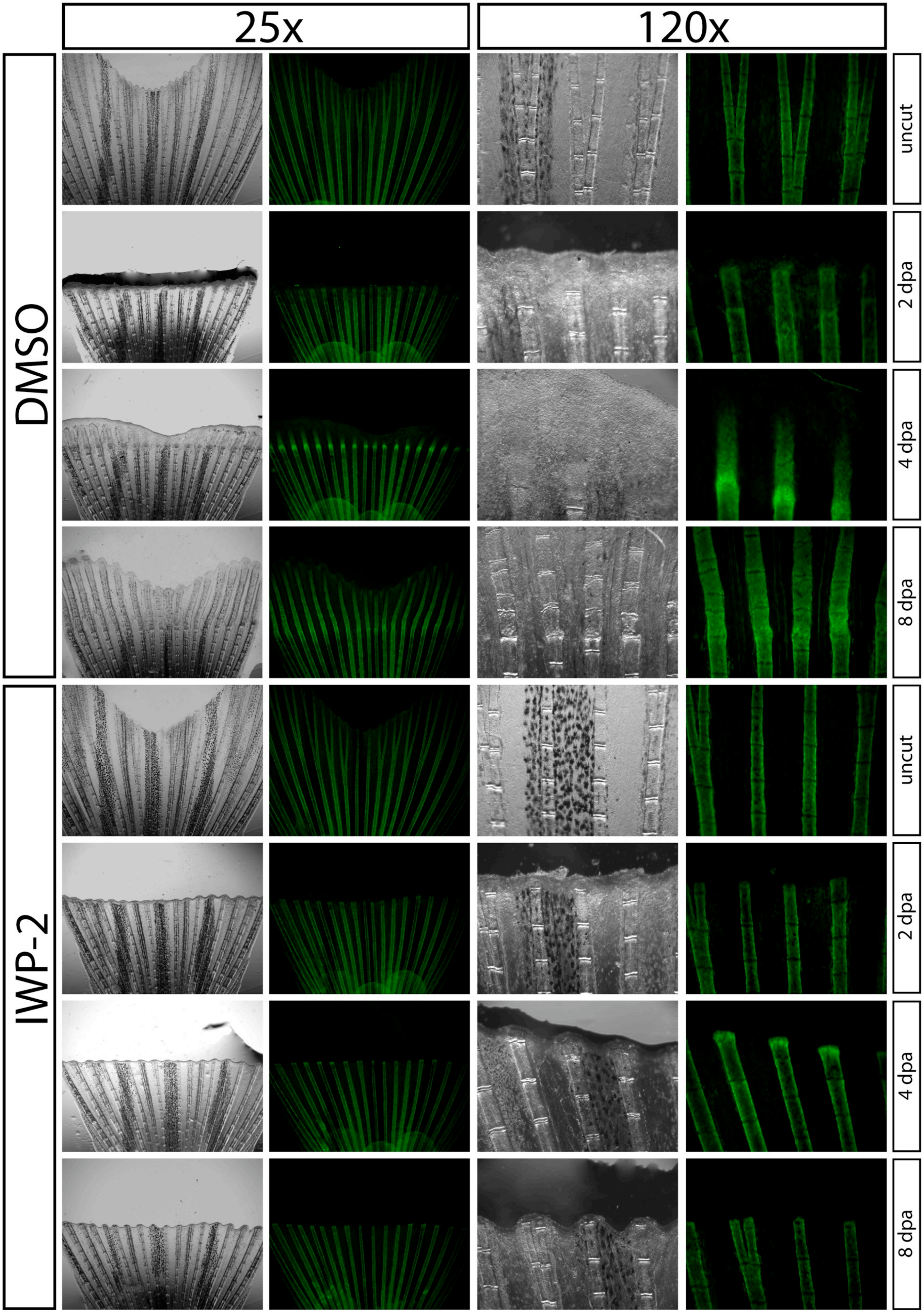

Figure S4, related to Figure 4

Figure S4, related to Figure 4. Wnt production is required for fin regeneration.

Regeneration of caudal fins from *Tg(sp7:EGFP)* fish exposed to DMSO (upper panels) or 10 μM IWP-2 (lower panels) from 0-8 dpa. Each animal is shown before amputation and at 2, 4, and 8 dpa by Rotterman contrast and epifluorescence to visualize *sp7:EGFP* expression (green) in osteoblasts at 25x and at 120x magnification. Shown is one of three fish from each treatment group, within which phenotypes were indistinguishable. The experiment was repeated with three independent fish cohorts.

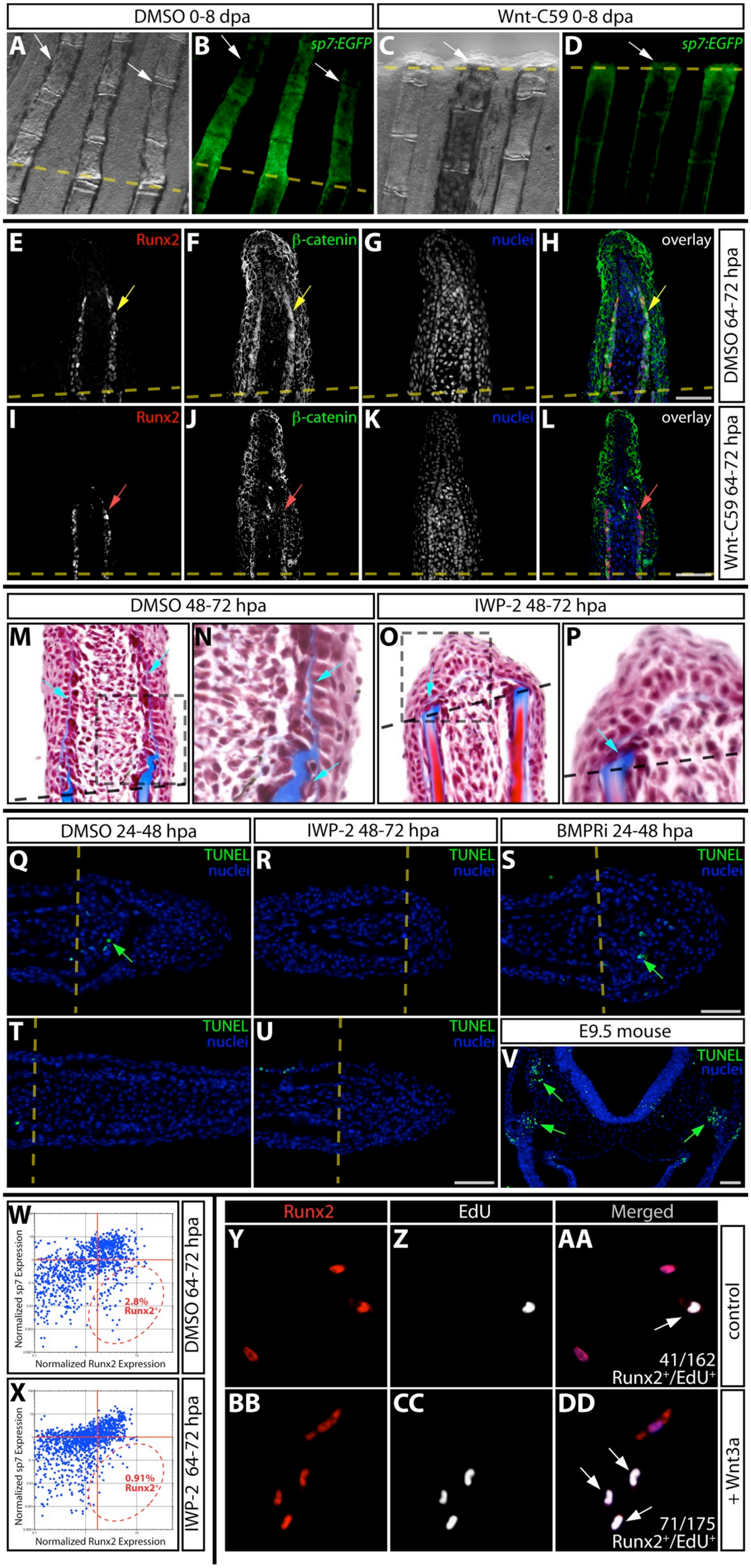

Figure S5, related to Figure 5

Figure S5, related to Figure 5. Wnt/β-catenin signaling is required for fin regeneration by supporting proliferation of Runx2$^+$ pre-osteoblasts.

(A-D) Caudal fin regeneration of *Tg(sp7:EGFP)* zebrafish treated with DMSO (A and B) or 100 nM Wnt-C59 (C and D) from 0-8 dpa. Shown are representative Rotterman contrast (A and C) and epifluorescent images (B and D) from 1 of 3 animals in each treatment group. White arrows point to osteoblasts and the dashed yellow line indicates the amputation site. **(E-L)** Immunostaining to visualize expression of Runx2 (in red) and β-catenin (in green) on fin sections from control (E-H, DMSO 64-72 hpa) and Wnt-C59 treated fish (I-L, 100 nM 64-72 hpa). Yellow arrows point to Runx2$^+$ pre-osteoblasts with accumulation of nuclear β-catenin; red arrows indicate Runx2$^+$ cells with membrane-localized β-catenin. The scale bar represents 50 μm and the dashed line is the plane of amputation. **(M-P)** Trichrome staining of control fins (M and N, DMSO from 48-72 hpa) and IWP-2 exposed fins (O and P, 10 μM from 48-72 hpa) at 72 hpa. Blue staining (Aniline Blue) indicates collagen and other connective tissue (blue arrows). The regions bounded by boxes are shown in higher magnification in the adjacent panel. **(Q-V)** TUNEL staining on fin sections from control (Q and T, DMSO 24-48 hpa), IWP-2 (R and U, 10 μM 48-72 hpa), and BMPRi (S, 5 μM 24-48 hpa) treated fish. A section from an E9.5 mouse is a positive control that serves to demonstrate the distinct labeling of apoptotic cells detected by TUNEL assays. For M-U, a representative ray from one of three fish in each treatment group is shown, with at least three rays examined from each animal. Green arrows indicate TUNEL$^+$ cells. The dashed yellow lines represent the amputation plane. Scale bars are 50 μm. **(W and X)** Scatter plots showing numbers of Runx2$^+$, Runx2$^+$/sp7$^+$ and sp7$^+$ cells in DMSO (W) or IWP-2 treated animals (X, 10 μM 64-72 hpa). Each point on the plots represents a single cell's normalized expression levels for Runx2 (x-axis) and sp7 (y-axis). Scored cells are from at least 10 sections from 4 different animals for each treatment. Regions bounded by the dashed red ellipse are defined as Runx2$^+$ cells and the percentage of Runx2$^+$ cells within the bounded region is shown. **(Y-DD)** Runx2 expression (Y and BB) and EdU incorporation (Z and CC) in cultured primary fin osteoblasts in response to Wnt3a treatment. Overlay images are shown in AA and DD. White arrows indicate Runx2$^+$/EdU$^+$ cells. Results are representative of two independent cell preparations and three independent Wnt3a treatments. In each case $p < 0.05$ (one tailed Fisher's test) comparing the fraction of EdU-incorporated Runx2$^+$ cells in control vs. Wnt3a treated cells.

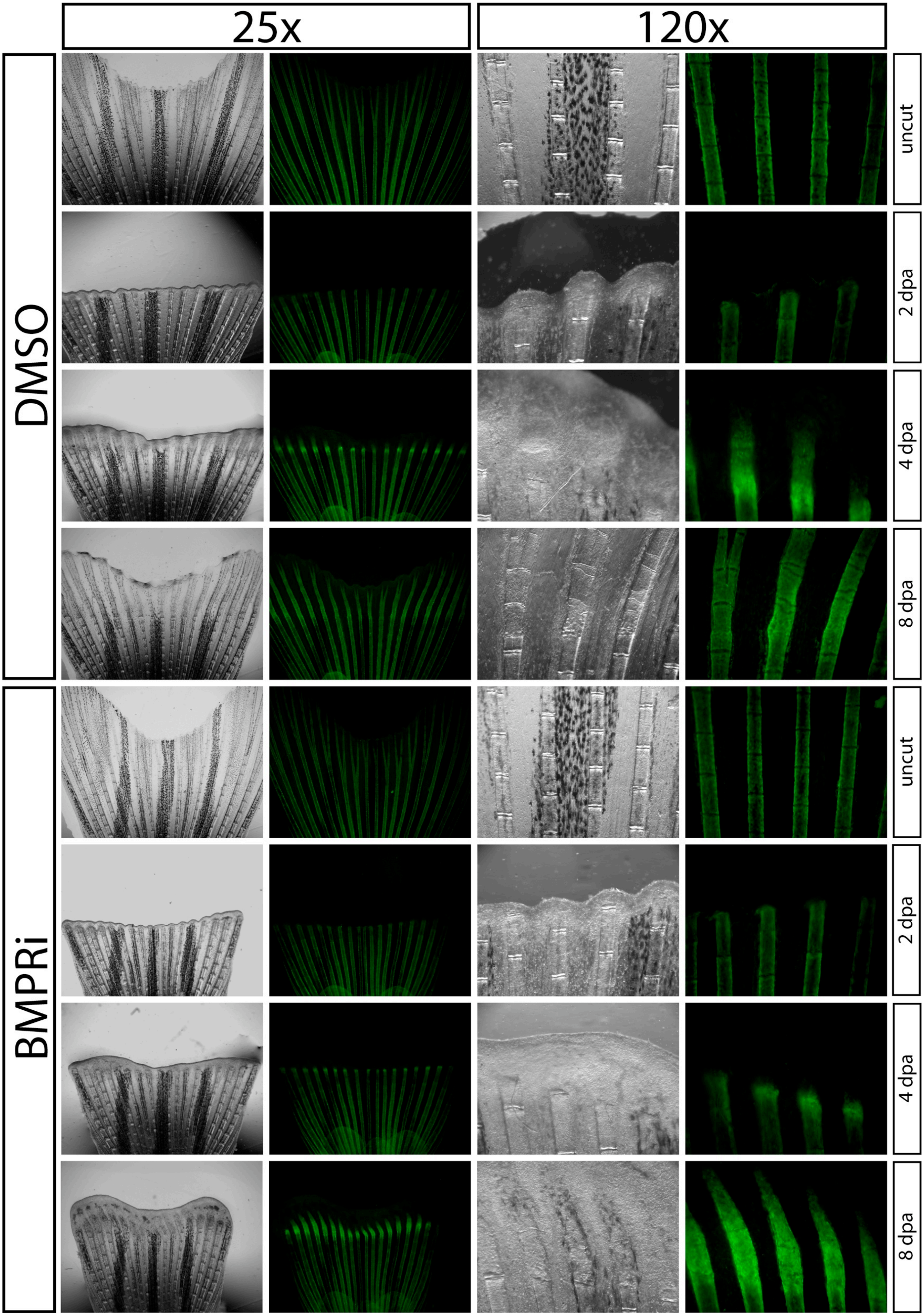

Figure S6, related to Figure 6

Figure S6, related to Figure 6. BMP receptor signaling is required for regeneration.

Regeneration of caudal fins from *Tg(sp7:EGFP)* animals exposed to DMSO (upper panels) or BMPRi (lower panels, 5 μM at 0-8 dpa). Images show the progression of regeneration in an individual fish before amputation and at 2, 4, and 8 dpa using Rotterman contrast and epifluorescence to visualize *sp7:EGFP* expression (green) in osteoblasts at 25x and 120x magnification. One of three fish, which all behaved similarly, from the two treatment groups are shown. The experiment was repeated using three independent sets of animals.

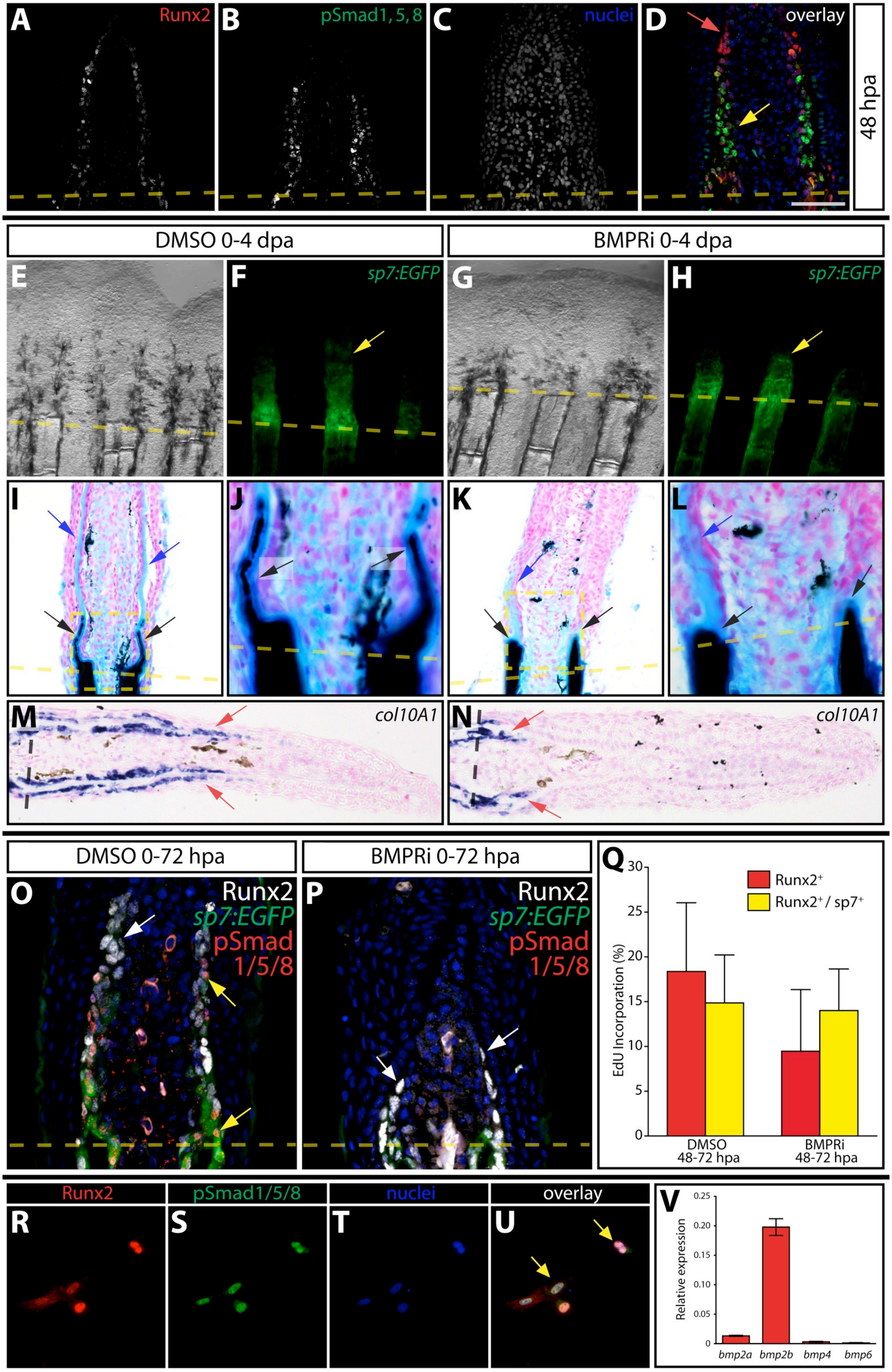

Figure S7, related to Figure 7

Figure S7, related to Figure 7. BMP signaling is essential for bone formation.

(A-D) Immunostaining of a 48 hpa fin section with pSmad 1/5/8 (green) and Runx2 (red) antibodies. The red arrow points to Runx2^{+}/pSmad^{-} pre-osteoblasts localized in the distal blastema. Yellow arrows indicate Runx2^{low}/pSmad^{+} cells. Nuclei are stained with Hoechst (blue) and the dashed line is the amputation plane. The scale bar is 50 μm. **(E-H)** Fin regeneration in *Tg(sp7:EGFP)* animals treated with DMSO (E and F) or BMPRi (G and H, 5 μM) from 0-4 dpa. Shown are representative whole-mount Rotterman contrast (E and G) and epifluorescent images (F and H) from one of the three animals in each group. Yellow arrows mark osteoblasts and the amputation site is indicated with a dashed line. **(I-L)** Von Kossa staining of frozen sections to detect calcified bone in control (I and J) and BMPRi-treated (K and L) fish. Black arrows point to calcified tissue (stained black) distal to the site of amputation (dashed yellow line); blue arrows point to regions rich in mucopolysaccharides (stained blue). The regions bounded by boxes are shown in higher magnification in the adjacent panel. Shown are representative examples from one of three fish for each treatment. (**M and N**) *col10a1* in situ hybridization on DMSO (M) and BMPRi treated (N) fins. Red arrows point to regions of gene expression stained blue. **(O and P)** Immunostaining of sections from *Tg(sp7:EGFP)* animals treated with DMSO (O) or 5 μM BMPRi (P) from 0-72 hpa) to demonstrate Runx2 (white) and *sp7:EGFP* (green) expression and levels of pSmad1/5/8 (red). Hoechst-stained nuclei are shown in blue. White arrows indicate Runx2^{+} cells and yellow arrows show *sp7:EGFP*$^{+}$/pSmad^{+} cells. The dashed yellow line indicates the amputation site. A representative section from one of three fish in each group is shown. Three or more rays were examined for each animal. **(Q)** EdU incorporation in osteoblast sub-types under DMSO or BMPRi conditions. No significant difference was observed ($p > 0.05$, two-tailed Student's t-tests). **(R-U)** Runx2 (red) and pSmad1/5/8 (green) immunostaining of primary cultured fin osteoblasts. Hoechst stained nuclei are shown in blue and the yellow arrows point to Runx2^{+} osteoblasts displaying pSmad1/5/8 immunoreactivity. For each treatment, at least 400 osteoblasts were examined from more than six comparable sections compiled from three different animals. **(V)** qRT-PCR analysis demonstrating relative transcript levels of *bmp2a*, *bmp2b*, *bmp4* and *bmp6* in cultured fin osteoblasts. Equal amounts of template were used for each reaction and relative expression levels were determined by normalizing to *rpl8* expression. Mean relative expression levels from three independent cultures are shown and the error bars indicate one standard deviation.

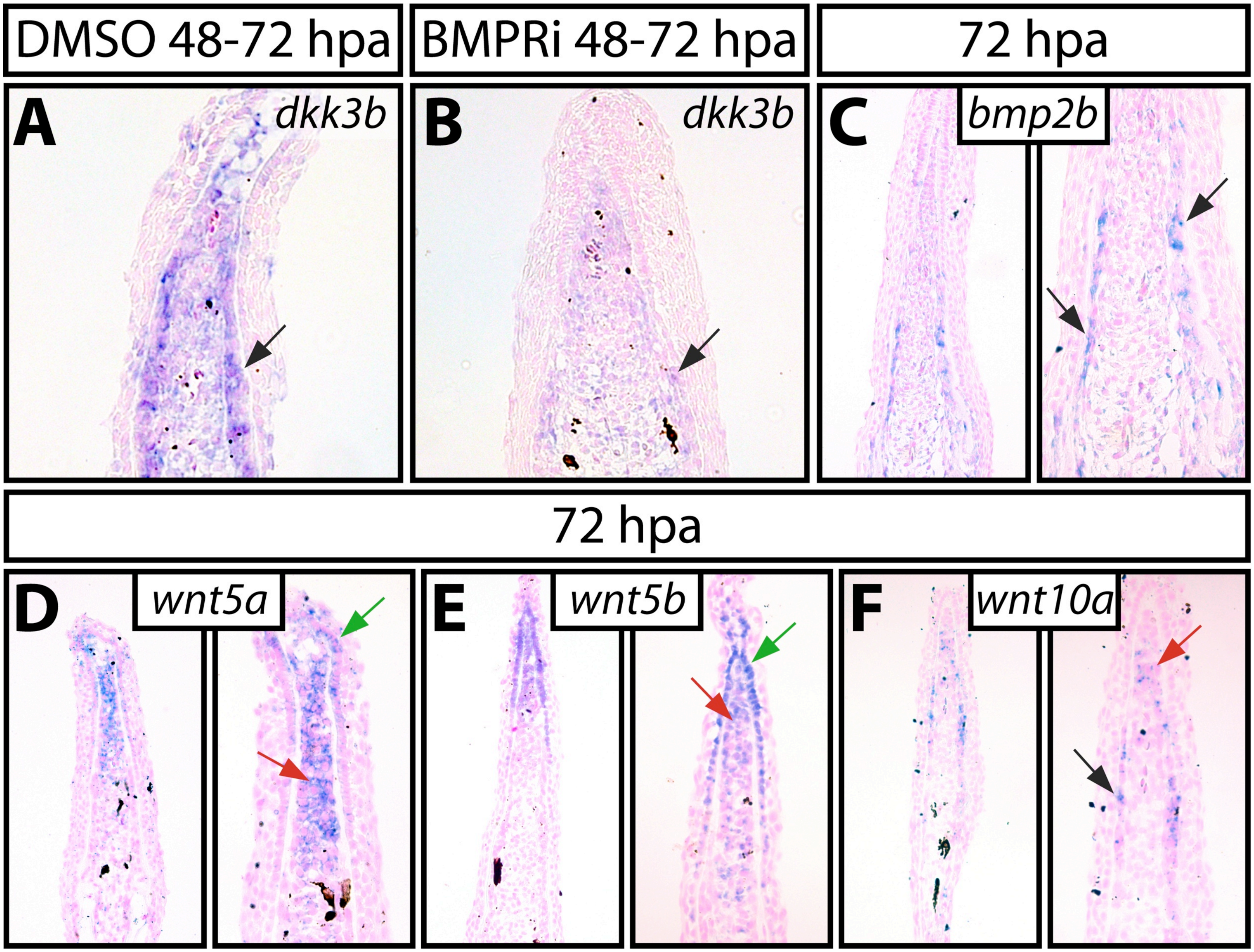

Figure S8, related to Figure 8

Figure S8, related to Figure 8. Localization of Wnt and BMP signals during fin regeneration.

(A and B) In situ hybridization using a *dkk3b* probe on 72 hpa fins from control (A, DMSO 48-72 hpa) and BMPRi (B, 5 μM, 48-72 hpa) treated fish. **(C-F)** Expression of *bmp2b* (C), *wnt5a (D), wnt5b* (E*),* and *wnt10a* (F) by in situ hybridization on paraffin sections from 72 hpa fin, shown at low and high magnification. The black arrows indicate osteoblasts, red arrows point to distal blastema mesenchymal cells, and green arrows mark basal epidermal cells.

SUPPLEMENTAL EXPERIMENTAL PROCEDURES

Immunostaining of sectioned tissue

To prepare paraffin sections, fins were fixed overnight in 4% paraformaldehyde (PFA) in PBS and then washed extensively in PBS. Fins were decalcified for 4 days in 0.5 M EDTA pH 8, which was replaced daily with fresh solution. Following decalcification, fins were rinsed extensively in PBS then dehydrated through an ethanol series and left overnight in 100% ethanol. Ethanol was replaced with xylenes followed by paraffin embedding and sectioning at 7 μm thickness.

For immunostaining, paraffin sections were rehydrated and antigen retrieval was performed in antigen retrieval buffer (1 mM EDTA pH 8, 0.1% Tween-20) for 10' in a pressure cooker. The slides were blocked with PBS/0.1% Tween-20 (PBST) and 10% non-fat dry milk and then incubated overnight at 4°C with primary antibody diluted in blocking buffer. In the case of pSmad 1/5/8 staining, PBST contained 650 mM NaCl during binding of the primary antibody. Slides were washed 3x5' in PBST and 1x30' in PBST containing 650 mM NaCl. Alexa-conjugated secondary antibodies (Invitrogen) were used at 1:1000 diluted in blocking buffer and applied to sections for 1 hour at room temperature, followed by 3 x 5' washes. Nuclei were stained with Hoechst (Invitrogen) in PBST for 10' at room temperature followed by 2 x 5' washes. Slides were mounted using Fluoro-gel (Electron Microscopy Services) and visualized with an Olympus confocal microscope. For confocal imaging, antibody-stained sections were typically analyzed at 20x and 60x magnification. Optical sections were collected and processed using ImageJ software (NIH) with maximum intensity projections generated from z-stacks. Where indicated, single optical sections were used to visualize nuclear-localized β-catenin.

For antibody staining of frozen sections, amputated fins were fixed overnight in 4% PFA/PBS, equilibrated in PBS, cryo-preserved in 30% sucrose/PBS, and frozen in agarose. Cryo-sections (16 μm) were prepared and stored at -20°C until use. Sections were hydrated in PBST and blocked in 10% non-fat dry milk in PBST + 0.1% Triton X-100 for 1-4 hours at room temperature. Subsequent staining and imaging steps followed as for paraffin sections.

Antibodies were sourced and diluted as follows: anti-Runx2 (Santa Cruz Biotechnology, 27-K) 100 ng/ml, anti-sp7 (Santa Cruz Biotechnology, A-13) 20 ng/ml, anti-β catenin (Cell Signaling, #8480) 1:250, anti-phospho Smad 1/5/8 (Cell Signaling, #9511) 1:100, anti-dsRed (Clontech, 632496) 1:200, zns-5 (Zebrafish International Resource Center (ZIRC) 1:5, anti-α-catenin (Genetex, GTX111095) 1:1000, anti-EGFP (Aves Labs, GFP-1020) 1:1000, anti-N-cadherin (BD, 610920) 1:2000.

To quantify osteoblast sub-types, 72 hpa paraffin sections, representing > 6 rays from multiple animals, were stained with Runx2 and sp7 antibodies, and imaged by confocal microscopy. Maximum intensity z-projections were generated using ImageJ (NIH) and processed in Adobe Photoshop. For scatter plot analysis, ImageJ was used to determine relative expression levels of Runx2 and sp7 in individual cells by first normalizing Runx2 and sp7 levels to nuclear staining intensity. Nuclei completely lacking expression of both Runx2 and sp7 were filtered out. Next, the mean normalized expression level was calculated for Runx2 and sp7. The deviation from the mean was determined for each nucleus by dividing the normalized expression level by the mean expression level for both sp7 and Runx2. Results were analyzed and plotted using GraphPad Prism.

Drug treatments and imaging of regenerating zebrafish

For long-term small molecule inhibitor studies, at t = 0, *Tg(sp7:EGFP)b1212* fish from the same clutch were anesthetized in Tricaine and their caudal fins were imaged on a Leica M165FC stereo microscope with epifluorescent illumination. Caudal fins were then amputated with a razor and the animals returned to fish water containing 10 μM IWP-2, 100 nM Wnt-C59, 5 μM BMPRi, or DMSO vehicle. At each indicated time point, animals were anesthetized with Tricaine and re-imaged as described above. Water, containing fresh drug or DMSO, was changed every 24 hours for the duration of the study.

Heat shock studies

Heat shock experiments were performed by amputating fins of control and *Tg(hsp70l:dkk1b-GFP)w32* animals and allowing regeneration to proceed for 48 h. At 48 hpa, fish were

transferred to a water bath and heated to 38°C for 45 minutes. Fish were removed and placed at 28°C. This heat shock regimen was repeated once more at 64 hpa and fins were collected at 72 hpa. For heat-shock studies (n = 3), cohorts of 3 or 4 animals were used and each analyzed at the completion of the study, and images shown are representative examples of each cohort.

In vivo EdU labeling

To analyze cell proliferation, fish were injected intraperitoneally with 12.5 μl of a 1 mg/ml solution of EdU (Invitrogen) in sterile saline 6 hours prior to fin harvesting. EdU was detected on paraffin-sectioned fins using the Click-iT proliferation assay kit (Invitrogen).

In situ hybridization

For in situ hybridization on slides, sections were rehydrated into PBST and then digested for 10'-30' in 2.5 μg/ml Proteinase-K (Invitrogen), re-fixed in 4% PFA/PBS for 30', and washed several times with PBST. Probes were hybridized overnight at 65°C in 5x SSC, 50% formamide, 0.1% Tween-20, 50 μg/ml heparin, and 0.5 mg/ml yeast tRNA. Slides were sequentially washed at 65°C for 30' in 2x SSC, 50% formamide, 0.1% Tween-20, 30' in 2x SSC, 0.1% Tween-20, and 30' in 0.2x SSC, and 0.1% Tween-20. Slides were incubated overnight in anti-digoxigenin (DIG) alkaline phosphatase conjugated antibodies (Roche) diluted 1:2000 in 1% blocking reagent (Roche) and developed using NBT/BCIP (Promega) at 4°C. After color development, slides were stained for 5" in Nuclear Fast Red (Vector Labs), dehydrated, mounted in Permount (Electron Microscopy Services), and imaged on a Leica microscope. For combination fluorescent in situ hybridization-immunostaining, frozen sections were treated for antigen retrieval as described above, followed by in situ hybridization with anti-DIG peroxidase conjugated antibody and developed using the Tyramide Signal Amplification (TSA) system (Perkin Elmer). Following TSA, slides were antibody stained and imaged as described in the Immunostaining section.

For whole mount in situ hybridizations, tissue was hybridized overnight at 65°C in 5x SSC, 50% formamide, 0.1% Tween-20, 50 μg/ml heparin, 0.5 mg/ml yeast tRNA and the appropriate digoxigenin (DIG) labeled riboprobe (Roche), washed and then incubated overnight in α-DIG alkaline phosphatase conjugated antibodies (Roche), and developed with NBT/BCIP (Promega).

In vitro transcription was used to generate digoxigenin (DIG)-labeled (Roche) probes. *Runx2a* (NM_212858), *bmp2b* (NM_131360), *col10a1* (NM_001083827) (Smith et al., 2006), and *sp7* (NM_212863) probes have been described previously (DeLaurier et al., 2010); *axin2* (NM_131561) probe was a gift from M. Westerfield; *GFP* probe was synthesized from pCS2-EGFP plasmid; probes for *dkk3b* (NM_001089545) 5' GGACAATAAAACCGGGAAGAC 3', 5' GGGACACATTTGGAGGTGAC 3'; *twist2* (NM_001005956) 5' ATGGAAGAGAG-TTCTAGCTCTCCCGT 3', 5' CTAGTGGGACGCAGACATCGACC 3'; *twist3* (NM_130985) 5' TAATACGACTCACTATAGGG 3', 5' TTAGTGAGTGGCGGACATGGACC 3'; *tcf7* (NM_001012389) 5' ATGCCGCAGCTGAACGGCGGA 3', 5' TTATCTGCAGGGGCCGCA-CCAGTCCG 3'; *wnt5a* (NM_001079834) 5' GGGCTCCGCGGAGAGCGCCCGGCAGATG 3', 5' TAATACGACTCACTATAGGG 3'; *wnt5b* (BC162984) 5' CTGCTGGCTCCAGCTGGC-CGACTTCC 3', 5' TAATACGACTCACTATAGGG 3'; *wnt10a* (NM_130980) 5' GGGG-AGCGCTTCTCCAAGGACTTCCTGG 3', 5' ACGGCAGTCCATTGCGCAAGGGAGGG 3' were generated by PCR amplification from regenerating caudal fin cDNA using the listed primers with all 3' primers containing a T7 priming site for in vitro transcription by T7 RNA polymerase (Fermentas).

Isolation of osteoblasts from zebrafish caudal fins

Osteoblasts were isolated by first amputating the distal end of approximately 50 adult caudal fins. The fins were then washed in PBS containing 25 μg/ml chloramphenicol and 100 μg/ml kanamycin, diced with a sterile razor, and digested for 1 hour at 30°C in Leibovitz L15 medium (Invitrogen) with 0.25% Trypsin-EDTA (Invitrogen), 25 μg/ml Liberase (Roche), and 1x Antibiotic/Antimycotic cocktail (Invitrogen). 1 ml of Fetal Bovine Serum (FBS, HyClone) was added to stop the digestions and the cells were then centrifuged at 1000 x g and resuspended in osteoblast culture medium (OM) consisting of α-MEM (Invitrogen) supplemented with 10% FBS, 1x Glutamax (Invitrogen), 1x non-essential amino acids (Invitrogen), 1x β-mercaptoethanol (Invitrogen), and 10 ng/ml acidic fibroblast growth factor (aFGF, Peprotech). The cell suspension was then passed through a 40 μm cell strainer (BD Biosciences) and plated onto a 15 cm cell culture dish. Cells were allowed to attach for 20 minutes before the medium and unattached cells were removed. Adhered cells were gently washed 3 times with PBS and osteoblast-enriched cells were recovered with 3 ml 0.25% Trypsin-EDTA for 5 minutes,

followed by adding 3 ml of OM. The cells were centrifuged as above, washed, resuspended in OM, and plated onto multi-well dishes containing coverslips coated with rat-tail collagen (Invitrogen). Cells were cultured at 30°C in 5% CO_2 and 5% O_2. To determine cell purity at 4 days post isolation, cells were fixed, immunostained with Runx2 and sp7 antibodies, and scored.

Treatment and staining of zebrafish osteoblasts

To assay β-catenin localization, zebrafish osteoblasts were maintained in OM for 48 hours before addition of either 300 nM LDN193189 (BMPRi) and/or 40 ng/ml recombinant mouse Wnt3a (R&D Systems) for 24 hours. Wnt3a dosage was determined according to the manufacturer's recommendation and dose-response pilot studies. Cells were then washed twice in PBS and fixed at room temperature for 20 minutes in 4% PFA in PBS. Cells subsequently were permeabilized in 0.2% Triton X-100 in PBS for two minutes and blocked with 5% Normal Goat Serum (NGS, MP Biomedicals) in PBS for 30 minutes. Primary antibodies used were anti-EGFP 1:1000, anti-sp7 20 ng/ml, anti-Runx2 100 ng/ml, anti-β-catenin 1:250, and anti-phospho-SMAD 1/5/8 1:500 and were applied for 1 hour at room temperature in blocking buffer. To visualize nuclei, the slides were incubated with 2 μg/ml Hoechst solution in PBS for 10-15 minutes. Coverslips were mounted with Fluoro-gel mounting medium (Electron Microscopy Services).

To quantify nuclear β-catenin levels, cells first were imaged at 40x magnification. Then, the ratio of nuclear signal as a percentage of the total β-catenin signal for individual cells was determined using FIJI software. To normalize for background signal, we imaged cells treated with secondary antibody alone. The same acquisition exposure-time parameters were used to capture images for all conditions. For fluorescence intensity measurements the following formula was used:

Corrected total cell fluorescence (CTCF) = Integrated Density – (Area of selected cell x Mean fluorescence of background)

Corrected nuclear fluorescence (CNF) = Integrated Density of Nucleus– (Area of nucleus x Mean fluorescence of background)

Percent nuclear β -catenin = (CNF/CTCF) x 100

The mean percent nuclear β-catenin from at least 25 cells from each treatment was plotted with error bars representing the standard deviation.

To examine effects of Wnt3a on proliferation, osteoblasts were cultured in OM media with either 40 ng/ml of recombinant mouse Wnt3a (R&D systems) or an equivalent concentration of PBS. Cells received fresh OM media, with or without Wnt3a, every 24 hours. After 48 hours in culture, 10 μM EdU was added and the cells were fixed in 4% PFA 12 hours later. EdU detection was performed using the Click-iT proliferation assay kit (Invitrogen). Cells were then stained with anti-Runx2 antibodies. The fraction of proliferating Runx2$^+$ osteoblasts was counted for each condition and a one-tailed Fisher's test was used to determine statistically significant differences between control and Wnt3a treated cells.

Quantitative RT-PCR

For in vitro studies, cells were isolated from caudal fins as described above. After 48 hours in culture, cells in triplicate wells were treated with 300 nM LDN193189 (BMPRi) or 0.003% DMSO as a control. After 24 hours of treatment, the media was changed, and fresh BMPRi or DMSO was added. After 96 hours in culture (48 hours of treatment), cells were washed 3x in PBS. RNA was extracted using TRIzol reagent (Invitrogen) according to the manufacturer's protocol. To analyze changes in gene expression upon in vivo inhibition of BMP signaling, 6 groups of 3 or 4 animals from the same clutch were used: 3 cohorts were treated with 5 μM BMPRi from 48 to 96 hours after amputation; similarly, 3 control cohorts were treated with the same volume of DMSO. At 96 hpa, regenerating fin tissue was dissected and RNA was extracted using TRIzol reagent (Invitrogen). cDNA was generated from total RNA using Superscript III reverse transcriptase and Oligo-dT(20) primers (Invitrogen) following the manufacturer's instructions. qPCR was performed using KAPA SYBR qPCR Master Mix (Kapa Biosystems). Relative mRNA expression levels between untreated and treated cohorts were determined using the ΔΔCt method in relation to *rpl8* mRNA abundance.

Primer sequences used for qPCR were:

runx2a (NM_212858) 5' ACGGTAATGGCTGGAAATGA 3', 5' GTCCGTCCACTGTGACC-TTT 3'; *runx2b* (NM_212862) 5' AGCTTCACCCTGACGATTACA 3', 5' CCAGTTCACTGA-GACGGTCA 3'; *sp7* (NM_212863) 5' TCCAGACCTCCAGTGTTTCC 3', 5' ATGGACATC-CCACCAAGAAG 3'; *dkk1a* (XM_001339705) 5' ACATCCCAGGAGAACCACAG 3', 5' AAACTTGTCCCTCTGTCAGCA 3'; *dkk1b* (NM_131003) 5' TCCTAAAAGAGGGCCAG-

GTC 3’, 5’ TCCCTCGACTCAAGTCTGCT 3’; *dkk2* (NM_001111209) 5’ ATCTCAGAGAGC-GCCTTGTC 3’, 5’ GCTTGCAGATTTTGGTCCAG 3’; *dkk3b* (NM_001089545) 5’ GGACAA-TAAAACCGGGAAGAC 3’, 5’ GGGACACATTTGGAGGTGAC 3’; *rpl8* (NM_200713) 5’ CCGAGACCAAGAAATCCAGA 3’, 5’ GAGGCCAGCAGTTTCTCTTG 3’; *bmp2a* (NM_131359.1) 5’ ATCAGGAGCTTCCA-CCATGA 3, 5’ TGAACGTTAATGCGGTGAAA 3’; *bmp2b* (NM_131360.1) 5’ CTGAAA-ACGATGACCCGAAC 3’, 5’ AACTGCTGCGTTGT-TTTTCC 3’; *bmp4* (NM_131342.2) 5’ AGCAGTGCCTTCAAAGGTTG 3’, 5’ CATGGGGAA-ACAGTCCATGT 3’; *bmp6* (NM_001013339.1) 5’ GGGTTGGCTGGAGTTTGAC 3’, 5’ ACGGACTTCGCTCACTTTGA 3’.

Mosaic analysis

Homozygous *Tg(Xla.ef1a1-actb2:LOXP-LOX5171-FRT-F3-EGFP,mCherry)vu295a* fish were crossed with heterozygous *Tg(dusp6:CreERT2,myl7:ECFP)b1230* animals and the resulting clutch was treated with 1 μM tamoxifen (Sigma-Aldrich) starting at 30-50% epiboly until 48 hours post fertilization (hpf). Animals containing mCherry$^+$ cells were selected and reared to adulthood. For osteoblast mosaic experiments, adult *Tg(Xla.ef1a1-actb2:LOXP-LOX5171-FRT-F3-EGFP,mCherry); Tg(dusp6:CreERT2,myl7:ECFP)* animals were selected that had caudal fins containing isolated mCherry$^+$ osteoblast mosaics. Their fins were amputated, harvested at 24, 48, or 72 hpa, and processed for immunostaining with Runx2 and dsRed antibodies on either frozen (24 and 48 hpa) or paraffin (72 hpa) sections.

TUNEL staining

A Click-iT TUNEL kit (Invitrogen) was used to detect apoptotic cells on paraffin sections from DMSO and drug treated caudal fins as directed by the manufacturer. As a positive control, sections of E9.5 mouse embryos were used. After TUNEL labeling, slides were stained with Hoechst to label nuclei and then imaged.

Histological Analysis

For Masson’s trichome staining, paraffin sections were rehydrated though a xylenes/ethanol/water series, stained using a kit as directed by the manufacturer (American Master Tech), and imaged by bright field microscopy. For Von Kossa staining, non-decalcified

16 μm frozen sections were rehydrated and stained in 1% silver nitrate solution in a Coplin jar under ultraviolet light for 20'. Un-reacted silver was removed with a 5' treatment with 5% sodium thiosulfate. Slides were then stained with Alcian blue for 30' followed by a 5' Nuclear Fast Red stain (Vector Labs), dehydrated in ethanol, cleared in xylenes, mounted, and imaged by bright field microscopy.
