# Supplementary material for: Sequential and Opposing Activities of Wnt and BMP Coordinate Zebrafish Bone Regeneration
Source: Cell Rep. Author manuscript; Available in PMC 2015 Feb 13. (PMC4009375; doi:10.1016/j.celrep.2014.01.010)
Supplement: 2 [file NIHMS558474-supplement-2.pdf]

Cell PRESS
Open ACCESS

Cell Reports
Article

Sequential and Opposing Activities of Wnt and BMP Coordinate Zebrafish Bone Regeneration

Scott Stewart,[1,*] Alan W. Gomez,[1] Benjamin E. Armstrong,[1] Astra Henner,[1] and Kryn Stankunas[1,2,*]
[1]Institute of Molecular Biology, University of Oregon, Eugene, OR 97403, USA
[2]Department of Biology, University of Oregon, Eugene, OR 97403, USA
*Correspondence: sstewart@molbio.uoregon.edu (S.S.), kryn@uoregon.edu (K.S.)
http://dx.doi.org/10.1016/j.celrep.2014.01.010
This is an open-access article distributed under the terms of the Creative Commons Attribution License, which permits unrestricted use, distribution, and reproduction in any medium, provided the original author and source are credited.

SUMMARY

Zebrafish fully regenerate lost bone, including after fin amputation, through a process mediated by dedifferentiated, lineage-restricted osteoblasts. Mechanisms controlling the osteoblast regenerative program from its initiation through reossification are poorly understood. We show that fin amputation induces a Wnt/β-catenin-dependent epithelial to mesenchymal transformation (EMT) of osteoblasts in order to generate proliferative Runx2$^+$ preosteoblasts. Localized Wnt/β-catenin signaling maintains this progenitor population toward the distal tip of the regenerative blastema. As they become proximally displaced, preosteoblasts upregulate sp7 and subsequently mature into re-epithelialized Runx2$^-$/sp7$^+$ osteoblasts that extend preexisting bone. Autocrine bone morphogenetic protein (BMP) signaling promotes osteoblast differentiation by activating *sp7* expression and counters Wnt by inducing Dickkopf-related Wnt antagonists. As such, opposing activities of Wnt and BMP coordinate the simultaneous demand for growth and differentiation during bone regeneration. This hierarchical signaling network model provides a conceptual framework for understanding innate bone repair and regeneration mechanisms and rationally designing regenerative therapeutics.

INTRODUCTION

Among mammalian organs, bones have unusually effective repair mechanisms, as demonstrated by continuous bone remodeling throughout life and the scarless healing of some fractures. Still, traumatic injuries or disease frequently exceed the innate regenerative capacities of human bone (Dimitriou et al., 2011). Envisioned restorative bone therapies include, for example, engineered cell scaffolds combined with bone grafts and the delivery of manipulated stem cells including patient-specific induced pluripotent stem cells. An appealing alternative is to recapitulate mechanisms observed in animals with remarkable capacities for self-repair, including fish and salamanders.

Osteoblasts are specialized bone producing cells that deposit a unique extracellular matrix, the osteoid, that mineralizes to form mature bone. Two transcription factors, Runx2 and sp7/Osterix (Osx), are key determinants of the osteoblast lineage (Long, 2012). *Runx2*-null mice lack all ossification (Ducy et al., 1997; Komori et al., 1997; Otto et al., 1997). Similarly, *Sp7*-deficient mice do not form bone due to a failure of osteoblasts to differentiate (Nakashima et al., 2002). *Runx2* functions upstream of *Sp7*; *Runx2*-deficient mice fail to express *Sp7*, whereas *Sp7*-null mice retain *Runx2* expression (Nakashima et al., 2002). Transcriptional control of *Runx2* and *Sp7* is mediated via cell signaling including by the Wnt and bone morphogenetic protein (BMP) pathways, both central components of bone developmental regulatory networks (Long, 2012).

Wnts are secreted proteins that function in many biological processes, including development and cancer (Clevers and Nusse, 2012). Wnts bind to Frizzled (Fz) and Lrp5/6 coreceptors culminating in the stabilization and nuclear translocation of β-catenin. Nuclear β-catenin functions with Tcf/Lef transcription factors to affect gene expression (Clevers and Nusse, 2012). Given Wnt's signaling potency in influencing cell behavior and lineage decisions, the pathway is under tight control (Clevers and Nusse, 2012). For instance, the Dickkopf family of secreted Wnt antagonists moderate Wnt activity by binding to and blocking Lrp5/6 (Mao et al., 2001). Deletion of mouse β-catenin in early mesenchymal precursors results in loss of *Runx2* and *Sp7* expression and a corresponding failure of bone formation, suggesting osteogenesis requires Wnt/β-catenin signaling (Day et al., 2005; Glass et al., 2005; Gong et al., 2001; Hill et al., 2005; Hu et al., 2005; Rodda and McMahon, 2006).

The BMP pathway is an additional key regulator of osteogenesis. Secreted BMPs bind and activate specific BMP serine/threonine kinase receptors (BMPRs). BMPRs phosphorylate Smad1/5/8 transcription factors, inducing their translocation to the nucleus where they activate downstream genes. BMPs are required for osteoblast-lineage commitment of cranial mesenchyme (Abzhanov et al., 2007), and loss of both *Bmp2* and *Bmp4* results in a severe defect in osteoblast differentiation likely due to a failure of *Runx2*-expressing cells to upregulate *Sp7* (Bandyopadhyay et al., 2006). These and earlier studies led to the development of recombinant BMPs as therapies to augment bone healing, albeit with debated clinical benefits (Garrison et al., 2010).

CrossMark

Mammalian bone fracture repair is mediated by bone-marrow-derived mesenchymal stem cells that differentiate into osteoblasts and subsequently produce remineralized bone (Dimitriou et al., 2011). Several signaling pathways, including Wnt and BMP, have been implicated in these repair processes, but their modes of action at the cellular and molecular level are largely unknown. For example, it is unclear whether Wnts and BMPs influence discrete osteoprogenitor populations or how expression of *Runx2* and *Sp7* the master regulators of osteogenesis, are coordinated during the repair process (Dimitriou et al., 2011). Even more confounding are observations that, whereas both Wnts and BMPs positively influence bone repair, the pathways appear to function in opposition to one another (Minear et al., 2010a).

Adult zebrafish fins comprise multiple organized cell types, including osteoblasts, fibroblasts, endothelial cells, neurons, and epidermal cells. Each of these cell types regenerate in concert after resection to perfectly restore the entire fin within 2–3 weeks (Gemberling et al., 2013). The bony rays, or lepidotrichia, that characterize fish fins are ossified spokes lined with bone forming osteoblasts. Lineage-tracing studies demonstrate that replacement osteoblasts are derived from preexisting unipotent osteoblasts (Knopf et al., 2011; Singh et al., 2012; Sousa et al., 2011; Stewart and Stankunas, 2012; Tu and Johnson, 2011), although an unknown population can substitute if needed (Singh et al., 2012). These observations indicate that mature osteoblasts respond to injury by acquiring progenitor cell properties, a process known as dedifferentiation. These fate restricted cells then populate lateral regions of the regenerative blastema, specialized mesenchymal tissue formed upon amputation. Subsequently, by poorly understood mechanisms, the osteoblast lineage cells undergo coordinated proliferation, differentiation, and morphogenesis to form replacement bone.

Fin regeneration is accompanied by upregulation of transcripts associated with osteogenesis including *runx2a*, *runx2b*, and *sp7* (Knopf et al., 2011; Smith et al., 2006; Sousa et al., 2011). Transgenic lines utilizing fish *sp7* promoter/enhancer elements indicate that fin osteoblasts distal to the amputation site express *sp7*, whereas cells below the amputation site downregulate *sp7* expression (DeLaurier et al., 2010; Knopf et al., 2011; Singh et al., 2012). Similarly, human *Runx2* regulatory elements drive reporter gene expression in cells distal to the amputation site (Knopf et al., 2011). Although the Wnt/β-catenin pathway is required for fin regeneration and components of the Wnt pathway are expressed in the regenerating fin (Kawakami et al., 2006; Poss et al., 2000; Stoick-Cooper et al., 2007), it remains unknown which cell types participate in Wnt/β-catenin signaling or how the pathway promotes regeneration. Similarly, BMP gene expression patterns and overexpression of the BMP inhibitor Chordin implicate the BMP pathway in fin osteoblast differentiation (Smith et al., 2006). However, it is unresolved how BMP promotes bone differentiation or even when and where BMP signaling is active during regeneration.

Here, we examine mechanisms of regenerative osteogenesis in the zebrafish caudal fin. Upon fin amputation, Runx2^{+} preosteoblasts accumulate in the blastema following a Wnt/β-catenin dependent epithelial-to-mesenchymal transformation (EMT) of osteoblasts lining the damaged bone. Distal Wnt signaling continuously maintains Runx2^{+} preosteoblasts, whereas their differentiation into mature, sp7^{+} osteoblasts is promoted by autocrine BMP signaling. By inducing secreted Wnt antagonists, including *dkk1b*, BMP facilitates negative crosstalk between differentiating osteoblasts and preosteoblasts. This straightforward signaling network produces a hierarchically and spatially organized osteoblast lineage that balances a simultaneous need for bone growth and differentiation until fin regeneration is complete.

RESULTS

Runx2 and sp7 Define a Hierarchical Organization of Osteoblast Lineage Cells during Fin Regeneration

We immunostained paraffin-sectioned fins with antibodies directed against the osteogenic transcription factors, Runx2 and sp7, at various times postamputation. At 24 hr postamputation (hpa), osteoblasts lining preexisting bone adjacent to the amputation site upregulated expression of Runx2. Further, Runx2, but not sp7, expressing cells were found distal to the amputation site distributed in the mesenchymal tissue of the forming blastema (Figures 1A–1C). At 32 hpa, sp7 expression was induced in select Runx2^{+} mesenchymal cells (Figures 1D–1F). By 48 hpa, osteoprogenitors became more linearly organized adjacent to the basal epidermis with Runx2^{-}/sp7^{+} cells first becoming detected near the amputation site (Figures 1G–1I). At 72 hpa, the osteoblast lineage was highly organized along the proximal-distal axis (Figures 1J–1L). Runx2^{+} cells were always the most distally localized population and were neighbored by Runx2^{+}/sp7^{+} cells. In contrast, sp7^{+} cells were concentrated near the amputation site. Similarly, *runx2a* and *sp7* transcripts were enriched at the distal and proximal ends of the blastema, respectively (Figures 1M and 1N).

We quantified osteoblast subtypes and their proliferation state by delivering 5-ethynyl deoxyuridine (EdU) to label cells in S phase 6 hr prior to harvesting fins at 72 hpa. In stained section fins, we observed robust EdU labeling in the distal blastema mesenchyme but only rarely in the epidermis (Figures 1O and 1P), in agreement with a previous report (Smith et al., 2006). Runx2^{+} and Runx2^{+}/sp7^{+} cells were found in equal proportion (Figures 1O, 1P, and 1Q, ~45%) with significantly fewer sp7^{+} cells (Figure 1Q, ~8%, $p < 10^{-5}$). More Runx2^{+} cells incorporated EdU compared to sp7^{+} cells (16% versus 7%) (Figure 1Q, $p < 0.03$). We also routinely saw clusters of several high-expressing Runx2^{+} cells that were EdU^{-}, suggesting there are distinct proliferative and nonproliferative Runx2^{+} preosteoblasts (Figure 1P, red arrows). The temporal and spatial patterns of Runx2 and sp7 expression and osteoblast-subtype proliferation demonstrate a hierarchical organization of the osteoblast lineage in regenerating fins. Renewing Runx2^{+} preosteoblasts at the leading edge of the fin beget proliferative Runx2^{+}/sp7^{+}-differentiating cells. These cells subsequently mature into relatively quiescent sp7^{+} osteoblasts that append to progressively elongating bone.

A *twist2*-Associated Epithelial-to-Mesenchymal Transition Generates Preosteoblasts

We performed lineage tracing to confirm the distal, leading edge Runx2^{+} cells were derived from preexisting osteoblasts.

Cell Press
Open ACCESS

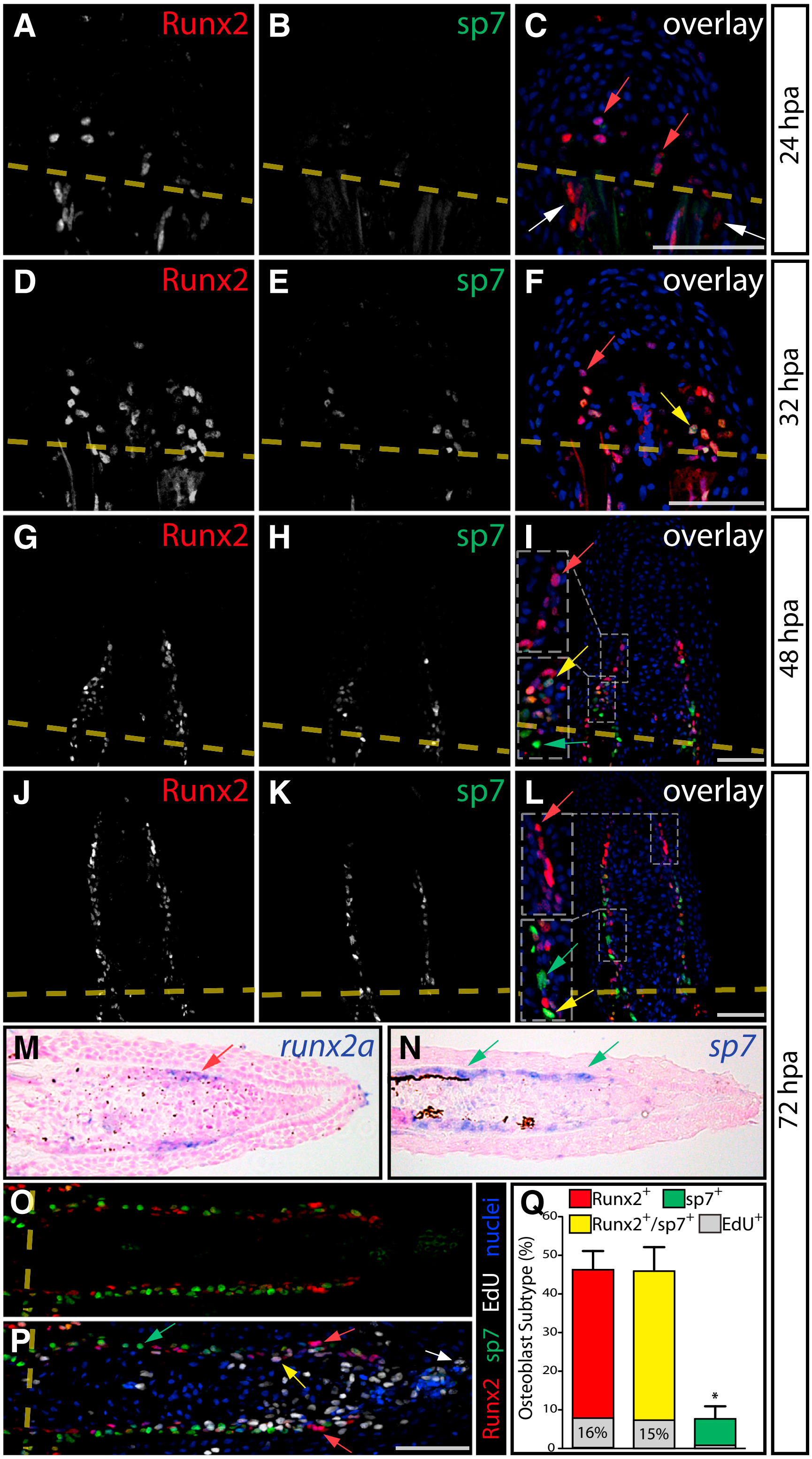

Figure 1. Runx2 and sp7 Define Distinct Populations of Preosteoblasts in Regenerating Fins

(A–L) Runx2 (red) and sp7 (green) immunostaining at 24 (A–C), 32 (D–F), 48 (G–I), and 72 (J–L) hr post-amputation (hpa) on longitudinal caudal fin sections. Images are maximum intensity projections of confocal z-stacks. Amputation planes are indicated with a dashed yellow line, white arrows point to Runx2$^+$ cells proximal to the amputation site, red arrows indicate Runx2$^+$ cells distal to the amputation site, yellow arrows denote Runx2$^+$/sp7$^+$cells, and green arrows show sp7$^+$ cells. For (I) and (L), regions bound by dashed white boxes are shown in higher magnification in inset panels.

(M and N) RNA in situ hybridizations showing *runx2a* and *sp7* expression on fin sections harvested 72 hpa. Red and green arrows point to lateral blastema cells expressing *runx2a* and *sp7*, respectively.

(O and P) Immunostaining showing Runx2 (red) and sp7 (green) expression (O) and incorporation of EdU (P, white, 6 hr pulse) on 72 hpa fin sections. The white arrow points to an extreme distally located EdU$^+$ blastema cell. The yellow and green arrows indicate Runx2$^+$/EdU$^+$ and sp7$^+$/EdU$^-$ cells, respectively. Red arrows show high Runx2$^+$/EdU$^-$ cells. In all overlay images, Hoechst-stained nuclei are shown in blue. The scale bars represent 50 μm.

(Q) Quantitation of osteoblast subtypes and EdU incorporation at 72 hpa. Bars show the mean percentile representation of osteoblast subtypes on comparable sections (n = 12 rays, compiled from >4 fish). Error bars are one SD from the mean, and significant p values are indicated with an asterisk ($p < 10^{-5}$, Student's t tests comparing either Runx2$^+$ or Runx2$^+$/sp7$^+$ populations to sp7$^+$ cells). The proportion of each cell population that incorporated EdU is indicated by the extent of gray shading relative to the bar's height. Fewer sp7$^+$ relative to Runx2$^+$ cells incorporated EdU ($p < 0.03$, one-tailed Fisher's exact test, n = 547 Runx2$^+$ and 75 sp7$^+$ cells).

We generated adult zebrafish with caudal fins containing isolated fin rays lined with permanently labeled mCherry$^+$ osteoblasts (Stewart and Stankunas, 2012) (Figure 2A). At 72 hpa, fins from the same animal displayed abundant mCherry$^+$ osteoblasts in the regenerating tissue (Figure 2B). Immunostaining showed that Runx2$^+$ cells extending from labeled hemirays coexpressed mCherry (Figures 2C–2H). Likewise, Runx2$^+$ blastema cells observed at 24 or 48 hpa were also derived from mosaic-labeled osteoblasts (Figure S1). These results demonstrate that fin amputation triggers the migration of osteoblasts closely associated with the bone ray into the nascent blastema following their upregulation of Runx2. These Runx2$^+$ blastema cells are the precursors to redifferentiated osteoblasts that will line and mineralize replacement bone.

We hypothesized that fin amputation induces an epithelial-to-mesenchymal transformation (EMT) of osteoblasts, which display an underappreciated epithelial-like organization (Ferrari et al., 2000; Izu et al., 2011), to generate Runx2$^+$ preosteoblasts. We immunostained nonregenerating fins with zns-5 (Johnson and Weston, 1995), α-catenin, and β-catenin antibodies to label osteoblasts (zns-5) and adherens junctions that interconnect epithelial sheets (catenins). Zns-5-positive osteoblasts expressed both α- (Figures 2I–2L) and β-catenin (Figure S2A), which were concentrated at membranes separating individual osteoblasts, demonstrating that fin ray osteoblasts have epithelial-like properties.

We examined α-catenin localization in 96 hpa fins from transgenic *Tg(sp7:EGFP)b1212* animals, which express EGFP in mature and differentiating osteoblasts (DeLaurier et al., 2010). We saw membrane-localized α-catenin staining in both Runx2$^+$/*sp7:EGFP*$^+$ and *sp7:EGFP*$^+$ cells that were in close proximity to new bone. In contrast, distally located Runx2$^+$ cells did not express α-catenin, suggesting they lacked adherens junctions (Figures 2M–2R). N-cadherin, another component of adherens junctions (Lim and Thiery, 2012), also was expressed in maturing osteoblasts (Figures S2B–S2E). At 24 hpa, we observed α-catenin staining only in the fin epidermis (Figures 2S and 2T), indicating that osteoblasts rapidly lose α-catenin expression as they populate the blastema and acquire a progenitor state. Further indicative of EMT, osteoblasts changed cell shape from strikingly long and thin epithelial cells lining nonregenerating bone to a more compact, polygonal morphology when they became Runx2$^+$ and Runx2$^+$/sp7$^+$ blastema-populating cells (compare Figures S3E and S3F).

EMTs are directed by transcription factors including Snail/Slug and Twist (Lim and Thiery, 2012). In developing bone, Twist1 and Twist2 are expressed in Runx2$^+$ preosteoblasts where Twist interacts with Runx2 (Bialek et al., 2004; Fulzele et al., 2010; Tran et al., 2010). RNA in situ hybridization showed that both *twist2* and *runx2a* were induced at 24 hpa in tissue immediately adjacent to the amputation site (Figures 2U and 2V). Combining fluorescent in situ hybridization with Runx2 immunostaining on 72 hpa sections succinctly demonstrated that distal Runx2$^+$ cells coexpressed *twist2* mRNA (Figures 2W–2Y) and are thus bona fide mesenchymal cells. Similar to *twist2*, *twist3* was expressed at 24 hpa adjacent to the amputated bone (Figure S2G) and by 72 hpa was distally localized in a pattern reminiscent of *twist2* (Figures S2F and S2H). We conclude that Runx2$^+$ cells originate from an EMT of epithelial-organized differentiated osteoblasts present near the amputation site, and, throughout regeneration, distal Runx2$^+$ preosteoblasts are maintained in a mesenchymal *twist2/3*-expressing state. However, the initial induction of Runx2 expression does not appear to depend on EMT, because epithelial osteoblasts lining the bone proximal to the amputation site also upregulate Runx2$^+$ (Figure 1).

Wnt/β-Catenin Signaling Is Active in Preosteoblasts

Wnts promote EMT (Stark et al., 1994), osteogenesis (Day et al., 2005; Hill et al., 2005; Hu et al., 2005), and fin regeneration (Poss et al., 2000; Kawakami et al., 2006; Stoick-Cooper et al., 2007). We monitored Wnt signaling in regenerating fins by examining β-catenin localization by antibody staining. At 24 and 32 hpa, we saw Runx2$^+$ cells with diffuse nuclear β-catenin localization dispersed in the mesenchyme distal to the amputation site (Figures 3A–3F). Nuclear β-catenin was still apparent at 48 hpa when Runx2$^+$ cells became organized along the anterior-posterior body axis (Figures 3G–3I). At 72 hpa, strong nuclear β-catenin staining persisted in distally residing Runx2$^+$ preosteoblasts, with less nuclear β-catenin in maturing osteoblasts located closer to the amputation site (Figures 3J–3L), suggesting that downregulation of Wnt signaling accompanies osteoblast differentiation. At 96 hpa, *Tg(sp7:EGFP)* animals showed robust nuclear β-catenin primarily in distal Runx2$^+$ cells and not in proximal osteoblasts that expressed only *sp7:EGFP* (Figures 3M–3R and S3A–S3D). High-magnification single optical sections confirmed overlapping nuclear β-catenin and Runx2 in distal osteoblasts at 96 hpa (Figures 3S–3U). Expression of the Wnt transcriptional effector *tcf7* was also enriched in Runx2$^+$ osteoblasts (Figures 3V–SX, S3G, and S3H), supporting the potential of Runx2$^+$ cells to respond to Wnt signals.

We examined *axin2* expression, a well-accepted target gene of canonical Wnt signaling (Clevers and Nusse, 2012), to monitor Wnt-responsive cell types in the regenerating fin. At 72 hpa, *axin2* was strongly expressed in distal Runx2$^+$ preosteoblasts (Figures 3Y–3AA, S3I, and S3J). *Axin2* transcripts additionally were detected in Runx2$^-$ cells directly medial to the preosteoblasts and extending to the extreme tip of the blastema. These nonosteoblasts also showed low levels of nuclear β-catenin (Figures S3A–S3D). We further examined Wnt activity using *Tg(TOP:GFP)w25* transgenic fish that express destabilized GFP under the control of a synthetic Wnt-responsive promoter (Dorsky et al., 2002). Due to the rapid turnover of the destabilized GFP, we monitored *GFP* expression by in situ hybridization. At 72 hpa, *TOP:GFP* was found in cells of the distal blastema, including Runx2$^+$ preosteoblasts, in a pattern indistinguishable from *axin2* (Figures 3BB–3DD). We did not observe nuclear β-catenin, *axin2* or *TOP:GFP* expression in the epidermis, suggesting that Wnt/β-catenin is not a major regulator of fin epidermis regeneration even though the basal epidermis expresses the Wnt effector *lef1* (Poss et al., 2000). Collectively, the striking nuclear β-catenin, *axin2* expression, and *TOP:GFP* activity in leading edge Runx2$^+$ cells suggest ongoing roles for canonical Wnt signaling in regulating Runx2$^+$ preosteoblasts from their earliest EMT-driven emergence following amputation through later stages of regenerative outgrowth. Our data further indicate that nonosteoblast lineage cells in the distal blastema represent an additional node of Wnt/β-catenin signaling.

Wnt Production and Signaling Is Required for Osteoblast EMT

Wnt is one secreted factor that can initiate EMT during embryonic development (Lim and Thiery, 2012; Stark et al., 1994), and Wnts induce *twist* expression during mouse craniofacial bone development (Tran et al., 2010). We performed loss-of-function studies of Wnt signaling during fin regeneration using IWP-2, a small molecule inhibitor of Porcupine, an acyltransferase that covalently modifies Wnts and is required for their secretion (Chen et al., 2009). We treated *Tg(sp7:EGFP)* animals

Cell PRESS
Open ACCESS

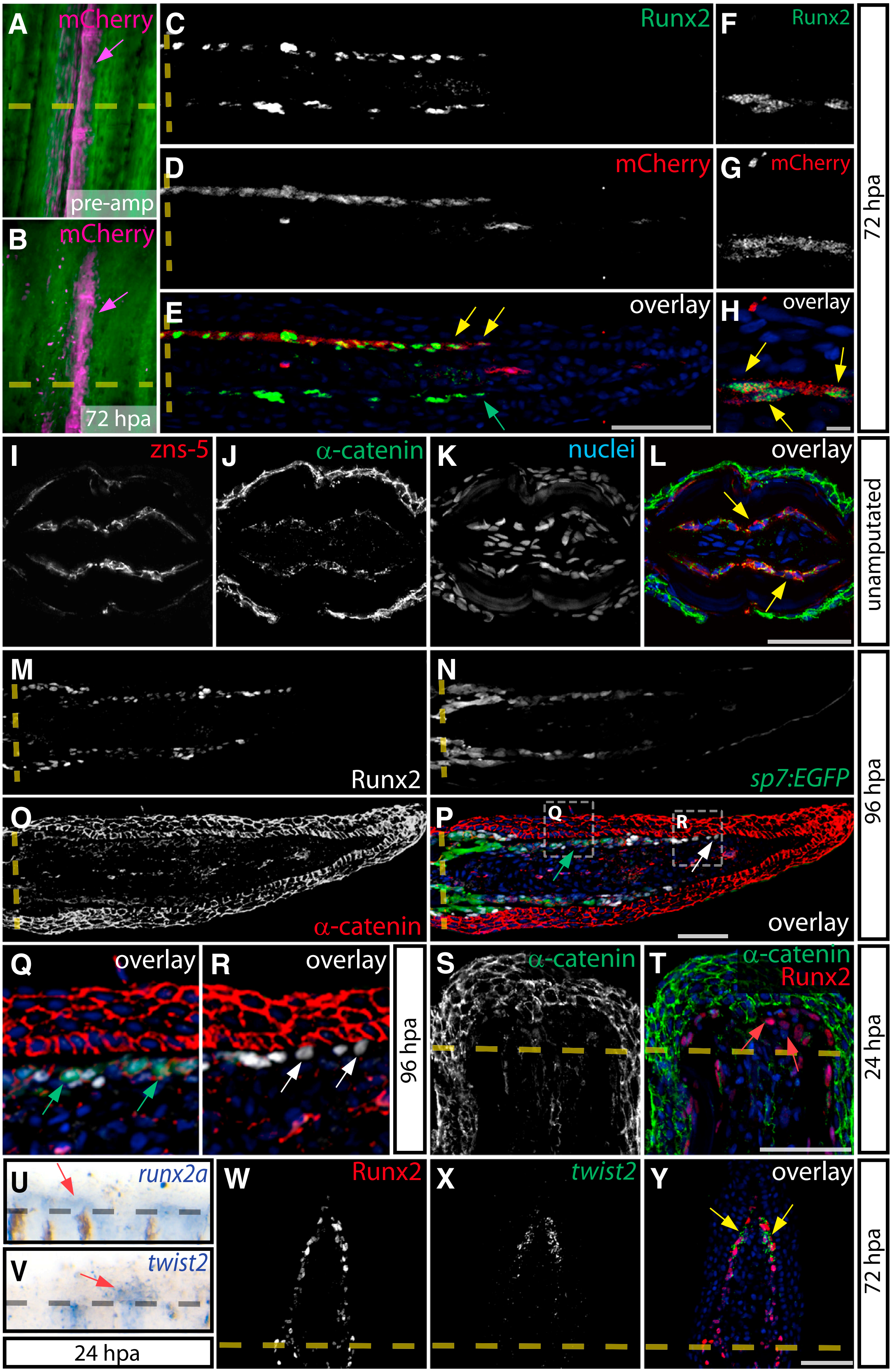

(legend on next page)

with IWP-2 and monitored regeneration in individual animals from 0 to 8 dpa. At 2 and 4 dpa, IWP-2-treated fish arrested regeneration after forming a wound epidermis and lacked *sp7:EGFP*$^{+}$ osteoblasts beyond the amputation site (Figure S4). By 8 dpa, control animals had substantially regenerated and *sp7:EGFP*$^{+}$ osteoblasts were seen throughout the regenerate (Figures 4A–4D and S4). In contrast, IWP-2-treated fish displayed no regenerative outgrowth, although bone proximal to the amputation site continuously maintained *sp7:EGFP*$^{+}$ expression (Figures 4E–4H and S4). Low doses (100 nM) of Wnt-C59, a chemically distinct Porcupine inhibitor (Proffitt et al., 2013), also completely inhibited bone regeneration (Figures S5A–S5D).

Given the complete lack of bone regeneration in both IWP-2- and Wnt-C59-treated fish, we speculated that an initiating step(s) requires Wnt activity. Sections of fins from fish treated with IWP-2 from 0 to 24 hpa lacked both nuclear-localized β-catenin and Runx2-expressing blastema cells (Figures 4I–4P). However, Runx2 expression was still evident in bone-lining osteoblasts near the amputation site, suggesting its initial activation is Wnt-independent (Figures 4I–4P). A later treatment with IWP-2 from 48 to 72 hpa abolished *twist2* expression (Figures 4Q and 4R). These results demonstrate that Wnt initiates osteoblast EMT and then maintains the mesenchymal state of preosteoblasts by promoting *twist2* expression.

Wnt/β-Catenin Signaling Maintains Runx2^{+} Preosteoblasts

We tested if Wnt has an ongoing role maintaining preosteoblasts during bone regrowth by blocking Wnt production from 48 to 72 hpa using IWP-2. This IWP-2 regimen stunted overall regeneration and depleted osteoblast-lineage cells distal to the amputation site (Figures 5A–5D). The few remaining cells displayed membrane-localized β-catenin and reduced levels of Runx2 (Figures 5A and 5B), whereas sp7 expression was largely unchanged (Figures 5C and 5D). Along with fewer osteoblasts, fins from IWP-2-treated fish had a pronounced decrease in collagen deposition near amputated bones (Figures S5M–S5P). Although the small number of osteoblasts remaining after a 24 hr IWP-2 treatment precluded a detailed analysis, we were able to rule out apoptosis as a cause of the phenotype by TUNEL staining (Figures S5Q–S5V).

At least two populations of distally located cells transmit Wnt signals in the regenerating fin: Runx2^{+} preosteoblasts and adjacent nonosteoblast distal mesenchymal cells. Therefore, the loss of osteoblasts after 24 hr IWP-2 treatment could represent direct and/or indirect Wnt signaling roles. To distinguish between these possibilities, we inhibited Wnt for brief periods (8 hr) during regeneration and monitored β-catenin, Runx2, and sp7 by immunostaining. Administration of IWP-2 or Wnt-C59 from 64 to 72 hpa was sufficient to inhibit nuclear localization of β-catenin (Figures 5E and 5F and S5E–S5L). An examination of osteoblast subtypes (Figures 5G–5J) indicated both IWP-2- and Wnt-C59-treated fish contained fewer Runx2^{+} cells (Figure 5I, $p < 0.02$) and increased numbers of Runx2^{+}/sp7^{+} cells (Figure 5I, $p < 0.02$). However, no significant difference in the number of sp7^{+} cells was observed (Figure 5I). EdU incorporation in Runx2^{+} cells, but not sp7-expressing osteoblasts, was also reduced in fish treated with either IWP-2 or Wnt-C59 (Figure 5J, $p < 0.003$), indicating that Wnt-dependent proliferation specifically maintains Runx2^{+} preosteoblasts. We also quantified Runx2 and sp7 expression on sections from multiple animals and produced scatterplots depicting normalized expression levels in individual cells. Confirming our qualitative observations, there was a 3.1-fold decrease in the fraction of Runx2 single positive cells relative to all mesenchymal cells in animals treated with IWP-2 from 64 to 72 hpa (Figures S5W and S5X).

We developed a method to isolate and culture primary fin osteoblasts where 78.5% of the cells stained positively for Runx2 and/or sp7 (SD = 8.3%, n = 5 independent cell preparations). To test if Wnt directly promotes fin osteoblast proliferation, we treated these cultures with Wnt3A and monitored EdU incorporation in Runx2^{+} cells. Wnt3A significantly increased the fraction of EdU^{+}/Runx2^{+} cells (Figures S5Y–S5DD, $p = 0.0021$). We conclude that, in regenerating fins, continuously secreted Wnt activates canonical Wnt signaling in Runx2^{+} preosteoblasts to support, directly or indirectly, their renewal.

BMP Signaling Promotes sp7 Expression and Osteoblast Differentiation during Bone Regeneration

BMPs have been implicated in osteoblast differentiation (Abzhanov et al., 2007; Bandyopadhyay et al., 2006) and are expressed in fin osteoblasts during regeneration (Smith et al., 2006). We

Figure 2. Dual Epithelial/Mesenchymal Nature of Osteoblasts during Fin Regeneration

(A–H) Whole-mount imaging of a mosaic bone ray before amputation (A) and at 72 hpa (B) showing osteoblasts permanently labeled by Cre-mediated expression of mCherry (magenta, indicated by magenta arrows). Sections of the same ray at 72 hpa immunostained for Runx2 (C and F) and mCherry (D and G). Overlays are shown in (E) and (H). Yellow arrows show distal mCherry^{+}/Runx2^{+} cells, and the green arrow points to a mCherry^{-}/Runx2^{+} distal osteoblast (C–H). Amputation sites are indicated with a dashed line. (F)–(H) are high-magnification images of another section through the same ray in (C)–(E).

(I–L) Antibody-stained transverse sections of nonregenerating fins showing α-catenin (green) and zns-5 (red). Yellow arrows indicate epithelial α-catenin^{+}/zns-5^{+} osteoblasts.

(M–R) A section from a 96 hpa *Tg(sp7:EGFP)* fin showing Runx2^{+} (white), *sp7:EGFP* (green), and α-catenin (red) expression. Regions bounded by dashed white boxes in (P) are shown at higher magnification in (Q) and (R). White arrows point to Runx2^{+} preosteoblasts and green arrows show *sp7:EGFP*$^{+}$ osteoblasts with membrane-localized α-catenin.

(S and T) Expression of Runx2 (red) and α-catenin (green) on 24 hpa sections. Red arrows indicate Runx2^{+}/α-catenin^{-} mesenchymal cells.

(U and V) Whole-mount RNA in situ hybridizations of *runx2a* (U) and *twist2* (V) at 24 hpa. Red arrows denote the specific expression of *runx2a* and *twist2* in regenerating tissue.

(W–Y) Runx2 immunostaining (W, red) and *twist2* in situ hybridization (X, green) is shown on 72 hpa frozen sections and overlaid (Y). Yellow arrows point to *twist2*$^{+}$/Runx2^{+} preosteoblasts. Hoechst-labeled nuclei are in blue.

Scale bars represent 50 μm except in H, which represents 5 μm.

Cell PRESS
Open ACCESS

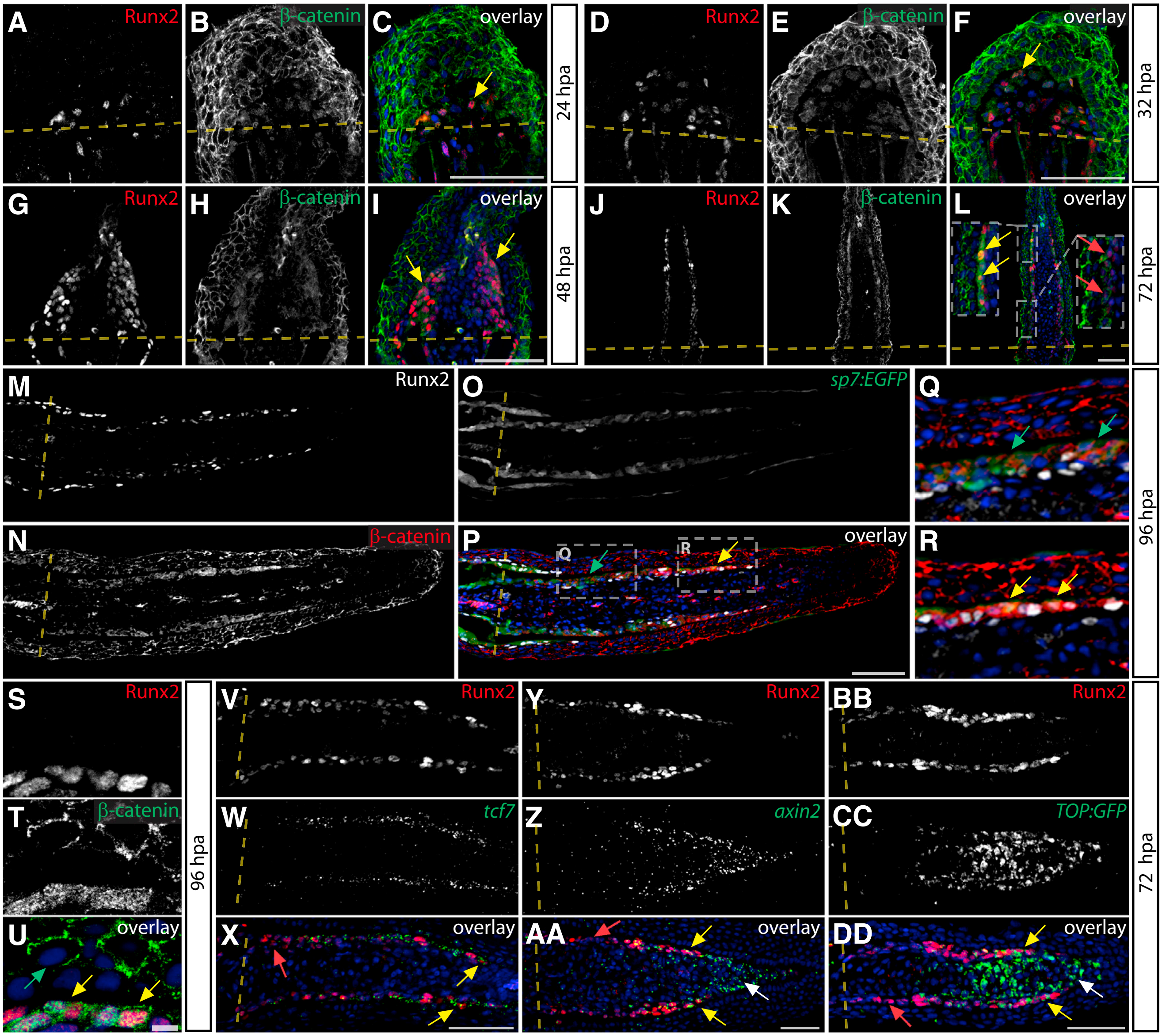

Figure 3. Wnt/β-Catenin Signaling in Preosteoblasts during Regeneration

(A–L) Immunostaining of Runx2 (red) and β-catenin (green) on fin sections at 24 (A–C), 32 (D–F), 48 (G–I), and 72 (J–L) hpa. Yellow arrows indicate Runx2^{+} cells with nuclear β-catenin, and the red arrows point to proximal osteoblasts at 72 hpa with membrane-localized β-catenin. For (L), regions bounded by dashed white boxes are shown in higher magnification in inset panels.

(M–R) Expression of Runx2 (M, white), *sp7:EGFP* (O, green), and localization of β-catenin (N, red) is shown and overlaid (P–R) on 96 hpa *Tg(sp7:EGFP)* section. Regions bound by dashed white boxes in (P) are shown at higher magnification in (Q) and (R). Yellow arrows highlight Runx2^{+} cells with nuclear-localized β-catenin, and green arrows indicate membrane-localized β-catenin in *sp7:EGFP*$^{+}$ osteoblasts.

(S–U) A single optical section at 96 hpa fin showing immunostaining of Runx2 (red) and β-catenin (green) in distal preosteoblasts. Yellow arrows point to Runx2^{+} nuclei containing β-catenin, and the green arrow indicates exclusively membrane-localized β-catenin in the fin epidermis.

(V–X) Runx2 immunostaining (V, red) and *tcf7* in situ hybridization (W, green) on a 72 hpa cryosection (X, overlay). The yellow arrow indicates distal Runx2^{+} preosteoblasts that coexpress *tcf7*; the red arrow indicates proximal osteoblasts lacking *tcf7* expression.

(Y–AA) Runx2 immunostaining (Y, red) combined with *axin2* in situ hybridization (Z, green) is shown on a 72 hpa cryosection and overlaid (AA). Yellow arrows point to distal preosteoblasts coexpressing *axin2* and Runx2. The white arrow indicates distal nonosteoblast blastema cells that express *axin2*. The red arrow shows proximal osteoblasts without *axin2* expression.

(BB–DD) Runx2 immunostaining (BB, red) and *GFP* in situ hybridization (CC, green) on a 72 hpa section from a *Tg(TOP:GFP)* fin shown in overlay (DD). Yellow arrows show Runx2^{+}/GFP^{+} preosteoblasts. The white arrow indicates distal nonosteoblast blastema cells that express the reporter. Hoechst-stained nuclei are shown in blue. Scale bars represent 50 μm except in U, which represents 5 μm.

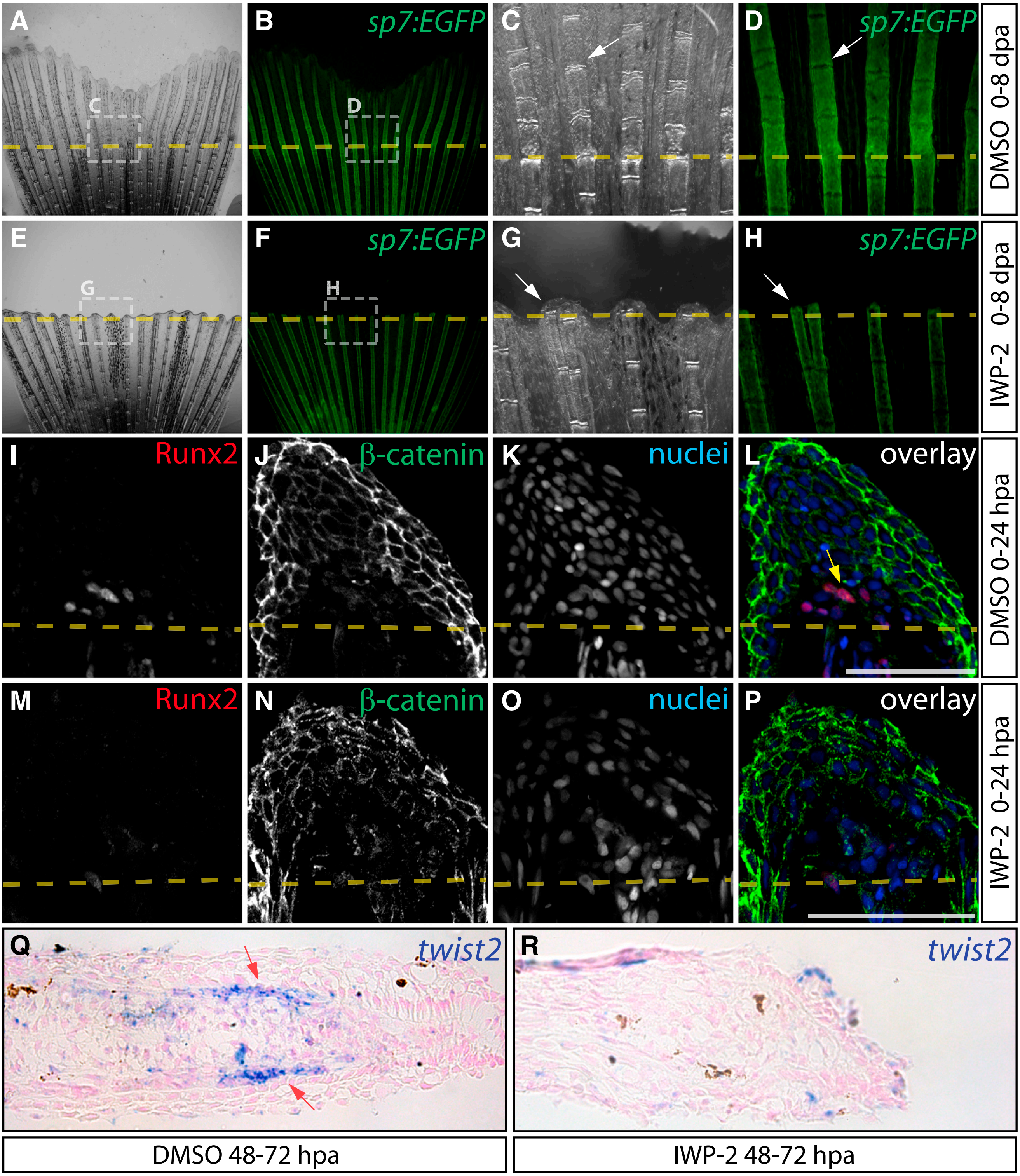

Figure 4. Wnt/β-Catenin Is Required for Osteoblast EMT and Dedifferentiation

(A–H) Regeneration of *Tg(sp7:EGFP)* fins after treatment with DMSO (A–D) or IWP-2 (E–H, 10 μM from 0 to 8 dpa). Rotterman contrast (A, C, E, G) and epifluorescence (B, D, F, H) images show *sp7:EGFP* expression in osteoblasts (white arrows) before amputation (A, B, E, F) and at 8 dpa (C, D, G, H). Shown are 25× images from one of three fish for control and IWP-2 groups and regions within dashed white boxes are shown at 120× magnification.

(legend continued on next page)

examined the pattern of BMP activity by staining fin sections from 96 hpa *Tg(sp7:EGFP)* fish with antibodies against phosphorylated, active Smad1, 5, and 8 (pSmad1/5/8), Runx2, and EGFP. We observed pSmad1/5/8 in differentiating *sp7:EGFP*$^+$ cells but not Runx2$^+$/*sp7:EGFP*$^-$ preosteoblasts or other cell types (Figure 6A). pSmad1/5/8 staining was evident by 48 hpa, coincident with the appearance of sp7$^+$ maturing osteoblasts (Figures S7A–S7D). The discrete localization of pSmad1/5/8 suggests that differentiating osteoblasts are the primary targets of BMP signals during fin regeneration and that the pathway promotes osteoblast redifferentiation.

We blocked BMP signaling in *Tg(sp7:EGFP)* animals using the BMPR inhibitor (BMPRi) LDN193189 (Cuny et al., 2008) from 0 to 8 dpa (Figures 6B–6I and S6). At 2 dpa, we could not distinguish BMPRi-treated from control fish, but by 4 dpa BMP inhibition produced a pronounced decrease in the extent and levels of *sp7:EGFP* expression (Figure S6). Paradoxically, by 8 dpa of BMPRi treatment, *sp7:EGFP* expression became relatively high, possibly indicating the activation of feedback mechanisms (Figure S6). Regardless, long-term inhibition of the BMP pathway (0–8 dpa) reduced bone formation (Figures 6B–6I) with BMPRi-treated fish lacking mature lepidotrichia including the joints between ray segments but maintaining regeneration of epidermis and blood vessels at 4 dpa (Figures S7E–S7H). Von Kossa staining revealed that fins from BMPRi-treated fish still formed a blastema but failed to produce mineralized bone (Figures S7I–S7L), consistent with a reduced expression domain of *col10a1,* a marker of differentiating osteoblasts (Smith et al., 2006) (Figures S7M and S7N). Importantly, pSmad1/5/8 levels were greatly diminished by chronic BMPRi treatment (Figures S7O and S7P), demonstrating drug efficacy. The bone-specific phenotype induced by BMPRi exposure contrasts those observed with IWP-2 or Wnt-C59, which largely blocked blastema outgrowth, and is consistent with the observation that BMP activity is primarily located in differentiating osteoblasts.

To determine BMP roles during ongoing regeneration, we treated animals with BMPRi from 48 to 72 hpa, which blocked Smad1/5/8 phosphorylation, increased Runx2$^+$ cells near the amputation site (Figures 6J–6O), and decreased sp7 expression (Figures 6P and 6R). We scored osteoblast subtypes in DMSO versus BMPRi-treated animals (Figure 6T), confirming BMPRi treatment increased Runx2$^+$ cells ($p < 0.002$) and decreased the Runx2$^+$/sp7$^+$ and the sp7$^+$ populations ($p < 0.007$ for the Runx2$^+$/sp7$^+$ cells and $p < 0.02$ for the sp7$^+$ population). EdU incorporation (Figures 6Q and 6S, quantified in Figure S7Q) and TUNEL staining (Figures S5Q–S5V) revealed osteoblast proliferation and cell death, respectively, were unaffected by BMPRi treatment. These results argue against a role for BMP in maintaining Runx2 expression (Smith et al., 2006) and rather demonstrate that BMP specifically drives osteoblast differentiation by promoting the transition from Runx2$^+$/sp7$^-$ cells to sp7$^+$ cells.

BMP Negatively Modulates Wnt/β-Catenin Activity in Regenerating Osteoblasts

Because inhibiting BMP activity expanded the Runx2$^+$ population, we hypothesized that BMP signaling in proximally located osteoblasts quenches Wnt/β-catenin activity. Indeed, stained fin sections from 48 to 72 hpa BMPRi-treated fish exhibited a dramatic increase of nuclear-localized β-catenin throughout an expanded population of Runx2$^+$ preosteoblasts (Figures 7A–7E and 7G–7K). The domain of *axin2* expression also was expanded proximally in BMPRi-exposed fish, further indicating that BMP functions in proximal osteoblasts to restrain Wnt activity to the distal-most progenitor pool (Figures 7F and 7L).

Cultured primary caudal fin osteoblasts became uniformly pSmad1/5/8$^+$ without the addition of exogenous BMP, suggesting cell autonomous BMP production and signaling (Figures S7R–S7U). Among BMPs reported to be expressed in the regenerating fin (Smith et al., 2006), we detected robust expression of *bmp2b* in cultured osteoblasts by quantitative RT-PCR (qRT-PCR) (Figure S7V). We reconstituted BMP's negative regulation of Wnt/β-catenin by combined addition of BMPRi and recombinant Wnt3A to primary fin osteoblasts. Wnt3A induced partial nuclear accumulation of β-catenin (Figures 7M–7S), which was substantially enhanced by inhibiting BMP signaling using BMPRi (Figures 7P–7S, $p < 0.01$). These results are congruent with our in vivo observations that BMP signaling is active in osteoblasts where its roles include negatively regulating Runx2$^+$ preosteoblasts by countering Wnt signals.

Dkk proteins compete with Wnt ligands for binding to Fz/Lrp5/6 receptor complexes (Mao et al., 2001). We therefore hypothesized that BMPs could negatively regulate Wnt/β-catenin signaling by activating Dkk expression. Primary fin osteoblasts expressed *dkk1a*, *dkk1b*, *dkk2*, and *dkk3b*, among which *dkk1a*, *dkk1b*, and *dkk3b* expression were reduced upon BMPRi treatment, as was *sp7* (Figure 8A, $p < 0.05$). We next examined BMP-dependent expression of *dkk* genes in regenerating fins from fish treated with DMSO or BMPRi from 48 to 96 hpa. *Dkk1b*, *dkk2*, and *dkk3b* all were significantly downregulated in BMPRi-treated animals (Figure 8B, $p < 0.05$). Consistent with antibody staining and the in vitro results, *sp7* transcript levels were also reduced upon BMPRi treatment. *Dkk2* was downregulated by BMPRi in vivo but not in cultured cells, possibly reflecting nonosteoblast expression of *dkk2* in intact fins. Unique among Dkk proteins, Dkk3 is not thought to directly antagonize Wnt/receptor interactions (Mao et al., 2001). Nevertheless, *dkk3b* was enriched in regenerating osteoblasts, and its expression was reduced by a 24 hr BMPRi treatment from 48 to 72 hpa (Figures S8A and S8B).

The BMP-dependent expression of *dkk1b* and its ability to inhibit Wnt signaling in zebrafish (Shinya et al., 2000) and attenuate fin regeneration (Stoick-Cooper et al., 2007) suggested that Dkk1b may be sufficient to inhibit the Wnt-dependent Runx2$^+$ preosteoblast pool. We amputated fins from control and *Tg(hsp70l:dkk1b-GFP)w32* fins, subjected them to a heat shock

(I–P) Immunostaining for Runx2 (red) and β-catenin (green) on 24 hpa sections from fish exposed to DMSO (I–L) or Wnt inhibitor (M–P, 10 μM IWP-2 from 0 to 24 hpa). The yellow arrow indicates Runx2$^+$ cells with nuclear-localized β-catenin. Nuclei are stained blue. Scale bars represent 50 μm.

(Q and R) *twist2* in situ hybridization on fins from DMSO (Q) and IWP-2-treated (R, 10 μM from 48 to 72 hpa) fish harvested 72 hpa. The red arrows point to *twist2*-expressing osteoblasts.

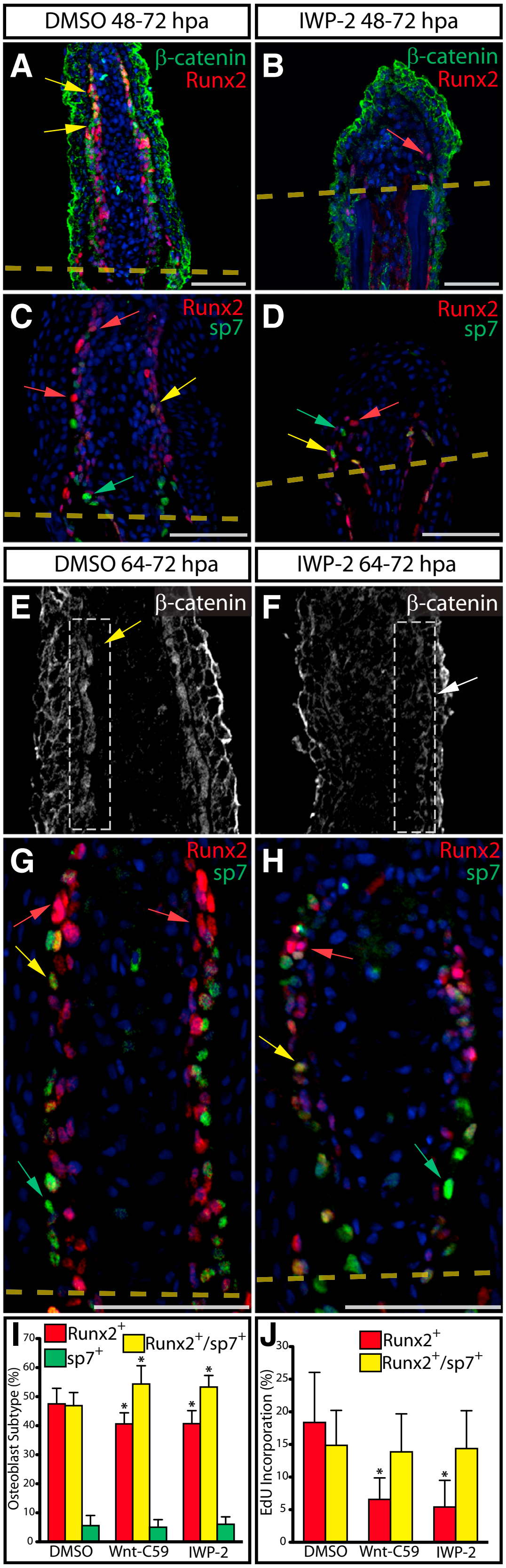

Figure 5. Wnt Is Required for Maintenance of Runx2⁺ Preosteoblasts during Fin Regeneration

(A–D) Runx2 (A–D, red), β-catenin (A and B, green), and sp7 (C and D, green) protein expression on sections from DMSO (A and C) and IWP-2-treated (B and D, 10 μM from 48 to 72 hpa) fish. In (A) and (B), yellow arrows indicate Runx2$^+$ cells with nuclear-localized β-catenin and the red arrow points to a Runx2$^+$ cell lacking nuclear β-catenin. In (C) and (D), red arrows show Runx2$^+$ cells, yellow arrows point to Runx2$^+$/sp7$^+$ cells, and green arrows mark sp7$^+$ cells.

(E and F) β-catenin localization at 72 hpa in DMSO (E) and IWP-2-treated fish (F, 10 μM at 64–72 hpa). The osteoblast-populated domain of the blastema is bounded by a dashed white box, and the white arrows point to osteoblasts with β-catenin expression.

(G and H) Runx2 (red) and sp7 (green) levels in DMSO (G) and IWP-2-treated fins (H, 10 μM at 64–72 hpa) harvested 72 hpa. Red, yellow, and green arrows indicate Runx2$^+$, Runx2$^+$/sp7$^+$, and sp7$^+$ cells, respectively. Nuclei are in blue. Scale bars represent 50 μm.

(I) Osteoblast subtype percentile representation on matched fin sections (n > 6 rays collected from three animals and representing >600 osteoblasts for each treatment) harvested 72 hpa following DMSO (64–72 hpa), Wnt-C59 (100 nM, 64–72 hpa), or IWP-2 (10 μM, 64–72 hpa) exposure. Asterisks indicate significant differences relative to DMSO-treated fish (all $p < 0.02$, one-tailed Student's t tests).

(J) Percentage of Runx2$^+$ and Runx2$^+$/sp7$^+$ osteoblasts in the same sections scored in (I) that had incorporated EdU. Asterisks indicate a significant decrease ($p < 0.003$, one tailed Student's t tests).

regimen from 48 to 72 hpa, and examined β-catenin localization and Runx2 expression. Dkk1b induction decreased nuclear-localized β-catenin and produced a pronounced deficiency in Runx2$^+$ preosteoblasts (Figures 8C–8H), consistent with Dkk1b being a potent BMP-dependent inhibitor of osteoblast progenitor renewal.

The division of regenerating fin osteoblasts into distal Wnt active and proximal BMP active populations suggests the pathway's respective ligands would be similarly distributed. Guided by earlier studies (Poss et al., 2000; Smith et al., 2006; Stoick-Cooper et al., 2007), we determined the cell specific expression patterns of *bmp2b*, *wnt5a*, *wnt5b*, and *wnt10a* transcripts on 72 hpa fin sections. In situ hybridization revealed that *bmp2b* mRNA was produced by differentiating proximal osteoblasts (Figure S8C). In contrast, expression of *wnt5a* (Figure S8D) and *wnt5b* (Figure S8E) was concentrated toward the distal end of the regenerating fin, in both blastema mesenchymal cells and basal epidermis. Although more weakly expressed, *wnt10a* was also present in distal and lateral regions of the blastema in or adjacent to osteoblasts (Figure S8F). To conclusively identify the cellular sources of *wnt5a* and *bmp2b* relative to osteoblast lineage cells, we performed double immunostaining/in situ hybridization studies. *Wnt5a* expression was expressed in mesenchymal cells occupying the extreme distal blastema bordered laterally by Runx2$^+$ preosteoblasts. Minimal *wnt5a* was detected in Runx2$^+$ preosteoblasts or blastema cells adjacent to maturing sp7-expressing osteoblasts (Figures 8I–8L). *Bmp2b* was expressed in maturing sp7$^+$ osteoblasts, but not Runx2$^+$ distal preosteoblasts (Figures 8M–8P). Based on their spatial separation, Wnt5a/5b and Bmp2b are attractive candidate ligands to simultaneously promote the opposing activities of Wnt-dependent preosteoblast maintenance and BMP-dependent osteoblast differentiation, respectively.

Cell PRESS
Open ACCESS

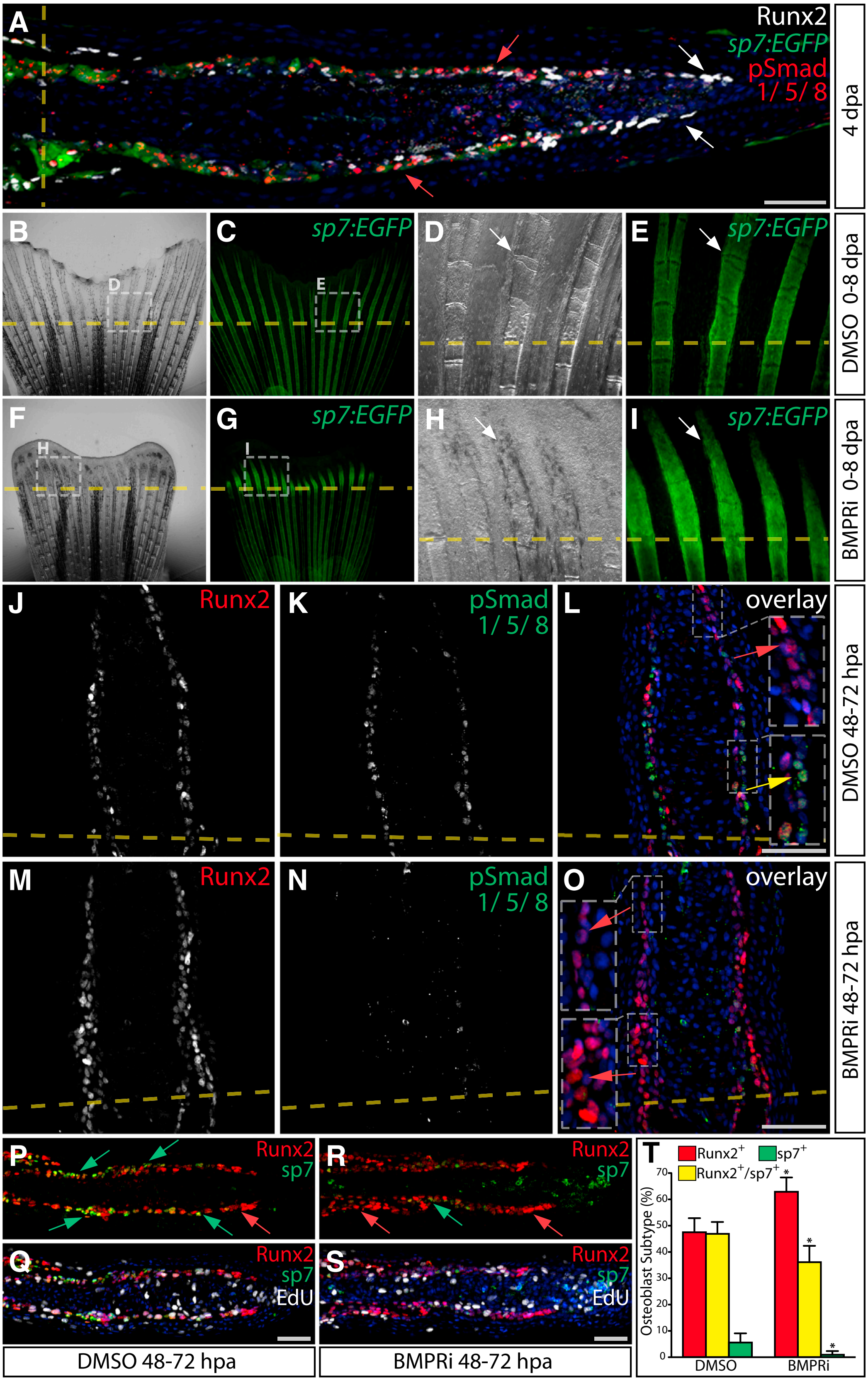

(legend on next page)

DISCUSSION

Bone regeneration in zebrafish fins requires regulatory mechanisms to (1) generate dedifferentiated progenitors from mature osteoblasts, (2) maintain this preosteoblast population until regeneration is complete, and (3) spatially and temporally restrict redifferentiation to a subset of preosteoblasts that progressively reform lost bone. The latter two processes are opposing activities; a fine balance between progenitor expansion and terminal differentiation must be maintained to sustain bone regeneration over 2 or more weeks. Our studies suggest a model for bone regeneration in the fin (Figure 8Q) whereby a Wnt-dependent EMT of epithelial osteoblasts populates the newly formed blastema with dedifferentiated Runx2^{+} preosteoblasts. Spatial segregation of two opposing pathways, Wnt/β-catenin and BMP, then balances growth and differentiation until regeneration is complete. Sustained high levels of Wnt in the distal blastema directly and continuously maintain a leading edge pool of Runx2^{+} mesenchymal preosteoblasts. Conversely, as more proximally located Runx2^{+} cells become physically distanced from the distal Wnt source, they upregulate *bmp2b* and activate autocrine BMP signaling. BMP promotes osteoblast differentiation by inducing *sp7* and *dkk1b* expression to feedback inhibit Wnt activity and prevent unproductive overexpansion of the progenitor pool. In agreement, *dkk1b* promoter elements drive gene expression exclusively in differentiating osteoblasts near the amputation site (Kang et al., 2013). As osteoblast maturation ensues, sp7-expressing cells downregulate Runx2, re-epithelialize by reforming adherens junctions, and progressively extend new mineralized bone (Figure 8Q).

Specific Roles for Wnt and BMP in the Osteoblast Lineage during Regeneration

Our examination of canonical Wnt activity shows that osteoblasts are a major Wnt-responsive cell type in the regenerating fin. Rapid changes in preosteoblast subtypes and their proliferation with short-term Wnt inhibitor exposure and the sufficiency of recombinant Wnt to promote osteoblast proliferation demonstrate canonical Wnt directly affects preosteoblasts. The requirement for Wnt in Runx2^{+} preosteoblast proliferation during fin regeneration is consistent with known functions for the Wnt/β-catenin pathway in maintaining progenitor cells in diverse biological settings (Clevers and Nusse, 2012) and regulating bone development in mice (Day et al., 2005; Glass et al., 2005; Gong et al., 2001; Hill et al., 2005; Hu et al., 2005; Rodda and McMahon, 2006). Precisely how Wnt/β-catenin signaling supports the preosteoblast state requires further study, but it is likely that Wnt/β-catenin's effects on transcription favor the silencing of genes required for differentiation and activation of genes associated with "stemness" (Clevers and Nusse, 2012).

We also observed *axin2* and *TOP:GFP* expression, as well as modest nuclear β-catenin in nonosteoblast distal blastema cells, indicating they, like preosteoblasts, are Wnt responsive. A role for Wnt signaling outside of osteoblasts is supported by our long-term Wnt inhibitor and Dkk1b overexpression experiments that produce a near complete arrest of fin regeneration. Therefore, akin to its role in preosteoblasts, canonical Wnt signaling may maintain these distal-most blastema cells, which then act as a signaling niche/center that orchestrates the overall fin regeneration process. This concept is described in detail in the accompanying paper from Wehner et al. (2014) in this issue of *Cell Reports*. W*nt5a*, *wnt5b*, and *wnt10a* are expressed in distal regenerating fin tissue (Poss et al., 2000; Stoick-Cooper et al., 2007), results we confirmed and expanded upon by demonstrating that *wnt5a* is expressed in distal blastema cells adjacent to Runx2^{+} preosteoblasts. Therefore, distal blastema cells may be a self-sustaining source of a Wnt morphogen gradient that both directly and indirectly regulates osteoblasts and other cell types of the regenerating fin.

Our observations show that BMP signaling likely is dedicated to differentiating osteoblasts in regenerating fins. Antibody staining localizes pSmad1/5/8 to proximal maturing osteoblasts and not distal Runx2^{+} preosteoblasts. Further, BMP receptor function is required for normal sp7 expression and bone formation, but not to maintain distal Runx2^{+} cells. Intriguingly, although distal Runx2^{+} cells with robust nuclear β-catenin reside near a field of *bmp* expression (Smith et al., 2006), they and other distal cells are pSmad1/5/8 negative and therefore likely refractory to BMP signals. One possible explanation is feedback inhibition of BMP in the distal blastema by Wnt-induced BMP antagonists. Ectopic expression of Chordin, a secreted BMP inhibitor, blocks fin regeneration (Smith et al., 2006), although it is unknown whether endogenous Chordin, or other BMP inhibitors, quench BMP signals in the context of fin regeneration. We find that cultured fin osteoblasts strongly express *bmp2b* and accordingly become pSmad1/5/8^{+}. Further, *bmp2b* expression is

Figure 6. BMP/Smad Signaling Promotes Osteoblast Differentiation and sp7 Expression during Bone Regeneration

(A) Runx2 (white), *sp7:EGFP* (green) and pSmad1/5/8 (red) levels on sections from a *Tg(sp7:EGFP)* fish 4 dpa. White arrows point to Runx2^{+}/pSmad^{-} preosteoblasts. Red arrows indicate sp7^{+}/pSmad1/5/8^{+} osteoblasts. The amputation site is marked with a dashed yellow line.

(B–I) Whole-mount images of regenerating fins of *Tg(sp7:EGFP)* fish at 8 dpa after DMSO (B–E) or BMPRi exposure (F–I, 5 μM from 0 to 8 dpa). Rotterman contrast (B, D, F, and H) and epifluorescence images (C, E, G, and I) to visualize *sp7:EGFP* expression (green) are shown. White arrows indicate osteoblasts. Representative images from one of three fish in each of the control and BMPRi groups are shown at low and high magnification.

(J–O) Antibody-stained fin sections showing Runx2 (red) and pSmad1/5/8 (green) levels in fish 72 hpa exposed to DMSO (J–L) or BMPRi (M–O, 5 μM BMPRi at 48–72 hpa). Red arrows indicate Runx2^{+}/pSmad^{-} cells, and the yellow arrow points to a Runx2^{low}/pSmad1/5/8^{+} cell. Insets show magnified boxed regions.

(P–S) EdU (white) incorporation in Runx2- (red) and sp7- (green) expressing cells in fin sections from DMSO (P and Q) and BMPRi-exposed animals (R and S, 5 μM at 48–72 hpa). Red arrows indicate Runx2^{+} cells and green arrows point to sp7^{+} cells. Overlay panels show Hoechst-stained nuclei in blue. Scale bars represent 50 μm.

(T) The percentage of Runx2^{+} (red bars), Runx2^{+}/sp7^{+} (yellow bars), and sp7^{+} cells (green bars) in fin sections from DMSO (48–72 hpa) versus BMPRi-treated (5 μM, 48–72 hpa) zebrafish. Four rays from three BMPRi-exposed animals containing a combined >400 osteoblasts were scored. The DMSO sample data are shared with the experiment shown in Figure 5I. Error bars are one SD, and significant p values are indicated with an asterisk ($p < 0.002$ for increased Runx2^{+} cells; $p < 0.007$ for a decrease in Runx2^{+}/sp7^{+} cells; and $p < 0.02$ for a decreased sp7^{+} population, Student's t tests).

Cell PRESS
Open ACCESS

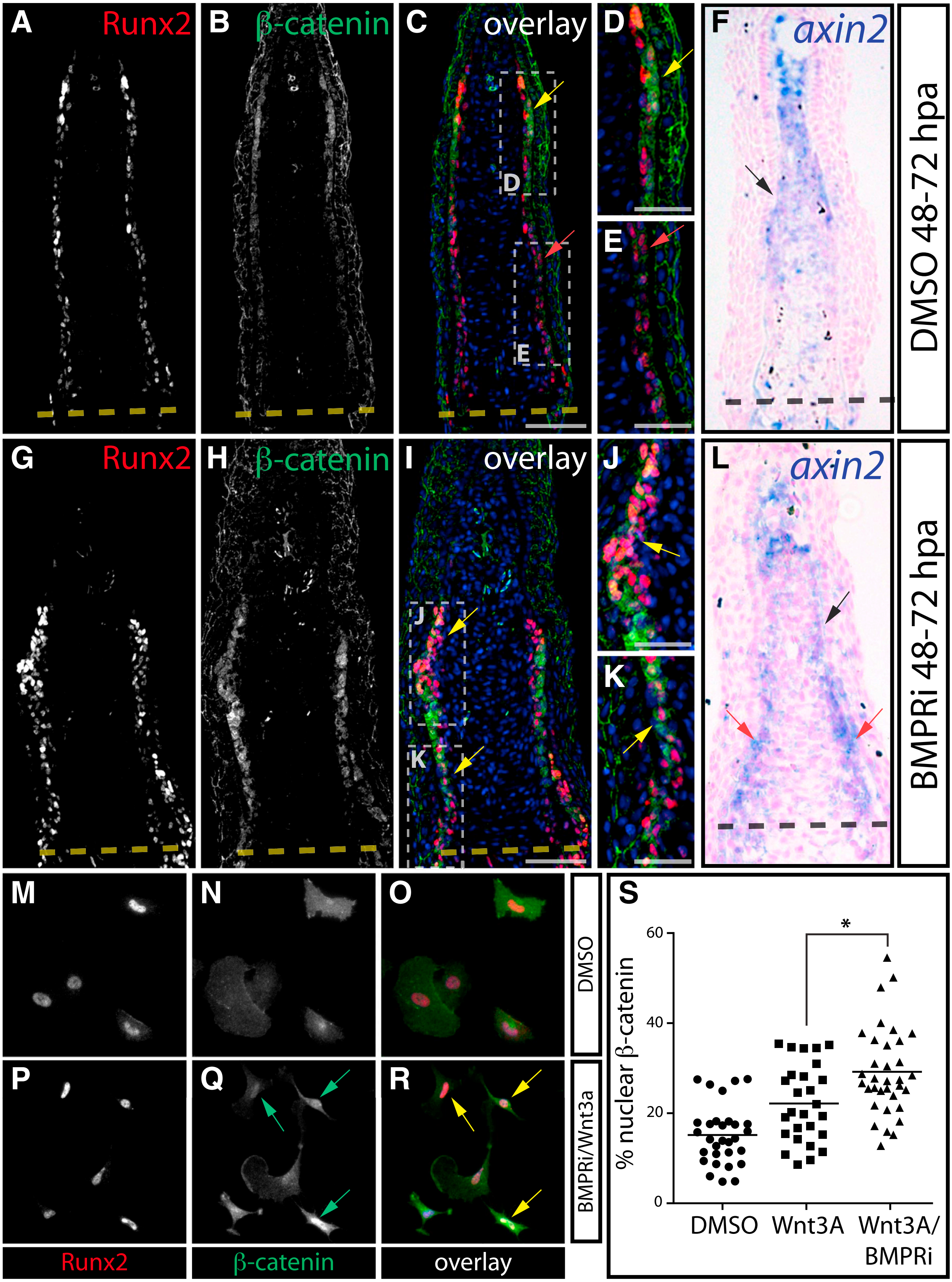

(legend on next page)

concentrated in differentiating sp7+ osteoblasts in vivo, as suggested by an earlier study (Smith et al., 2006). Direct downstream targets of BMP/Smad during bone regeneration could include both *sp7* and *dkk* genes, such as *dkk1b*. Our model does not resolve if other signaling pathways required for fin ray regeneration, including fibroblast growth factor (FGF) and Hedgehog (reviewed in Gemberling et al., 2013), interface with Wnt/β-catenin and BMPs in osteoblasts or have secondary effects on regenerating bone.

Opposing Activities of Wnt and BMP Establish Self-Renewing Tissues and Organs

Self-renewing tissues, including intestinal epithelium, stomach lining, interfollicular epidermis, sebaceous glands, and hair follicles of the skin, contain stem cells that produce cells whose differentiation states are both temporally and spatially arranged (Barker et al., 2010). The logic of how these hierarchical progenitor cell systems are maintained is remarkably similar to what we describe during fin bone regeneration with Wnt/BMP mutual antagonism being a common theme (Kandyba et al., 2013; Plikus et al., 2008). Mathematical models predict an obligatory role for negative feedback loops to balance stem cell renewal and differentiation (Lander et al., 2009). Therefore, an equilibrium between Wnt and BMP output may be a common logic component of regulatory networks that establish a precise balance between cell plasticity and differentiation.

Implications for Restorative Bone Therapies

Our interpretation of bone regenration in the zebrafish fin suggests that the signaling networks that control regenerative osteogenesis are evolutionarily conserved, relatively simple, and largely lineage intrinsic. Human bone may retain the competency to robustly regenerate if inherent networks could be somehow activated and/or augmented. Currently, recombinant BMPs are the only biologic factors approved for use in bone repair therapies; however, whereas BMPs can promote ossification, their clinical efficacy is controversial (Garrison et al., 2010). A potential explanation for this can be inferred from our results and those of others that show that BMPs downregulate Wnt/β-catenin signaling in bone (Minear et al., 2010a). New therapeutic approaches can be envisioned that exploit the features of regenerative osteogenesis that we have uncovered. For instance, first enhancing Wnt/β-catenin activity using small molecules or Wnt proteins soon after injury may expand endogenous preosteoblasts, an idea supported by enhanced healing of damaged bone in mice treated with recombinant Wnts (Minear et al., 2010b). Then, later stages of bone healing may benefit from BMP treatment to enhance osteoblast redifferentiation and mineralization. More broadly, our observations encourage approaches aimed at coaxing human cells to mimic those of animals, like zebrafish, that have remarkably robust regenerative abilities.

EXPERIMENTAL PROCEDURES

Zebrafish

Wild-type AB, *Tg(sp7:EGFP)b1212* (DeLaurier et al., 2010), *Tg(hsp70l:dkk1b-GFP)w32* (Stoick-Cooper et al., 2007), *Tg(dusp6:Cre-ERT2,myl7:ECFP) b1230* (Stewart and Stankunas, 2012), *Tg(TOP:GFP)w25* (Dorsky et al., 2002), and *Tg(Xla.Eef1a1-actb2:LOXP-LOX5171-FRT-F3-EGFP,mCherry) vu295a* (Boniface et al., 2009) lines were maintained according to University of Oregon institutional guidelines. Regeneration studies were performed at 28°C –29°C as described previously (Stewart and Stankunas, 2012).

Immunostaining

Sections were rehydrated and antigen retrieval was performed for 10 min in a pressure cooker in retrieval buffer (1 mM EDTA [pH 8], 0.1% Tween 20). Antibodies were diluted in PBST containing 10% nonfat dry milk and applied to slides overnight at 4°C, followed by staining with Alexa-conjugated secondary antibodies (Invitrogen) and Hoechst nuclear staining. Antibody staining details are provided in the Supplemental Experimental Procedures.

Drug Treatments

Wnt pathway inhibitor IWP-2 and the BMPR inhibitor, LDN193189, referred to in the text and figures as BMPRi, were purchased from StemRD; Wnt-C59 was from Biovision; all were dissolved in DMSO. At the indicated times after fin amputation, IWP-2, Wnt-C59, BMPRi, or DMSO was added to fish water (one animal per 200 ml of water) at 10 μM, 100 nM, or 5 μM, respectively. For each drug treatment (n = 3), cohorts of three or four animals were used, each was analyzed at the completion of the study, and images shown are representative examples of each cohort.

In Situ Hybridization

For combination fluorescent in situ hybridization immunostaining, sections on slides were sequentially antigen retrieval treated, hybridized with DIG-labeled probes, stained with α-DIG peroxidase-conjugated antibody (Roche), developed using the TSA system (PerkinElmer), and finally immunostained. Protocol details and probe synthesis are described in the Supplemental Experimental Procedures.

Heat Shock Studies, EdU Labeling, Osteoblast Cell Culture, Quantitative RT-PCR, Mosaic Analysis, TUNEL Staining, and Histology

These methods are presented in the Supplemental Experimental Procedures.

Statistical Analysis

Statistically significant differences between osteoblast subtypes were determined by scoring their fractional representation on comparable immunostained sections (for Figures 1 and 5, >600 osteoblasts from more than six

Figure 7. BMP Negatively Regulates Wnt/β-Catenin Signaling

(A–L) Immunostaining (A–E and G–K) with Runx2 (red) and β-catenin (green) antibodies and *axin2* in situ hybridization (F and L) on sectioned fins from DMSO (A–F) and BMPRi-treated fish (G–L, 5 μM at 48–72 hpa). Yellow arrows highlight Runx2+ cells with nuclear-localized β-catenin, and red arrows point to osteoblasts with membrane-localized β-catenin. Hoechst-stained nuclei are in blue. For (F) and (L), *axin2* expression (blue) in distal osteoblasts is indicated by black arrows. In (L), red arrows point to proximal *axin2*+ osteoblasts. Scale bars represent 50 μm (A–C and G–I) and 25 μm (D, E, J, and K).

(M–R) Runx2 expression (red) and β-catenin localization (green) in antibody-stained primary zebrafish fin osteoblasts grown in media containing DMSO (M–O) or BMPRi + Wnt3a (P–R, 300 nM and 40 ng/ml, respectively) for 24 hr beginning at 4 days postisolation. Green arrows point to cells with nuclear β-catenin localization, and yellow arrows indicate Runx2+ cells displaying nuclear β-catenin.

(S) Plots showing nuclear-localized β-catenin versus total cell β-catenin in Runx2+ cells in individual, randomly selected cultured fin osteoblasts following DMSO, Wnt3 (40 ng/ml), or BMPRi + Wnt3a (300 nM and 40 ng/ml, respectively) exposure. A line marks the mean of each group. The Wnt3a + BMPRi population displayed significantly increased nuclear β-catenin relative to the Wnt3A-alone-treated group as determined by one-way ANOVA and a post hoc Tukey's test ($p > 0.01$, indicated by an asterisk).

Cell PRESS
Open ACCESS

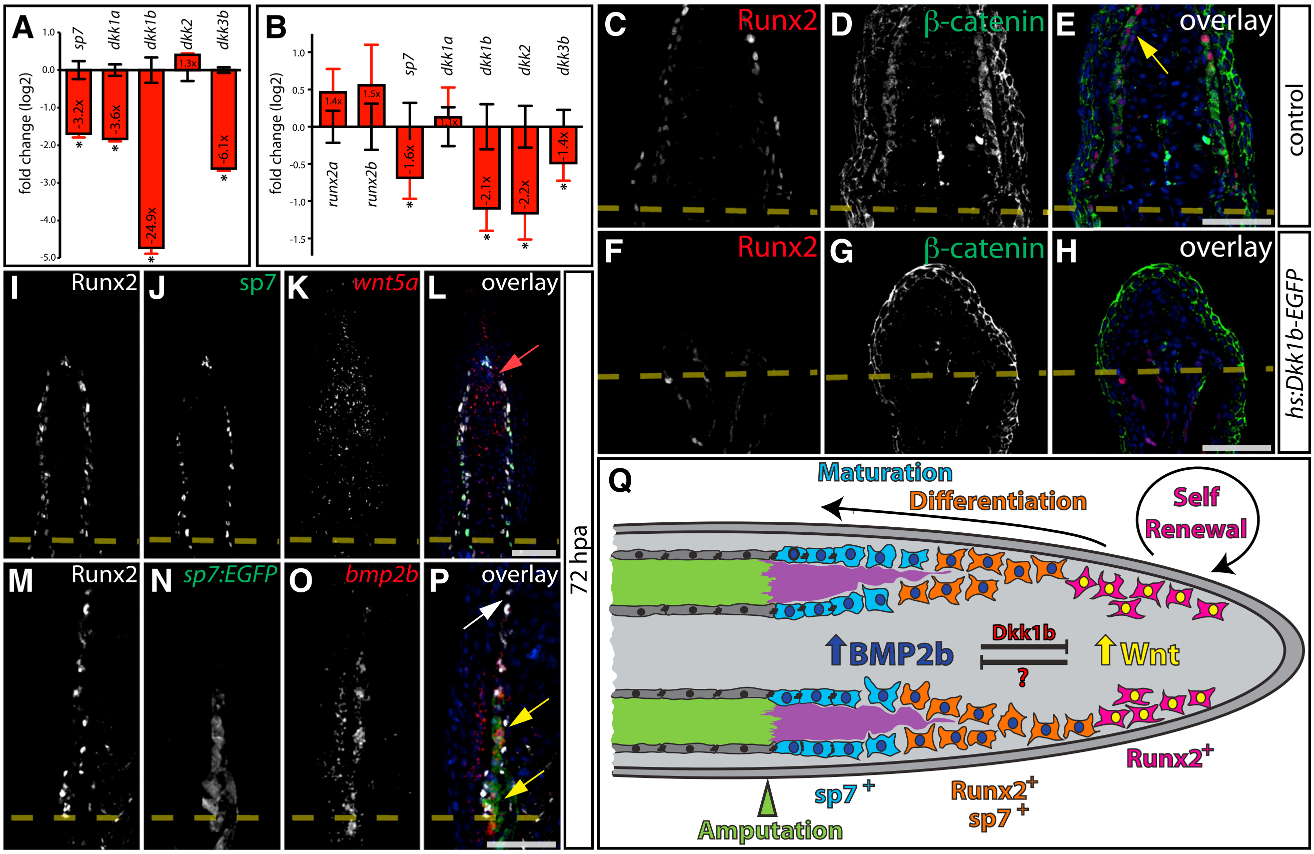

Figure 8. Spatially Distributed Gene Expression Combined with Negative Feedback Maintains Regenerative Osteogenesis

(A) Quantitative RT-PCR analysis of *sp7* and *dkk* genes in primary fin osteoblasts after 24 hr of 300 nM BMPRi (red bars and error bars) relative to control DMSO-treated cells (variation shown with black error bars). For each primer pair, the normalized average fold change in transcript levels upon BMPRi treatment is plotted on a $\log_2$ scale with DMSO samples averaged to $\log_2 0 = 1$. Each group includes three independent cultures. Error bars are one SD. Statistically significant differences are indicated with an asterisk ($p < 0.05$, two-tailed Student's t tests). A representative example of three independent experiments is shown.

(B) Relative gene expression of osteogenic factors and *dkk* genes at 96 hpa following DMSO (black error bars) or BMPRi exposure (red bars, 5 μM from 48 to 96 hpa). Mean normalized levels of the indicated transcripts from four fins per treatment group are shown on a $\log_2$ scale. Error bars represent one SD, and asterisks mark differentially expressed genes ($p < 0.05$, two-tailed Student's t tests).

(C–H) Antibody staining for Runx2 (red) and β-catenin (green) on sectioned fins from control (C–E, two heat treatments between 48 and 72 hpa) and *Tg(hsp70l:dkk1b-GFP)* fish (F–H, two heat treatments between 48 and 72 hpa) harvested 72 hpa. The yellow arrow indicates Runx2$^+$ cells with nuclear β-catenin.

(I–L) Immunostaining of a 72 hpa fin cryosection with Runx2 (I, white) and sp7 (J, green) antibodies and simultaneous *wnt5a* mRNA in situ hybridization (K, red), overlaid in (L). The red arrow shows *wnt5a* expression in distal mesenchymal cells.

(M–P) A fin cryosection from a 72 hpa *Tg(sp7:EGFP)* fish showing antibody staining for Runx2 (M, white) and EGFP (N, green) and *bmp2b* mRNA in situ hybridization (O, red), overlaid in (P). The white arrow points to Runx2$^+$/*bmp2b*$^-$ cells, and yellow arrows indicate overlapping expression of *sp7:EGFP* and *bmp2b*. Hoechst-stained nuclei are in blue in overlay panels. Scale bars represent 50 μm.

(Q) A signaling network model for osteogenesis during fin regeneration. Wnt acts distally to maintain a pool of Runx2$^+$ osteoblast progenitor cells, whereas Bmp2b-initiated signaling in progenitor-derived cells both promotes sp7-associated differentiation and constrains Wnt activity by inducing Dkk1b.

rays compiled from at least three fish; for Figure 6, >400 osteoblasts from more than four rays collected from at least three animals). One-tailed Student's t tests compared the means of each population's percentage of total osteoblasts across individual rays. Fisher's exact tests were used to determine significant differences in the proportion of EdU$^+$ cells between osteoblast subtypes, combining osteoblasts scored in 12 rays from at least four fish (>1,100 cells total). To assess changes in EdU incorporation between drug-treated fish, comparable stained sections were scored for the fraction of EdU incorporating cells of each osteoblast subtype. Student's t tests assessed differences between drug-treated groups using the means of each subtype's percentage of EdU$^+$ incorporating cells across individual rays. For nuclear β-catenin quantitation in cultured osteoblasts, one-way ANOVA and post hoc Tukey's tests were used to determine significant differences in the ratio of nuclear to total β-catenin in individual cells across and between treatments. To determine differentially expressed gene in qRT-PCR studies, two-tailed Student's t tests used raw ΔCts from three or four independent control and experimental samples.

SUPPLEMENTAL INFORMATION

Supplemental Information includes Supplemental Experimental Procedures and eight figures and can be found with this article online at http://dx.doi.org/10.1016/j.celrep.2014.01.010.

ACKNOWLEDGMENTS

We thank the University of Oregon Zebrafish Facility for fish care; the University Oregon zebrafish community for support; A. Delaurier and C. Kimmel for fish lines and *sp7* plasmid; W. Chen for fish lines; M. Akimenko for *runx2a*, *col10a1*, and *bmp2b* plasmids; M. Westerfield for *axin2* plasmid; C. Camarillo for technical assistance; and the K.S. lab for their input. The research was funded by an Oregon Scientist Development Award from the Oregon Medical Research Foundation (S.S.), NIH training grants 5T32HD007348 and 2T32GM007413 (B.E.A.), and the NIH/NHLBI (5R00HL087598) and the University of Oregon (K.S.).

Received: April 12, 2013
Revised: August 29, 2013
Accepted: January 7, 2014
Published: January 30, 2014

REFERENCES

Abzhanov, A., Rodda, S.J., McMahon, A.P., and Tabin, C.J. (2007). Regulation of skeletogenic differentiation in cranial dermal bone. Development *134*, 3133–3144.

Bandyopadhyay, A., Tsuji, K., Cox, K., Harfe, B.D., Rosen, V., and Tabin, C.J. (2006). Genetic analysis of the roles of BMP2, BMP4, and BMP7 in limb patterning and skeletogenesis. PLoS Genet. *2*, e216.

Barker, N., Bartfeld, S., and Clevers, H. (2010). Tissue-resident adult stem cell populations of rapidly self-renewing organs. Cell Stem Cell *7*, 656–670.

Bialek, P., Kern, B., Yang, X., Schrock, M., Sosic, D., Hong, N., Wu, H., Yu, K., Ornitz, D.M., Olson, E.N., et al. (2004). A twist code determines the onset of osteoblast differentiation. Dev. Cell *6*, 423–435.

Boniface, E.J., Lu, J., Victoroff, T., Zhu, M., and Chen, W. (2009). FlEx-based transgenic reporter lines for visualization of Cre and Flp activity in live zebrafish. Genesis *47*, 484–491.

Chen, B., Dodge, M.E., Tang, W., Lu, J., Ma, Z., Fan, C.W., Wei, S., Hao, W., Kilgore, J., Williams, N.S., et al. (2009). Small molecule-mediated disruption of Wnt-dependent signaling in tissue regeneration and cancer. Nat. Chem. Biol. *5*, 100–107.

Clevers, H., and Nusse, R. (2012). Wnt/β-catenin signaling and disease. Cell *149*, 1192–1205.

Cuny, G.D., Yu, P.B., Laha, J.K., Xing, X., Liu, J.F., Lai, C.S., Deng, D.Y., Sachidanandan, C., Bloch, K.D., and Peterson, R.T. (2008). Structure-activity relationship study of bone morphogenetic protein (BMP) signaling inhibitors. Bioorg. Med. Chem. Lett. *18*, 4388–4392.

Day, T.F., Guo, X., Garrett-Beal, L., and Yang, Y. (2005). Wnt/beta-catenin signaling in mesenchymal progenitors controls osteoblast and chondrocyte differentiation during vertebrate skeletogenesis. Dev. Cell *8*, 739–750.

DeLaurier, A., Eames, B.F., Blanco-Sánchez, B., Peng, G., He, X., Swartz, M.E., Ullmann, B., Westerfield, M., and Kimmel, C.B. (2010). Zebrafish sp7:EGFP: a transgenic for studying otic vesicle formation, skeletogenesis, and bone regeneration. Genesis *48*, 505–511.

Dimitriou, R., Jones, E., McGonagle, D., and Giannoudis, P.V. (2011). Bone regeneration: current concepts and future directions. BMC Med. *9*, 66.

Dorsky, R.I., Sheldahl, L.C., and Moon, R.T. (2002). A transgenic Lef1/beta-catenin-dependent reporter is expressed in spatially restricted domains throughout zebrafish development. Dev. Biol. *241*, 229–237.

Ducy, P., Zhang, R., Geoffroy, V., Ridall, A.L., and Karsenty, G. (1997). Osf2/Cbfa1: a transcriptional activator of osteoblast differentiation. Cell *89*, 747–754.

Ferrari, S.L., Traianedes, K., Thorne, M., Lafage-Proust, M.H., Genever, P., Cecchini, M.G., Behar, V., Bisello, A., Chorev, M., Rosenblatt, M., and Suva, L.J. (2000). A role for N-cadherin in the development of the differentiated osteoblastic phenotype. J. Bone Miner. Res. *15*, 198–208.

Fulzele, K., Riddle, R.C., DiGirolamo, D.J., Cao, X., Wan, C., Chen, D., Faugere, M.C., Aja, S., Hussain, M.A., Brüning, J.C., and Clemens, T.L. (2010). Insulin receptor signaling in osteoblasts regulates postnatal bone acquisition and body composition. Cell *142*, 309–319.

Garrison, K.R., Shemilt, I., Donell, S., Ryder, J.J., Mugford, M., Harvey, I., Song, F., and Alt, V. (2010). Bone morphogenetic protein (BMP) for fracture healing in adults. Cochrane Database Syst. Rev., CD006950.

Gemberling, M., Bailey, T.J., Hyde, D.R., and Poss, K.D. (2013). The zebrafish as a model for complex tissue regeneration. Trends Genet. *29*, 611–620.

Glass, D.A., 2nd, Bialek, P., Ahn, J.D., Starbuck, M., Patel, M.S., Clevers, H., Taketo, M.M., Long, F., McMahon, A.P., Lang, R.A., and Karsenty, G. (2005). Canonical Wnt signaling in differentiated osteoblasts controls osteoclast differentiation. Dev. Cell *8*, 751–764.

Gong, Y., Slee, R.B., Fukai, N., Rawadi, G., Roman-Roman, S., Reginato, A.M., Wang, H., Cundy, T., Glorieux, F.H., Lev, D., et al.; Osteoporosis-Pseudoglioma Syndrome Collaborative Group (2001). LDL receptor-related protein 5 (LRP5) affects bone accrual and eye development. Cell *107*, 513–523.

Hill, T.P., Später, D., Taketo, M.M., Birchmeier, W., and Hartmann, C. (2005). Canonical Wnt/beta-catenin signaling prevents osteoblasts from differentiating into chondrocytes. Dev. Cell *8*, 727–738.

Hu, H., Hilton, M.J., Tu, X., Yu, K., Ornitz, D.M., and Long, F. (2005). Sequential roles of Hedgehog and Wnt signaling in osteoblast development. Development *132*, 49–60.

Izu, Y., Sun, M., Zwolanek, D., Veit, G., Williams, V., Cha, B., Jepsen, K.J., Koch, M., and Birk, D.E. (2011). Type XII collagen regulates osteoblast polarity and communication during bone formation. J. Cell Biol. *193*, 1115–1130.

Johnson, S.L., and Weston, J.A. (1995). Temperature-sensitive mutations that cause stage-specific defects in Zebrafish fin regeneration. Genetics *141*, 1583–1595.

Kandyba, E., Leung, Y., Chen, Y.B., Widelitz, R., Chuong, C.M., and Kobielak, K. (2013). Competitive balance of intrabulge BMP/Wnt signaling reveals a robust gene network ruling stem cell homeostasis and cyclic activation. Proc. Natl. Acad. Sci. USA *110*, 1351–1356.

Kang, J., Nachtrab, G., and Poss, K.D. (2013). Local Dkk1 crosstalk from breeding ornaments impedes regeneration of injured male zebrafish fins. Dev. Cell *27*, 19–31.

Kawakami, Y., Rodriguez Esteban, C., Raya, M., Kawakami, H., Martí, M., Dubova, I., and Izpisúa Belmonte, J.C. (2006). Wnt/beta-catenin signaling regulates vertebrate limb regeneration. Genes Dev. *20*, 3232–3237.

Knopf, F., Hammond, C., Chekuru, A., Kurth, T., Hans, S., Weber, C.W., Mahatma, G., Fisher, S., Brand, M., Schulte-Merker, S., and Weidinger, G. (2011). Bone regenerates via dedifferentiation of osteoblasts in the zebrafish fin. Dev. Cell *20*, 713–724.

Komori, T., Yagi, H., Nomura, S., Yamaguchi, A., Sasaki, K., Deguchi, K., Shimizu, Y., Bronson, R.T., Gao, Y.H., Inada, M., et al. (1997). Targeted disruption of Cbfa1 results in a complete lack of bone formation owing to maturational arrest of osteoblasts. Cell *89*, 755–764.

Lander, A.D., Gokoffski, K.K., Wan, F.Y., Nie, Q., and Calof, A.L. (2009). Cell lineages and the logic of proliferative control. PLoS Biol. *7*, e15.

Lim, J., and Thiery, J.P. (2012). Epithelial-mesenchymal transitions: insights from development. Development *139*, 3471–3486.

Long, F. (2012). Building strong bones: molecular regulation of the osteoblast lineage. Nat. Rev. Mol. Cell Biol. *13*, 27–38.

Mao, B., Wu, W., Li, Y., Hoppe, D., Stannek, P., Glinka, A., and Niehrs, C. (2001). LDL-receptor-related protein 6 is a receptor for Dickkopf proteins. Nature *411*, 321–325.

Minear, S., Leucht, P., Miller, S., and Helms, J.A. (2010a). rBMP represses Wnt signaling and influences skeletal progenitor cell fate specification during bone repair. J. Bone Miner. Res. *25*, 1196–1207.

Minear, S., Leucht, P., Jiang, J., Liu, B., Zeng, A., Fuerer, C., Nusse, R., and Helms, J.A. (2010b). Wnt proteins promote bone regeneration. Sci. Transl. Med. *2*, 29ra30.

Nakashima, K., Zhou, X., Kunkel, G., Zhang, Z., Deng, J.M., Behringer, R.R., and de Crombrugghe, B. (2002). The novel zinc finger-containing transcription

factor osterix is required for osteoblast differentiation and bone formation. Cell *108*, 17–29.

Otto, F., Thornell, A.P., Crompton, T., Denzel, A., Gilmour, K.C., Rosewell, I.R., Stamp, G.W., Beddington, R.S., Mundlos, S., Olsen, B.R., et al. (1997). Cbfa1, a candidate gene for cleidocranial dysplasia syndrome, is essential for osteoblast differentiation and bone development. Cell *89*, 765–771.

Plikus, M.V., Mayer, J.A., de la Cruz, D., Baker, R.E., Maini, P.K., Maxson, R., and Chuong, C.M. (2008). Cyclic dermal BMP signalling regulates stem cell activation during hair regeneration. Nature *451*, 340–344.

Poss, K.D., Shen, J., and Keating, M.T. (2000). Induction of lef1 during zebrafish fin regeneration. Dev. Dyn. *219*, 282–286.

Proffitt, K.D., Madan, B., Ke, Z., Pendharkar, V., Ding, L., Lee, M.A., Hannoush, R.N., and Virshup, D.M. (2013). Pharmacological inhibition of the Wnt acyltransferase PORCN prevents growth of WNT-driven mammary cancer. Cancer Res. *73*, 502–507.

Rodda, S.J., and McMahon, A.P. (2006). Distinct roles for Hedgehog and canonical Wnt signaling in specification, differentiation and maintenance of osteoblast progenitors. Development *133*, 3231–3244.

Shinya, M., Eschbach, C., Clark, M., Lehrach, H., and Furutani-Seiki, M. (2000). Zebrafish Dkk1, induced by the pre-MBT Wnt signaling, is secreted from the prechordal plate and patterns the anterior neural plate. Mech. Dev. *98*, 3–17.

Singh, S.P., Holdway, J.E., and Poss, K.D. (2012). Regeneration of amputated zebrafish fin rays from de novo osteoblasts. Dev. Cell *22*, 879–886.

Smith, A., Avaron, F., Guay, D., Padhi, B.K., and Akimenko, M.A. (2006). Inhibition of BMP signaling during zebrafish fin regeneration disrupts fin growth and scleroblasts differentiation and function. Dev. Biol. *299*, 438–454.

Sousa, S., Afonso, N., Bensimon-Brito, A., Fonseca, M., Simões, M., Leon, J., Roehl, H., Cancela, M.L., and Jacinto, A. (2011). Differentiated skeletal cells contribute to blastema formation during zebrafish fin regeneration. Development *138*, 3897–3905.

Stark, K., Vainio, S., Vassileva, G., and McMahon, A.P. (1994). Epithelial transformation of metanephric mesenchyme in the developing kidney regulated by Wnt-4. Nature *372*, 679–683.

Stewart, S., and Stankunas, K. (2012). Limited dedifferentiation provides replacement tissue during zebrafish fin regeneration. Dev. Biol. *365*, 339–349.

Stoick-Cooper, C.L., Weidinger, G., Riehle, K.J., Hubbert, C., Major, M.B., Fausto, N., and Moon, R.T. (2007). Distinct Wnt signaling pathways have opposing roles in appendage regeneration. Development *134*, 479–489.

Tran, T.H., Jarrell, A., Zentner, G.E., Welsh, A., Brownell, I., Scacheri, P.C., and Atit, R. (2010). Role of canonical Wnt signaling/ß-catenin via Dermo1 in cranial dermal cell development. Development *137*, 3973–3984.

Tu, S., and Johnson, S.L. (2011). Fate restriction in the growing and regenerating zebrafish fin. Dev. Cell *20*, 725–732.

Wehner, D., Cizelsky, W., Vasudevaro, M.D., Özhan, G., Haase, C., Kagermeier-Schenk, B., Röder, A., Dorsky, R.I., Moro, E., Argenton, F., et al. (2014). Wnt/β-catenin signaling defines organizing centers that orchestrate growth and differentiation of the regenerating zebrafish caudal fin. Cell Rep. *6*. Published online February 13, 2014. http://dx.doi.org/10.1016/j.celrep.2013.12.036.

SUPPLEMENTAL INFORMATION

Sequential and opposing activities of Wnt and BMP coordinate zebrafish bone regeneration

Scott Stewart, Alan W. Gomez, Benjamin E. Armstrong, Astra Henner, Kryn Stankunas

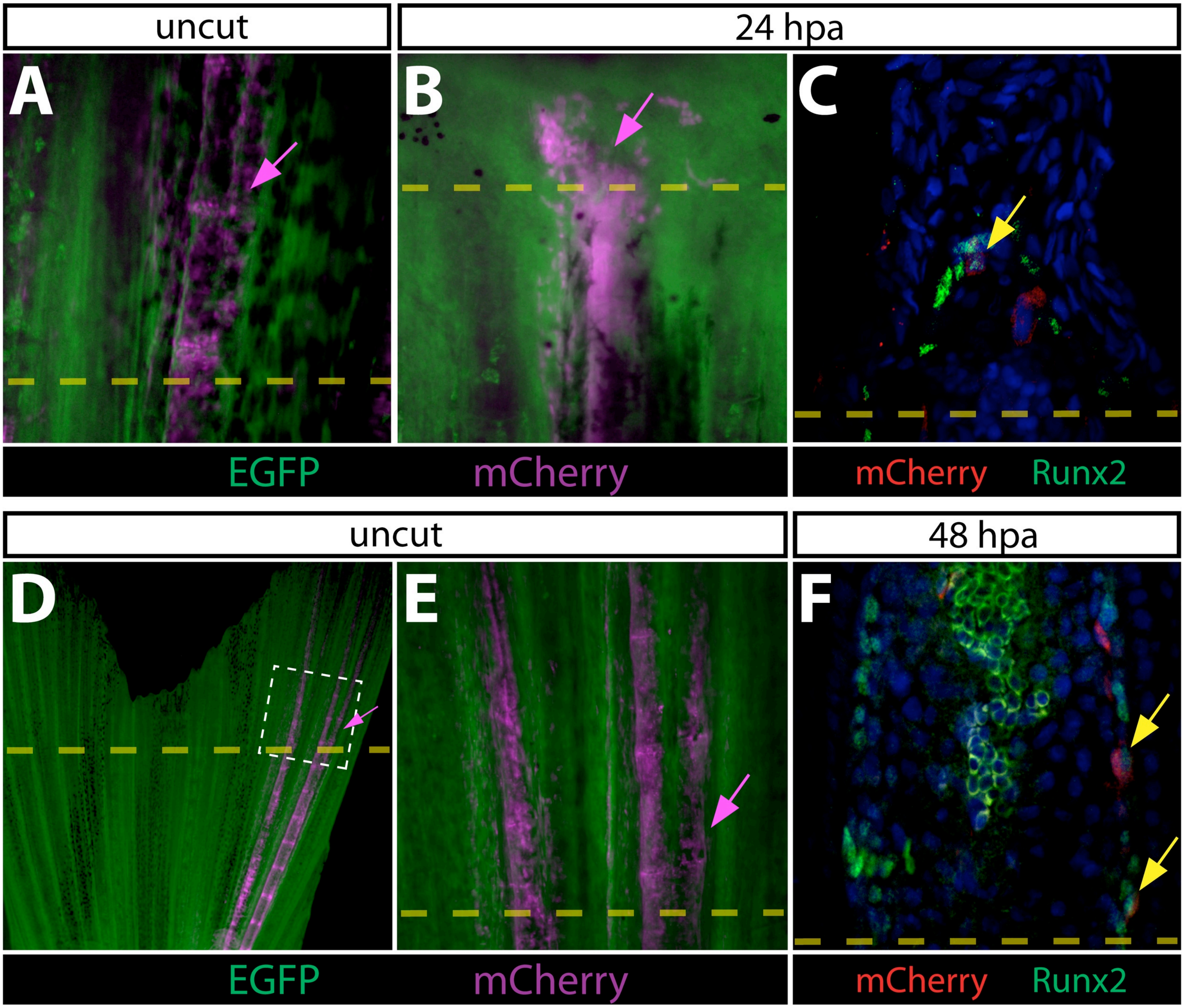

Figure S1, related to Figure 1

Figure S1, related to Figure 1. Pre-existing osteoblasts generate Runx2^{+} blastema cells.

(A-C) Whole-mount epifluorescence microscopy of a mosaic adult *Tg(Xla.ef1a1-actb2:LOXP-LOX5171-FRT-F3-EGFP,mCherry); Tg(dusp6:CreERT2,myl7:ECFP)* caudal fin mosaic displaying osteoblasts labeled by permanent mCherry expression (magenta) before amputation (A) and at 24 hpa (B). (C) Immunostaining of a section of this fin ray at 24 hpa showing Runx2 (green) and mCherry expression (red) in cells distal to the amputation site (dashed yellow line). Nuclei stained with Hoechst are shown in blue. **(D-F)** Another *Tg(Xla.ef1a1-actb2:LOXP-LOX5171-FRT-F3-EGFP,mCherry); Tg(dusp6:CreERT2,myl7:ECFP)* osteoblast mosaic visualized by whole-mount epifluorescence microscopy at 25x (D). The region in the dashed box is shown at 120x magnification (E). (F) Immunostaining of a section of this mosaic fin at 48 hpa showing Runx2 (green) and mCherry (red) co-expression. Nuclei are stained with Hoechst (blue). The dashed yellow line marks the amputation plane. Magenta arrows point to the osteoblasts expressing mCherry in mosaic lepidotrichia and yellow arrows indicate the Runx2^{+}/mCherry^{+} mosaic-derived cells.

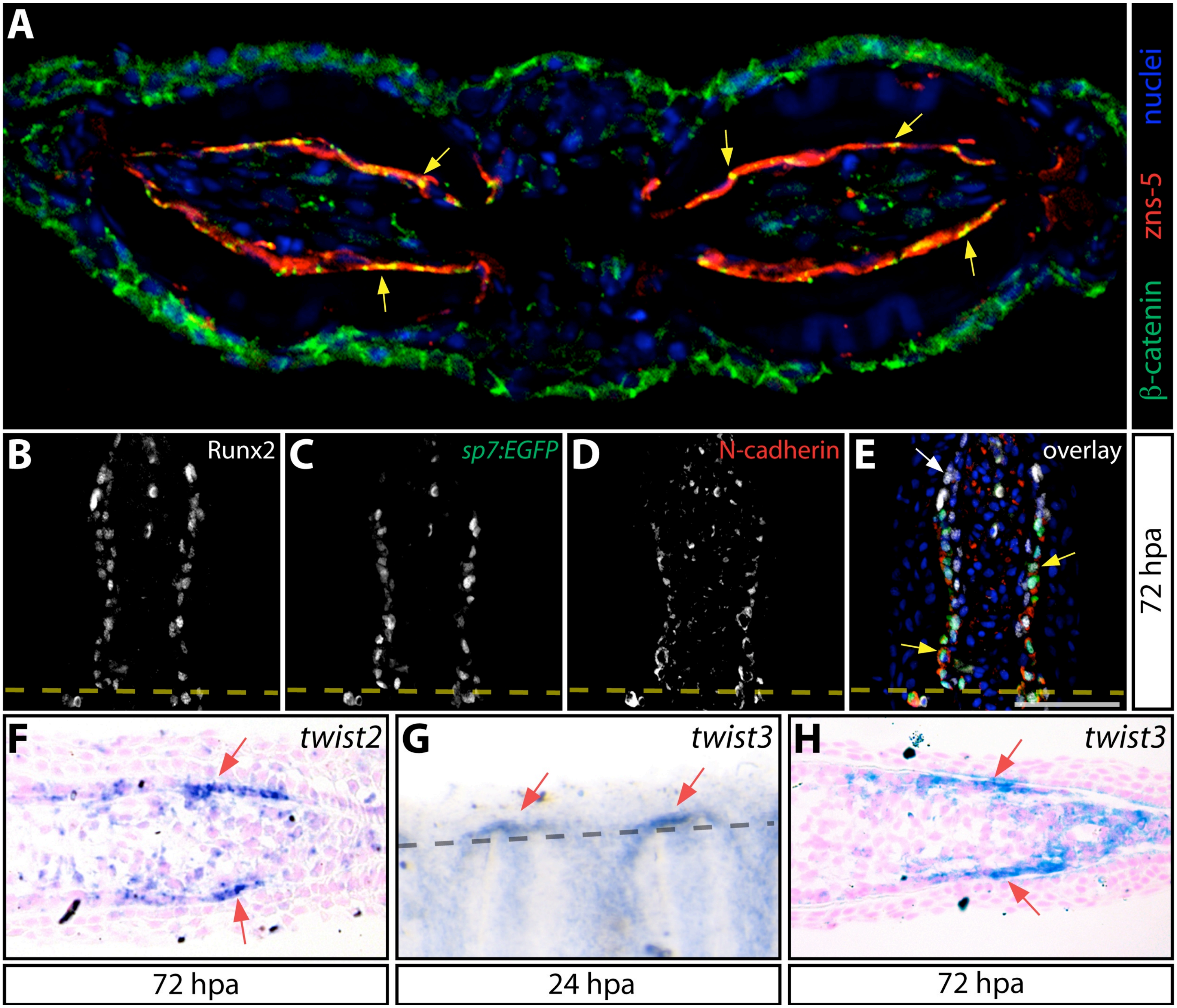

Figure S2, related to Figure 2

Figure S2, related to Figure 2. Dual epithelial/mesenchymal nature of osteoblasts during fin regeneration.

(A) Immunostaining for the osteoblast lineage marker zns-5 (red) and β-catenin (green) on a transverse section from a non-regenerating fin. Hoechst-stained nuclei are shown in blue. Yellow arrows point to adherens junctions between β-catenin/zns-5 labeled osteoblasts. **(B-E)** Expression of Runx2 (B, in white), *sp7:EGFP* (C, in green), and N-cadherin (D, in red) are shown in overlay (E) on 72 hpa fin sections. The white arrow points to distal Runx2$^+$ pre-osteoblasts lacking N-cadherin expression and yellow arrows indicate sp7$^+$ maturing osteoblasts that display membrane-localized N-cadherin. The yellow dashed line indicates the amputation plane and the scale bar represents 50 μm. **(F)** Expression of *twist2* mRNA (in blue) using in situ hybridization on 72 hpa fin paraffin sections. Red arrows point to distal pre-osteoblasts expressing *twist2*. **(G and H)** In situ hybridization to localize expression of *twist3* on whole-mount fins at 24 hpa. (G) and paraffin sections of 72 hpa fins (H). In (G), red arrows point to cells expressing *twist3* (in blue) distal to the amputation site (grey dashed arrow). In (H), red arrows point to distal pre-osteoblasts exhibiting *twist3* expression (in blue). Shown are representative images from experiments repeated > 3 times.

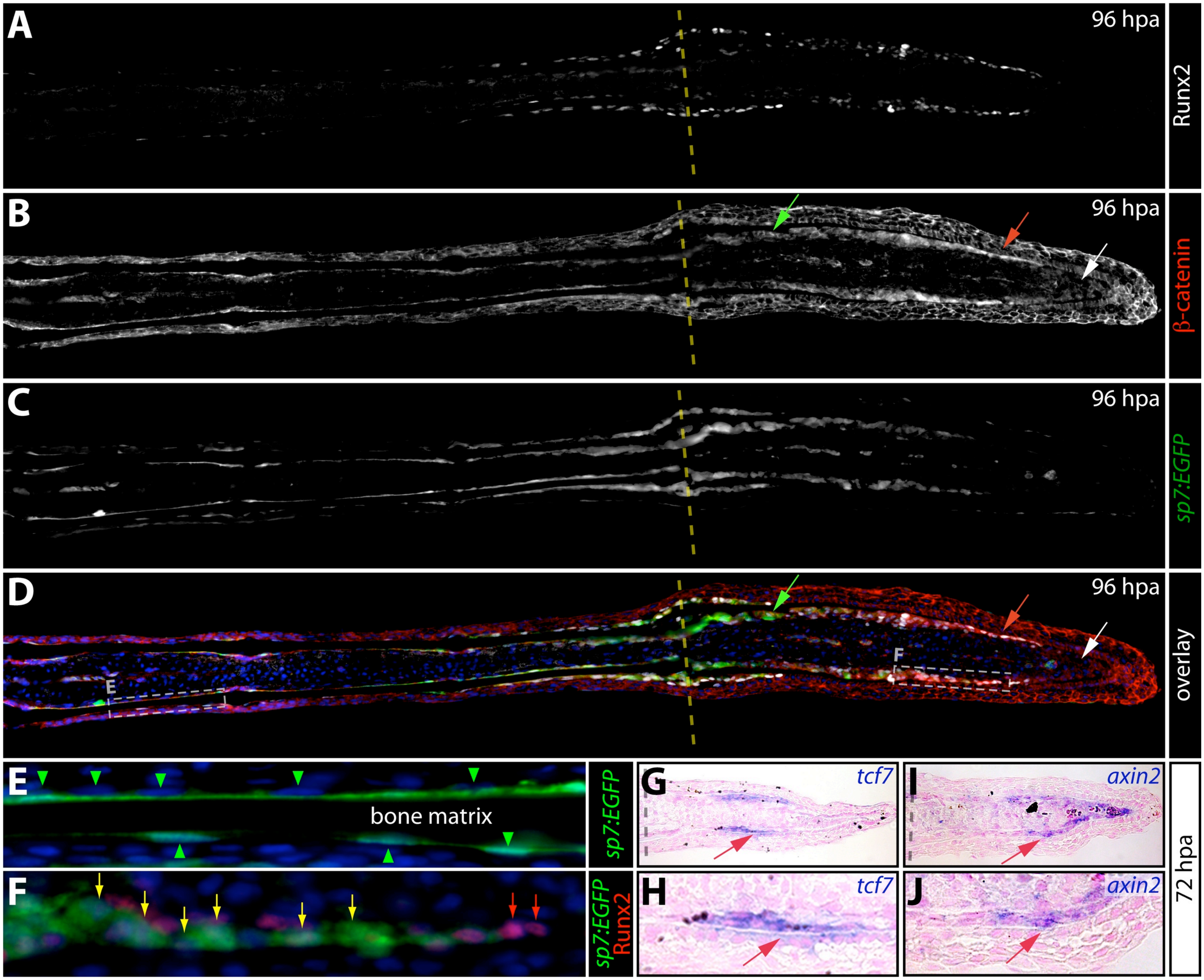

Figure S3, related to Figure 3

Figure S3, related to Figure 3. β-catenin localization in the osteoblast lineage during fin regeneration.

(A-F) Stitched high-resolution widefield epifluorescence images of a fin section from a 4 dpa *Tg(sp7:EGFP)* fish immunostained to show Runx2 expression (A, white), β-catenin localization (B, red), and *sp7:EGFP* reporter activity (C, green). The overlay is shown in (D). Red arrows point to Runx2$^+$ cells containing robust nuclear β-catenin, green arrows indicate *sp7:EGFP*$^+$ with membrane associated β-catenin, and white arrows point to distal blastema cells with low nuclear β-catenin. The dashed yellow lines show the amputation site. (E, F) High magnification images of the regions bounded by the dashed white boxes in (D) to highlight cell shape in non-regenerating (E) vs. regenerating (F) osteoblasts. Green arrowheads indicate elongated non-regenerating *sp7:EGFP*$^+$ epithelial osteoblasts. Red and yellow arrows indicate regenerating distal Runx2$^+$ and Runx2$^+$/*sp7:EGFP*$^+$ cells, respectively, with distinctly rounded morphology. **(G-J)** Detection of *tcf7* (G and H) and *axin2* (I and J) expression by in situ hybridization on sections from 72 hpa fins at low (G and I) and high (H and J) magnification. Red arrows point to specific expression of *axin2* and *tcf7* in distal pre-osteoblasts. The grey dashed line indicates the site of amputation. Shown are representative images indicative of experiments repeated > 6 times.

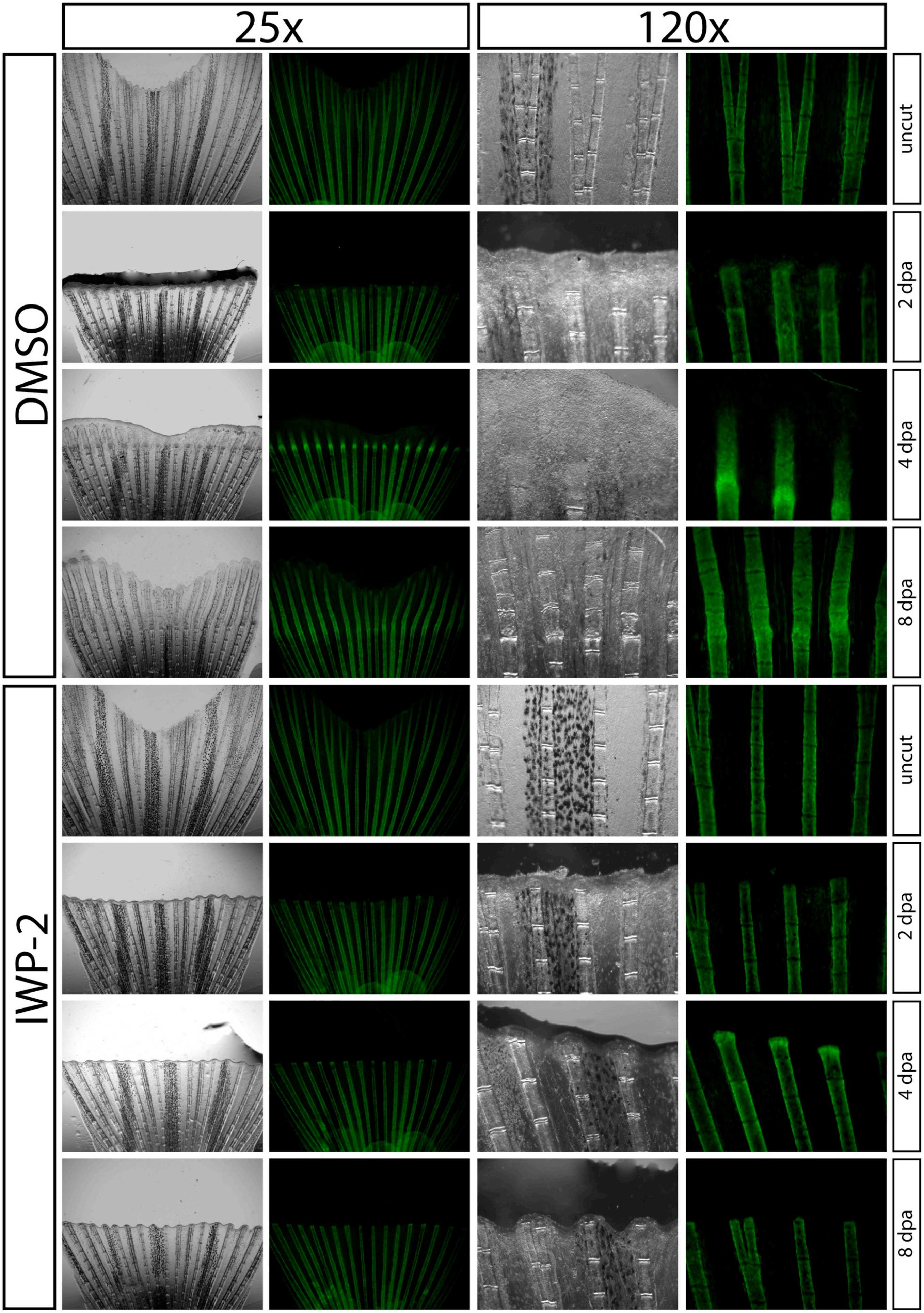

Figure S4, related to Figure 4

Figure S4, related to Figure 4. Wnt production is required for fin regeneration.

Regeneration of caudal fins from *Tg(sp7:EGFP)* fish exposed to DMSO (upper panels) or 10 μM IWP-2 (lower panels) from 0-8 dpa. Each animal is shown before amputation and at 2, 4, and 8 dpa by Rotterman contrast and epifluorescence to visualize *sp7:EGFP* expression (green) in osteoblasts at 25x and at 120x magnification. Shown is one of three fish from each treatment group, within which phenotypes were indistinguishable. The experiment was repeated with three independent fish cohorts.

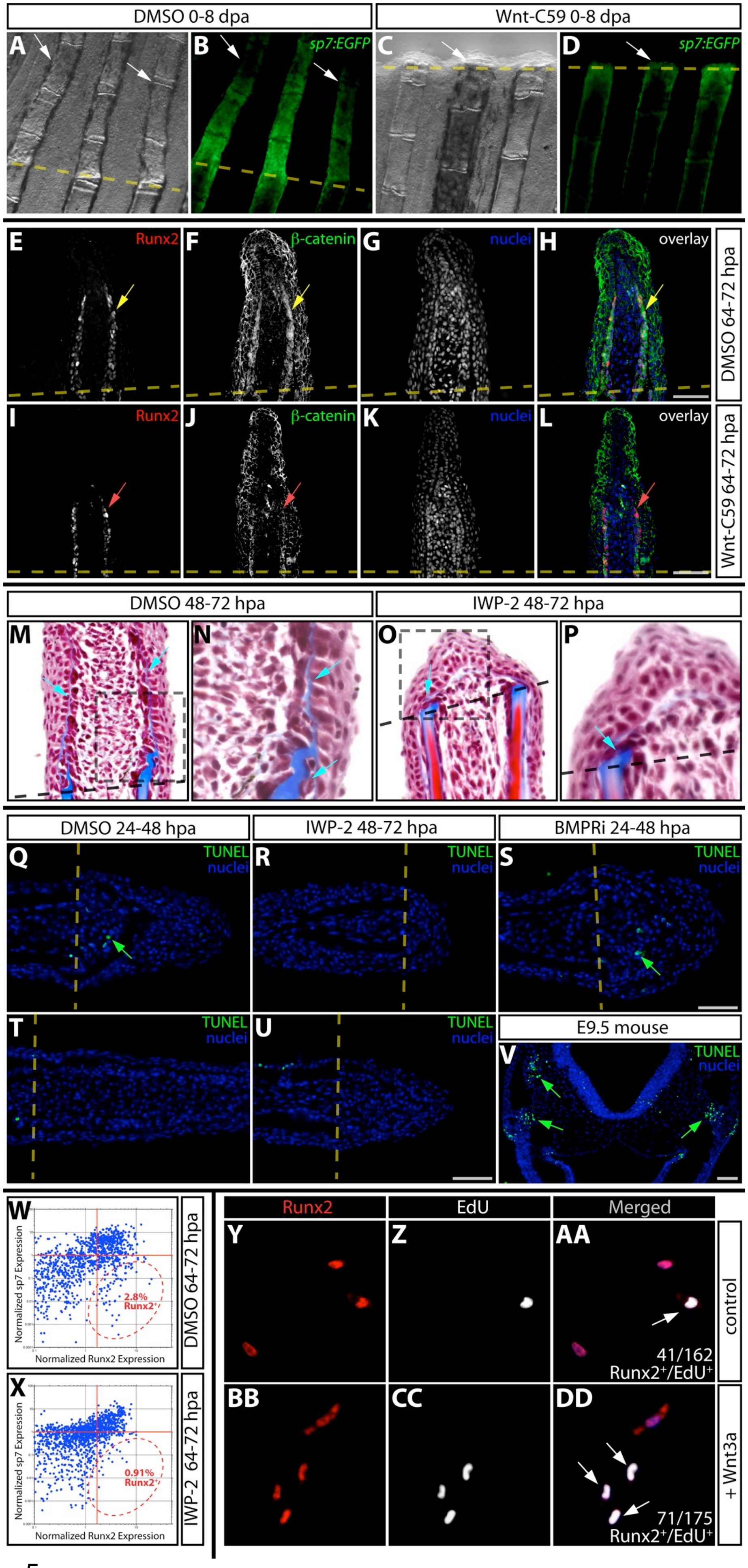

Figure S5, related to Figure 5

Figure S5, related to Figure 5. Wnt/β-catenin signaling is required for fin regeneration by supporting proliferation of Runx2$^+$ pre-osteoblasts.

(A-D) Caudal fin regeneration of *Tg(sp7:EGFP)* zebrafish treated with DMSO (A and B) or 100 nM Wnt-C59 (C and D) from 0-8 dpa. Shown are representative Rotterman contrast (A and C) and epifluorescent images (B and D) from 1 of 3 animals in each treatment group. White arrows point to osteoblasts and the dashed yellow line indicates the amputation site. **(E-L)** Immunostaining to visualize expression of Runx2 (in red) and β-catenin (in green) on fin sections from control (E-H, DMSO 64-72 hpa) and Wnt-C59 treated fish (I-L, 100 nM 64-72 hpa). Yellow arrows point to Runx2$^+$ pre-osteoblasts with accumulation of nuclear β-catenin; red arrows indicate Runx2$^+$ cells with membrane-localized β-catenin. The scale bar represents 50 μm and the dashed line is the plane of amputation. **(M-P)** Trichrome staining of control fins (M and N, DMSO from 48-72 hpa) and IWP-2 exposed fins (O and P, 10 μM from 48-72 hpa) at 72 hpa. Blue staining (Aniline Blue) indicates collagen and other connective tissue (blue arrows). The regions bounded by boxes are shown in higher magnification in the adjacent panel. **(Q-V)** TUNEL staining on fin sections from control (Q and T, DMSO 24-48 hpa), IWP-2 (R and U, 10 μM 48-72 hpa), and BMPRi (S, 5 μM 24-48 hpa) treated fish. A section from an E9.5 mouse is a positive control that serves to demonstrate the distinct labeling of apoptotic cells detected by TUNEL assays. For M-U, a representative ray from one of three fish in each treatment group is shown, with at least three rays examined from each animal. Green arrows indicate TUNEL$^+$ cells. The dashed yellow lines represent the amputation plane. Scale bars are 50 μm. **(W and X)** Scatter plots showing numbers of Runx2$^+$, Runx2$^+$/sp7$^+$ and sp7$^+$ cells in DMSO (W) or IWP-2 treated animals (X, 10 μM 64-72 hpa). Each point on the plots represents a single cell's normalized expression levels for Runx2 (x-axis) and sp7 (y-axis). Scored cells are from at least 10 sections from 4 different animals for each treatment. Regions bounded by the dashed red ellipse are defined as Runx2$^+$ cells and the percentage of Runx2$^+$ cells within the bounded region is shown. **(Y-DD)** Runx2 expression (Y and BB) and EdU incorporation (Z and CC) in cultured primary fin osteoblasts in response to Wnt3a treatment. Overlay images are shown in AA and DD. White arrows indicate Runx2$^+$/EdU$^+$ cells. Results are representative of two independent cell preparations and three independent Wnt3a treatments. In each case $p < 0.05$ (one tailed Fisher's test) comparing the fraction of EdU-incorporated Runx2$^+$ cells in control vs. Wnt3a treated cells.

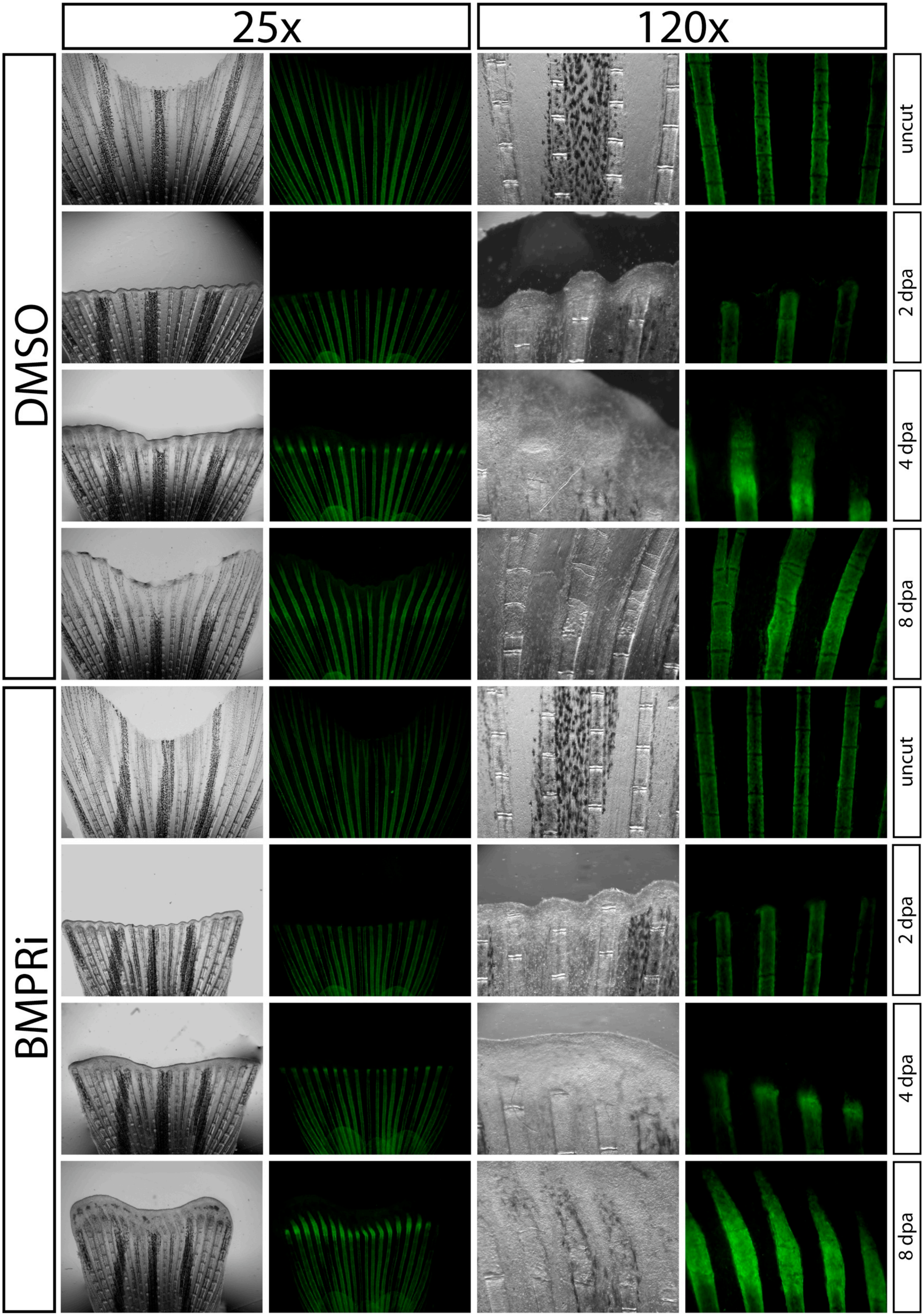

Figure S6, related to Figure 6

Figure S6, related to Figure 6. BMP receptor signaling is required for regeneration.

Regeneration of caudal fins from *Tg(sp7:EGFP)* animals exposed to DMSO (upper panels) or BMPRi (lower panels, 5 μM at 0-8 dpa). Images show the progression of regeneration in an individual fish before amputation and at 2, 4, and 8 dpa using Rotterman contrast and epifluorescence to visualize *sp7:EGFP* expression (green) in osteoblasts at 25x and 120x magnification. One of three fish, which all behaved similarly, from the two treatment groups are shown. The experiment was repeated using three independent sets of animals.

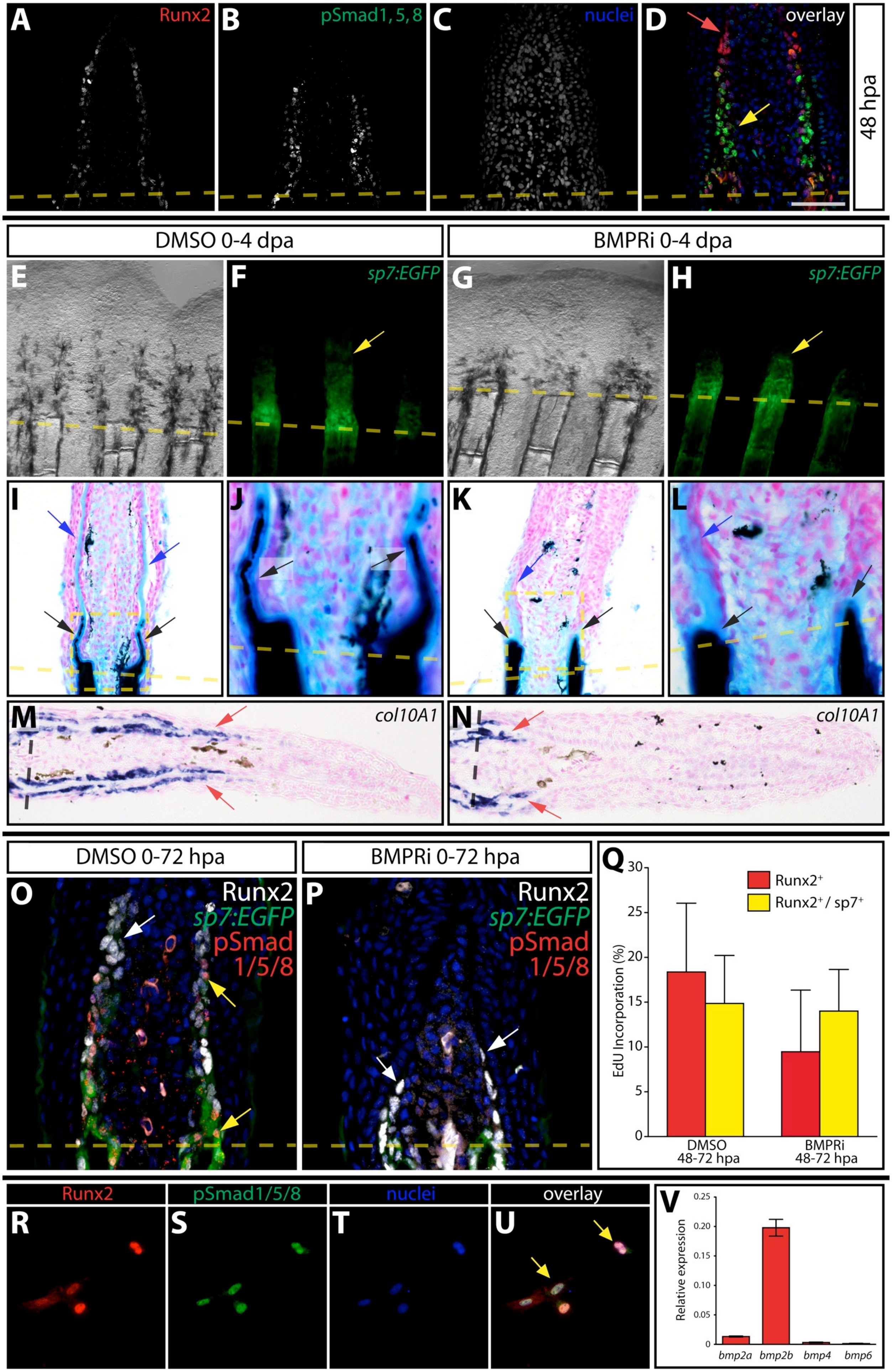

Figure S7, related to Figure 7

Figure S7, related to Figure 7. BMP signaling is essential for bone formation.

(A-D) Immunostaining of a 48 hpa fin section with pSmad 1/5/8 (green) and Runx2 (red) antibodies. The red arrow points to Runx2^{+}/pSmad^{-} pre-osteoblasts localized in the distal blastema. Yellow arrows indicate Runx2^{low}/pSmad^{+} cells. Nuclei are stained with Hoechst (blue) and the dashed line is the amputation plane. The scale bar is 50 μm. **(E-H)** Fin regeneration in *Tg(sp7:EGFP)* animals treated with DMSO (E and F) or BMPRi (G and H, 5 μM) from 0-4 dpa. Shown are representative whole-mount Rotterman contrast (E and G) and epifluorescent images (F and H) from one of the three animals in each group. Yellow arrows mark osteoblasts and the amputation site is indicated with a dashed line. **(I-L)** Von Kossa staining of frozen sections to detect calcified bone in control (I and J) and BMPRi-treated (K and L) fish. Black arrows point to calcified tissue (stained black) distal to the site of amputation (dashed yellow line); blue arrows point to regions rich in mucopolysaccharides (stained blue). The regions bounded by boxes are shown in higher magnification in the adjacent panel. Shown are representative examples from one of three fish for each treatment. (**M and N**) *col10a1* in situ hybridization on DMSO (M) and BMPRi treated (N) fins. Red arrows point to regions of gene expression stained blue. **(O and P)** Immunostaining of sections from *Tg(sp7:EGFP)* animals treated with DMSO (O) or 5 μM BMPRi (P) from 0-72 hpa) to demonstrate Runx2 (white) and *sp7:EGFP* (green) expression and levels of pSmad1/5/8 (red). Hoechst-stained nuclei are shown in blue. White arrows indicate Runx2^{+} cells and yellow arrows show *sp7:EGFP*$^{+}$/pSmad^{+} cells. The dashed yellow line indicates the amputation site. A representative section from one of three fish in each group is shown. Three or more rays were examined for each animal. **(Q)** EdU incorporation in osteoblast sub-types under DMSO or BMPRi conditions. No significant difference was observed ($p > 0.05$, two-tailed Student's t-tests). **(R-U)** Runx2 (red) and pSmad1/5/8 (green) immunostaining of primary cultured fin osteoblasts. Hoechst stained nuclei are shown in blue and the yellow arrows point to Runx2^{+} osteoblasts displaying pSmad1/5/8 immunoreactivity. For each treatment, at least 400 osteoblasts were examined from more than six comparable sections compiled from three different animals. **(V)** qRT-PCR analysis demonstrating relative transcript levels of *bmp2a*, *bmp2b*, *bmp4* and *bmp6* in cultured fin osteoblasts. Equal amounts of template were used for each reaction and relative expression levels were determined by normalizing to *rpl8* expression. Mean relative expression levels from three independent cultures are shown and the error bars indicate one standard deviation.

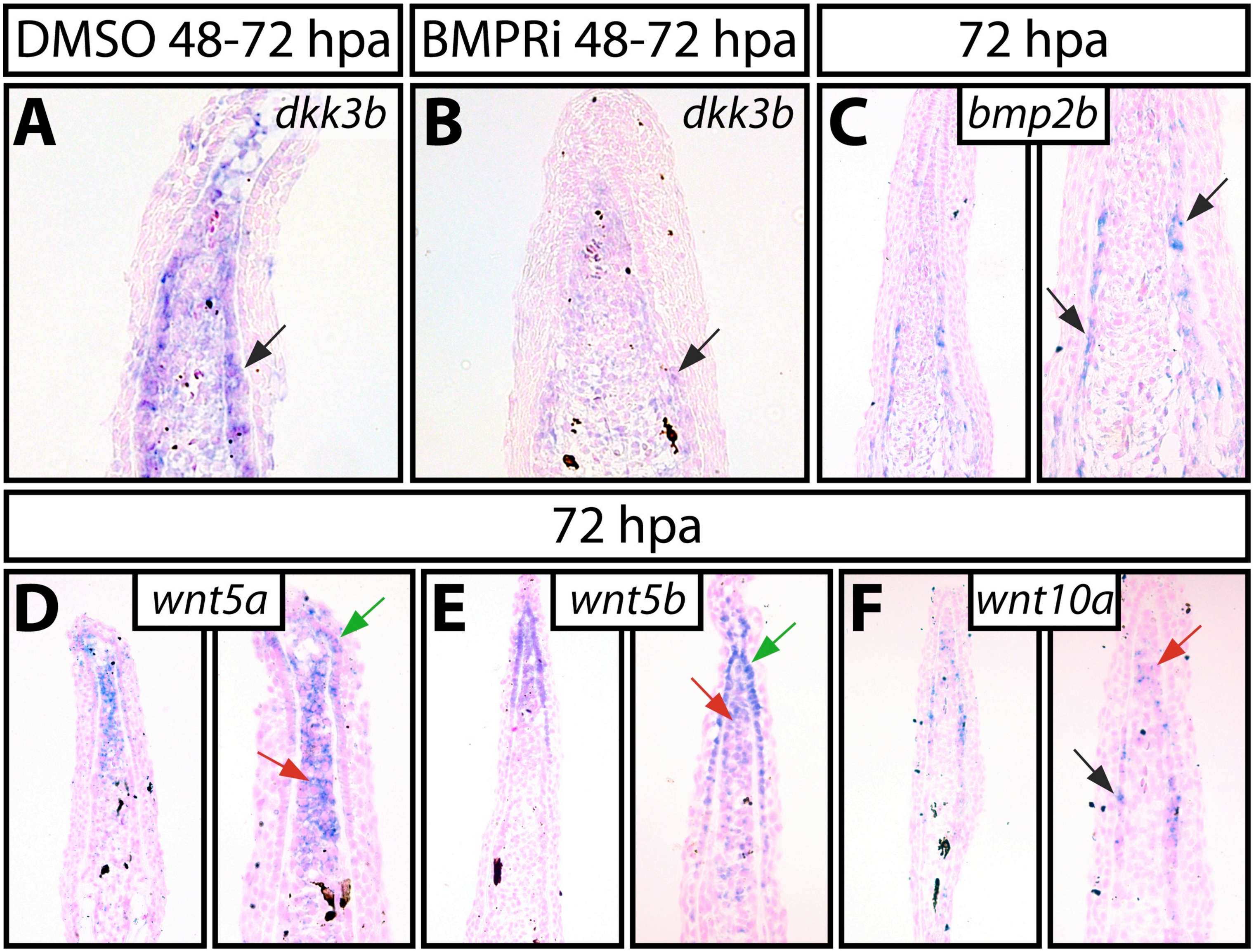

Figure S8, related to Figure 8

Figure S8, related to Figure 8. Localization of Wnt and BMP signals during fin regeneration.

(A and B) In situ hybridization using a *dkk3b* probe on 72 hpa fins from control (A, DMSO 48-72 hpa) and BMPRi (B, 5 μM, 48-72 hpa) treated fish. **(C-F)** Expression of *bmp2b* (C), *wnt5a (D), wnt5b* (E*),* and *wnt10a* (F) by in situ hybridization on paraffin sections from 72 hpa fin, shown at low and high magnification. The black arrows indicate osteoblasts, red arrows point to distal blastema mesenchymal cells, and green arrows mark basal epidermal cells.

SUPPLEMENTAL EXPERIMENTAL PROCEDURES

Immunostaining of sectioned tissue

To prepare paraffin sections, fins were fixed overnight in 4% paraformaldehyde (PFA) in PBS and then washed extensively in PBS. Fins were decalcified for 4 days in 0.5 M EDTA pH 8, which was replaced daily with fresh solution. Following decalcification, fins were rinsed extensively in PBS then dehydrated through an ethanol series and left overnight in 100% ethanol. Ethanol was replaced with xylenes followed by paraffin embedding and sectioning at 7 μm thickness.

For immunostaining, paraffin sections were rehydrated and antigen retrieval was performed in antigen retrieval buffer (1 mM EDTA pH 8, 0.1% Tween-20) for 10' in a pressure cooker. The slides were blocked with PBS/0.1% Tween-20 (PBST) and 10% non-fat dry milk and then incubated overnight at 4°C with primary antibody diluted in blocking buffer. In the case of pSmad 1/5/8 staining, PBST contained 650 mM NaCl during binding of the primary antibody. Slides were washed 3x5' in PBST and 1x30' in PBST containing 650 mM NaCl. Alexa-conjugated secondary antibodies (Invitrogen) were used at 1:1000 diluted in blocking buffer and applied to sections for 1 hour at room temperature, followed by 3 x 5' washes. Nuclei were stained with Hoechst (Invitrogen) in PBST for 10' at room temperature followed by 2 x 5' washes. Slides were mounted using Fluoro-gel (Electron Microscopy Services) and visualized with an Olympus confocal microscope. For confocal imaging, antibody-stained sections were typically analyzed at 20x and 60x magnification. Optical sections were collected and processed using ImageJ software (NIH) with maximum intensity projections generated from z-stacks. Where indicated, single optical sections were used to visualize nuclear-localized β-catenin.

For antibody staining of frozen sections, amputated fins were fixed overnight in 4% PFA/PBS, equilibrated in PBS, cryo-preserved in 30% sucrose/PBS, and frozen in agarose. Cryo-sections (16 μm) were prepared and stored at -20°C until use. Sections were hydrated in PBST and blocked in 10% non-fat dry milk in PBST + 0.1% Triton X-100 for 1-4 hours at room temperature. Subsequent staining and imaging steps followed as for paraffin sections.

Antibodies were sourced and diluted as follows: anti-Runx2 (Santa Cruz Biotechnology, 27-K) 100 ng/ml, anti-sp7 (Santa Cruz Biotechnology, A-13) 20 ng/ml, anti-β catenin (Cell Signaling, #8480) 1:250, anti-phospho Smad 1/5/8 (Cell Signaling, #9511) 1:100, anti-dsRed (Clontech, 632496) 1:200, zns-5 (Zebrafish International Resource Center (ZIRC) 1:5, anti-α-catenin (Genetex, GTX111095) 1:1000, anti-EGFP (Aves Labs, GFP-1020) 1:1000, anti-N-cadherin (BD, 610920) 1:2000.

To quantify osteoblast sub-types, 72 hpa paraffin sections, representing > 6 rays from multiple animals, were stained with Runx2 and sp7 antibodies, and imaged by confocal microscopy. Maximum intensity z-projections were generated using ImageJ (NIH) and processed in Adobe Photoshop. For scatter plot analysis, ImageJ was used to determine relative expression levels of Runx2 and sp7 in individual cells by first normalizing Runx2 and sp7 levels to nuclear staining intensity. Nuclei completely lacking expression of both Runx2 and sp7 were filtered out. Next, the mean normalized expression level was calculated for Runx2 and sp7. The deviation from the mean was determined for each nucleus by dividing the normalized expression level by the mean expression level for both sp7 and Runx2. Results were analyzed and plotted using GraphPad Prism.

Drug treatments and imaging of regenerating zebrafish

For long-term small molecule inhibitor studies, at t = 0, *Tg(sp7:EGFP)b1212* fish from the same clutch were anesthetized in Tricaine and their caudal fins were imaged on a Leica M165FC stereo microscope with epifluorescent illumination. Caudal fins were then amputated with a razor and the animals returned to fish water containing 10 μM IWP-2, 100 nM Wnt-C59, 5 μM BMPRi, or DMSO vehicle. At each indicated time point, animals were anesthetized with Tricaine and re-imaged as described above. Water, containing fresh drug or DMSO, was changed every 24 hours for the duration of the study.

Heat shock studies

Heat shock experiments were performed by amputating fins of control and *Tg(hsp70l:dkk1b-GFP)w32* animals and allowing regeneration to proceed for 48 h. At 48 hpa, fish were

transferred to a water bath and heated to 38°C for 45 minutes. Fish were removed and placed at 28°C. This heat shock regimen was repeated once more at 64 hpa and fins were collected at 72 hpa. For heat-shock studies (n = 3), cohorts of 3 or 4 animals were used and each analyzed at the completion of the study, and images shown are representative examples of each cohort.

In vivo EdU labeling

To analyze cell proliferation, fish were injected intraperitoneally with 12.5 μl of a 1 mg/ml solution of EdU (Invitrogen) in sterile saline 6 hours prior to fin harvesting. EdU was detected on paraffin-sectioned fins using the Click-iT proliferation assay kit (Invitrogen).

In situ hybridization

For in situ hybridization on slides, sections were rehydrated into PBST and then digested for 10'-30' in 2.5 μg/ml Proteinase-K (Invitrogen), re-fixed in 4% PFA/PBS for 30', and washed several times with PBST. Probes were hybridized overnight at 65°C in 5x SSC, 50% formamide, 0.1% Tween-20, 50 μg/ml heparin, and 0.5 mg/ml yeast tRNA. Slides were sequentially washed at 65°C for 30' in 2x SSC, 50% formamide, 0.1% Tween-20, 30' in 2x SSC, 0.1% Tween-20, and 30' in 0.2x SSC, and 0.1% Tween-20. Slides were incubated overnight in anti-digoxigenin (DIG) alkaline phosphatase conjugated antibodies (Roche) diluted 1:2000 in 1% blocking reagent (Roche) and developed using NBT/BCIP (Promega) at 4°C. After color development, slides were stained for 5" in Nuclear Fast Red (Vector Labs), dehydrated, mounted in Permount (Electron Microscopy Services), and imaged on a Leica microscope. For combination fluorescent in situ hybridization-immunostaining, frozen sections were treated for antigen retrieval as described above, followed by in situ hybridization with anti-DIG peroxidase conjugated antibody and developed using the Tyramide Signal Amplification (TSA) system (Perkin Elmer). Following TSA, slides were antibody stained and imaged as described in the Immunostaining section.

For whole mount in situ hybridizations, tissue was hybridized overnight at 65°C in 5x SSC, 50% formamide, 0.1% Tween-20, 50 μg/ml heparin, 0.5 mg/ml yeast tRNA and the appropriate digoxigenin (DIG) labeled riboprobe (Roche), washed and then incubated overnight in α-DIG alkaline phosphatase conjugated antibodies (Roche), and developed with NBT/BCIP (Promega).

In vitro transcription was used to generate digoxigenin (DIG)-labeled (Roche) probes. *Runx2a* (NM_212858), *bmp2b* (NM_131360), *col10a1* (NM_001083827) (Smith et al., 2006), and *sp7* (NM_212863) probes have been described previously (DeLaurier et al., 2010); *axin2* (NM_131561) probe was a gift from M. Westerfield; *GFP* probe was synthesized from pCS2-EGFP plasmid; probes for *dkk3b* (NM_001089545) 5' GGACAATAAAACCGGGAAGAC 3', 5' GGGACACATTTGGAGGTGAC 3'; *twist2* (NM_001005956) 5' ATGGAAGAGAG-TTCTAGCTCTCCCGT 3', 5' CTAGTGGGACGCAGACATCGACC 3'; *twist3* (NM_130985) 5' TAATACGACTCACTATAGGG 3', 5' TTAGTGAGTGGCGGACATGGACC 3'; *tcf7* (NM_001012389) 5' ATGCCGCAGCTGAACGGCGGA 3', 5' TTATCTGCAGGGGCCGCA-CCAGTCCG 3'; *wnt5a* (NM_001079834) 5' GGGCTCCGCGGAGAGCGCCCGGCAGATG 3', 5' TAATACGACTCACTATAGGG 3'; *wnt5b* (BC162984) 5' CTGCTGGCTCCAGCTGGC-CGACTTCC 3', 5' TAATACGACTCACTATAGGG 3'; *wnt10a* (NM_130980) 5' GGGG-AGCGCTTCTCCAAGGACTTCCTGG 3', 5' ACGGCAGTCCATTGCGCAAGGGAGGG 3' were generated by PCR amplification from regenerating caudal fin cDNA using the listed primers with all 3' primers containing a T7 priming site for in vitro transcription by T7 RNA polymerase (Fermentas).

Isolation of osteoblasts from zebrafish caudal fins

Osteoblasts were isolated by first amputating the distal end of approximately 50 adult caudal fins. The fins were then washed in PBS containing 25 μg/ml chloramphenicol and 100 μg/ml kanamycin, diced with a sterile razor, and digested for 1 hour at 30°C in Leibovitz L15 medium (Invitrogen) with 0.25% Trypsin-EDTA (Invitrogen), 25 μg/ml Liberase (Roche), and 1x Antibiotic/Antimycotic cocktail (Invitrogen). 1 ml of Fetal Bovine Serum (FBS, HyClone) was added to stop the digestions and the cells were then centrifuged at 1000 x g and resuspended in osteoblast culture medium (OM) consisting of α-MEM (Invitrogen) supplemented with 10% FBS, 1x Glutamax (Invitrogen), 1x non-essential amino acids (Invitrogen), 1x β-mercaptoethanol (Invitrogen), and 10 ng/ml acidic fibroblast growth factor (aFGF, Peprotech). The cell suspension was then passed through a 40 μm cell strainer (BD Biosciences) and plated onto a 15 cm cell culture dish. Cells were allowed to attach for 20 minutes before the medium and unattached cells were removed. Adhered cells were gently washed 3 times with PBS and osteoblast-enriched cells were recovered with 3 ml 0.25% Trypsin-EDTA for 5 minutes,

followed by adding 3 ml of OM. The cells were centrifuged as above, washed, resuspended in OM, and plated onto multi-well dishes containing coverslips coated with rat-tail collagen (Invitrogen). Cells were cultured at 30°C in 5% CO_2 and 5% O_2. To determine cell purity at 4 days post isolation, cells were fixed, immunostained with Runx2 and sp7 antibodies, and scored.

Treatment and staining of zebrafish osteoblasts

To assay β-catenin localization, zebrafish osteoblasts were maintained in OM for 48 hours before addition of either 300 nM LDN193189 (BMPRi) and/or 40 ng/ml recombinant mouse Wnt3a (R&D Systems) for 24 hours. Wnt3a dosage was determined according to the manufacturer's recommendation and dose-response pilot studies. Cells were then washed twice in PBS and fixed at room temperature for 20 minutes in 4% PFA in PBS. Cells subsequently were permeabilized in 0.2% Triton X-100 in PBS for two minutes and blocked with 5% Normal Goat Serum (NGS, MP Biomedicals) in PBS for 30 minutes. Primary antibodies used were anti-EGFP 1:1000, anti-sp7 20 ng/ml, anti-Runx2 100 ng/ml, anti-β-catenin 1:250, and anti-phospho-SMAD 1/5/8 1:500 and were applied for 1 hour at room temperature in blocking buffer. To visualize nuclei, the slides were incubated with 2 μg/ml Hoechst solution in PBS for 10-15 minutes. Coverslips were mounted with Fluoro-gel mounting medium (Electron Microscopy Services).

To quantify nuclear β-catenin levels, cells first were imaged at 40x magnification. Then, the ratio of nuclear signal as a percentage of the total β-catenin signal for individual cells was determined using FIJI software. To normalize for background signal, we imaged cells treated with secondary antibody alone. The same acquisition exposure-time parameters were used to capture images for all conditions. For fluorescence intensity measurements the following formula was used:

Corrected total cell fluorescence (CTCF) = Integrated Density – (Area of selected cell x Mean fluorescence of background)

Corrected nuclear fluorescence (CNF) = Integrated Density of Nucleus– (Area of nucleus x Mean fluorescence of background)

Percent nuclear β -catenin = (CNF/CTCF) x 100

The mean percent nuclear β-catenin from at least 25 cells from each treatment was plotted with error bars representing the standard deviation.

To examine effects of Wnt3a on proliferation, osteoblasts were cultured in OM media with either 40 ng/ml of recombinant mouse Wnt3a (R&D systems) or an equivalent concentration of PBS. Cells received fresh OM media, with or without Wnt3a, every 24 hours. After 48 hours in culture, 10 μM EdU was added and the cells were fixed in 4% PFA 12 hours later. EdU detection was performed using the Click-iT proliferation assay kit (Invitrogen). Cells were then stained with anti-Runx2 antibodies. The fraction of proliferating $Runx2^+$ osteoblasts was counted for each condition and a one-tailed Fisher's test was used to determine statistically significant differences between control and Wnt3a treated cells.

Quantitative RT-PCR

For in vitro studies, cells were isolated from caudal fins as described above. After 48 hours in culture, cells in triplicate wells were treated with 300 nM LDN193189 (BMPRi) or 0.003% DMSO as a control. After 24 hours of treatment, the media was changed, and fresh BMPRi or DMSO was added. After 96 hours in culture (48 hours of treatment), cells were washed 3x in PBS. RNA was extracted using TRIzol reagent (Invitrogen) according to the manufacturer's protocol. To analyze changes in gene expression upon in vivo inhibition of BMP signaling, 6 groups of 3 or 4 animals from the same clutch were used: 3 cohorts were treated with 5 μM BMPRi from 48 to 96 hours after amputation; similarly, 3 control cohorts were treated with the same volume of DMSO. At 96 hpa, regenerating fin tissue was dissected and RNA was extracted using TRIzol reagent (Invitrogen). cDNA was generated from total RNA using Superscript III reverse transcriptase and Oligo-dT(20) primers (Invitrogen) following the manufacturer's instructions. qPCR was performed using KAPA SYBR qPCR Master Mix (Kapa Biosystems). Relative mRNA expression levels between untreated and treated cohorts were determined using the ΔΔCt method in relation to *rpl8* mRNA abundance.

Primer sequences used for qPCR were:
runx2a (NM_212858) 5' ACGGTAATGGCTGGAAATGA 3', 5' GTCCGTCCACTGTGACC-TTT 3'; *runx2b* (NM_212862) 5' AGCTTCACCCTGACGATTACA 3', 5' CCAGTTCACTGA-GACGGTCA 3'; *sp7* (NM_212863) 5' TCCAGACCTCCAGTGTTTCC 3', 5' ATGGACATC-CCACCAAGAAG 3'; *dkk1a* (XM_001339705) 5' ACATCCCAGGAGAACCACAG 3', 5' AAACTTGTCCCTCTGTCAGCA 3'; *dkk1b* (NM_131003) 5' TCCTAAAAGAGGGCCAG-

GTC 3', 5' TCCCTCGACTCAAGTCTGCT 3'; *dkk2* (NM_001111209) 5' ATCTCAGAGAGC-GCCTTGTC 3', 5' GCTTGCAGATTTTGGTCCAG 3'; *dkk3b* (NM_001089545) 5' GGACAA-TAAAACCGGGAAGAC 3', 5' GGGACACATTTGGAGGTGAC 3'; *rpl8* (NM_200713) 5' CCGAGACCAAGAAATCCAGA 3', 5' GAGGCCAGCAGTTTCTCTTG 3'; *bmp2a* (NM_131359.1) 5' ATCAGGAGCTTCCA-CCATGA 3, 5' TGAACGTTAATGCGGTGAAA 3'; *bmp2b* (NM_131360.1) 5' CTGAAA-ACGATGACCCGAAC 3', 5' AACTGCTGCGTTGT-TTTTCC 3'; *bmp4* (NM_131342.2) 5' AGCAGTGCCTTCAAAGGTTG 3', 5' CATGGGGAA-ACAGTCCATGT 3'; *bmp6* (NM_001013339.1) 5' GGGTTGGCTGGAGTTTGAC 3', 5' ACGGACTTCGCTCACTTTGA 3'.

Mosaic analysis

Homozygous *Tg(Xla.ef1a1-actb2:LOXP-LOX5171-FRT-F3-EGFP,mCherry)vu295a* fish were crossed with heterozygous *Tg(dusp6:CreERT2,myl7:ECFP)b1230* animals and the resulting clutch was treated with 1 μM tamoxifen (Sigma-Aldrich) starting at 30-50% epiboly until 48 hours post fertilization (hpf). Animals containing mCherry$^+$ cells were selected and reared to adulthood. For osteoblast mosaic experiments, adult *Tg(Xla.ef1a1-actb2:LOXP-LOX5171-FRT-F3-EGFP,mCherry); Tg(dusp6:CreERT2,myl7:ECFP)* animals were selected that had caudal fins containing isolated mCherry$^+$ osteoblast mosaics. Their fins were amputated, harvested at 24, 48, or 72 hpa, and processed for immunostaining with Runx2 and dsRed antibodies on either frozen (24 and 48 hpa) or paraffin (72 hpa) sections.

TUNEL staining

A Click-iT TUNEL kit (Invitrogen) was used to detect apoptotic cells on paraffin sections from DMSO and drug treated caudal fins as directed by the manufacturer. As a positive control, sections of E9.5 mouse embryos were used. After TUNEL labeling, slides were stained with Hoechst to label nuclei and then imaged.

Histological Analysis

For Masson's trichome staining, paraffin sections were rehydrated though a xylenes/ethanol/water series, stained using a kit as directed by the manufacturer (American Master Tech), and imaged by bright field microscopy. For Von Kossa staining, non-decalcified

16 μm frozen sections were rehydrated and stained in 1% silver nitrate solution in a Coplin jar under ultraviolet light for 20'. Un-reacted silver was removed with a 5' treatment with 5% sodium thiosulfate. Slides were then stained with Alcian blue for 30' followed by a 5' Nuclear Fast Red stain (Vector Labs), dehydrated in ethanol, cleared in xylenes, mounted, and imaged by bright field microscopy.
